# Living alone and mental health: parallel analyses in UK longitudinal population surveys and electronic health records prior to and during the COVID-19 pandemic

**DOI:** 10.1136/bmjment-2023-300842

**Published:** 2023-08-10

**Authors:** Eoin McElroy, Emily Herrett, Kishan Patel, Dominik M Piehlmaier, Giorgio Di Gessa, Charlotte Huggins, Michael J Green, Alex S F Kwong, Ellen J Thompson, Jingmin Zhu, Kathryn E Mansfield, Richard J Silverwood, Rosie Mansfield, Jane Maddock, Rohini Mathur, Ruth E Costello, Anthony Matthews, John Tazare, Alasdair Henderson, Kevin Wing, Lucy Bridges, Sebastian Bacon, Amir Mehrkar, OpenSAFELY Collaborative, Richard John Shaw, Jacques Wels, Srinivasa Vittal Katikireddi, Nish Chaturvedi, Laurie A Tomlinson, Praveetha Patalay, Longitudinal Health and Wellbeing Collaborative

**Affiliations:** 1 School of Psychology, Ulster University, Coleraine, UK; 2 Epidemiology and Population Health, London School of Hygiene and Tropical Medicine, London, UK; 3 MRC Unit for Lifelong Health and Ageing, University College London, London, UK; 4 Bennett Institute for Applied Data Science, Nuffield Department of Primary Care Health Sciences, University of Oxford, Oxford, UK; 5 Strategy and Marketing, University of Sussex Business School, Brighton, UK; 6 Epidemiology and Public Health, University College London, London, UK; 7 Centre for Genomic and Experimental Medicine, University of Edinburgh, Edinburgh, UK; 8 MRC/CSO Social and Public Health Sciences Unit, University of Glasgow, Glasgow, UK; 9 MRC Integrative Epidemiology Unit, University of Bristol, Bristol, UK; 10 Division of Psychiatry, University of Edinburgh, Edinburgh, UK; 11 Department of Twin Research & Genetic Epidemiology, King's College London, London, UK; 12 Centre for Longitudinal Studies, University College London, London, UK; 13 Centre for Primary Care, Wolfson Institute of Population Health, Queen Mary University of London, London, UK; 14 Unit of Epidemiology, Institute of Environmental Medicine, Karolinska Institute, Stockholm, Sweden

**Keywords:** anxiety disorders, COVID-19, psychiatry

## Abstract

**Background:**

People who live alone experience greater levels of mental illness; however, it is unclear whether the COVID-19 pandemic had a disproportionately negative impact on this demographic.

**Objective:**

To describe the mental health gap between those who live alone and with others in the UK prior to and during the COVID-19 pandemic.

**Methods:**

Self-reported psychological distress and life satisfaction in 10 prospective longitudinal population surveys (LPSs) assessed in the nearest pre-pandemic sweep and three periods during the pandemic. Recorded diagnosis of common and severe mental illnesses between March 2018 and January 2022 in electronic healthcare records (EHRs) within the OpenSAFELY-TPP.

**Findings:**

In 37 544 LPS participants, pooled models showed greater psychological distress (standardised mean difference (SMD): 0.09 (95% CI: 0.04; 0.14); relative risk: 1.25 (95% CI: 1.12; 1.39)) and lower life satisfaction (SMD: −0.22 (95% CI: −0.30; −0.15)) for those living alone pre-pandemic. This gap did not change during the pandemic. In the EHR analysis of c.16 million records, mental health conditions were more common in those who lived alone (eg, depression 26 (95% CI: 18 to 33) and severe mental illness 58 (95% CI: 54 to 62) more cases more per 100 000). For common mental health disorders, the gap in recorded cases in EHRs narrowed during the pandemic.

**Conclusions:**

People living alone have poorer mental health and lower life satisfaction. During the pandemic, this gap in self-reported distress remained; however, there was a narrowing of the gap in service use.

**Clinical implications:**

Greater mental health need and potentially greater barriers to mental healthcare access for those who live alone need to be considered in healthcare planning.

**WHAT IS ALREADY KNOWN ON THIS TOPIC**

- Households with one individual are an increasing demographic, comprising over a quarter of all households in the UK in 2021. However, the mental health gap between those who live alone compared with those who live with others is not well described and even less is known about the relative gaps in need and healthcare-seeking and access. The pandemic and associated restrictive measures further increased the likelihood of isolation for this group, which may have impacted mental health.

**WHAT THIS STUDY ADDS**

- We present comprehensive evidence from both population-based surveys and electronic health records regarding the greater levels of psychological distress symptoms, lower life satisfaction and in recorded diagnoses for common (anxiety, depression) and less common (obsessive compulsive disorder, eating disorders, serious mental illnesses) mental health conditions for people living alone compared with those living with others. We present this information for both before and during the COVID-19 pandemic. The different data sources provide information on both population-levels of distress and wellbeing and patterns in healthcare seeking and diagnosis.

**HOW THIS STUDY MIGHT AFFECT RESEARCH, PRACTICE OR POLICY**

- Our analyses indicate that a range of mental health outcomes and conditions are more common among those who live alone compared with those who live with others. Although levels of reported distress increased for both groups during the pandemic, healthcare-seeking dropped in both groups, and the rates of healthcare-seeking among those who live alone converged with those who live with others for common mental health conditions. This could suggest greater barriers to treatment access among those who live alone in this period. The findings have implications for mental health service planning and efforts to reduce barriers to treatment access, especially for individuals who live on their own.

## Introduction

More people than ever are living alone.^1^ For instance, recent estimates from the UK suggest that over 25% of households have just one resident, with considerable regional variations seen within the country (eg, 25.8% in London and 36.0% in Scotland).^2^ Projections suggest that the total number of lone households in the UK could rise from the current estimates of 7.7 million to 10.7 million households by 2039.^3^ Studies consistently demonstrate that people who live alone are more likely to experience common^4 5^ and severe mental illness (SMI),^6 7^ along with increased self-harm and suicide rates.^8^

The COVID-19 pandemic, at least temporarily, changed the context of living alone. Attempts to curb the spread of the virus via lockdowns led to extended periods of greatly reduced in-person social interactions. People living alone during lockdown saw the greatest declines in face-to-face contact,^9^ and research suggests that alternative means of social interaction (eg, telephone, video chat) may not protect mental health to the same degree as in-person interaction.^10 11^ Consequently, those living alone may have experienced a disproportionate increase in mental health difficulties as a result of enforced isolation during the pandemic. However, studies to date, using both cross-sectional and longitudinal samples, have reported mixed findings, with some suggesting no widening of the distress gap between the two groups,^12–14^ and others noting a steeper rise in psychological distress for people who lived alone in the first months of the pandemic.^15–19^

The existing evidence, however, suffers from several key limitations. First, the majority of studies have focused only on the short-term impact of the pandemic, and few UK studies have evidenced the extent of the gap pre-pandemic and whether the mental health gap between people living alone and living with others changed as the pandemic became prolonged. Second, most studies have only considered a narrow range of mental health outcomes, particularly high-prevalence disorders such as depression and anxiety. To our knowledge, little attention has been given to more severe mental health outcomes, such as eating disorders, obsessive compulsive disorder (OCD), psychoses and self-harm. Finally, the majority of studies have used convenience samples, which may not generalise to the UK population. The combination of limited mental health outcomes studied and convenience sampling makes it difficult to assess if living alone continued to be associated with poor mental health during the COVID-19 pandemic.

Establishing the impact of COVID-19 on population mental health requires rich longitudinal data, with assessments for periods both pre-COVID-19 and during the pandemic. In the UK, such data are available in ongoing population-based longitudinal studies and electronic health records (EHRs). The longitudinal population studies (LPSs) include regular assessments of the same individuals and have a wealth of information on living arrangements and mental health prior to and during the pandemic. However, they are limited to small samples (relative to EHRs), typically focus on common mental health symptoms and may contain only a limited number of assessments.^20^ EHRs overcome many of these limitations, in this case allowing us to study the association between living status and mental health outcomes at scale, providing the statistical power and precision to explore the impact of the pandemic on more serious but lower-prevalence mental illnesses (eg, psychosis, OCD).^21^ However, EHRs have other limitations as they do not capture psychosocial phenomena such as loneliness (subjective feelings of inadequate social relations) and social support (having people to turn to when in psychological or material need). Furthermore, they capture information only for those who seek healthcare and report symptoms, which may be problematic given that there are many inequalities in who accesses health services for mental health difficulties.^22^ While EHRs give an indication of mental health service use and healthcare access, relying solely on this data source is likely to underestimate mental health problems due to under-reporting of common mental health problems, especially for certain subgroups of the population, and particularly during the pandemic, due to closure or reduction of services.

Consequently, this study aimed to provide high-quality evidence on the association between living alone and mental health outcomes before and during the COVID-19 pandemic by estimating these associations in EHRs and estimating and pooling results from 10 UK longitudinal population-based studies, thus adding robustness and information on both need and healthcare utilisation, while balancing the strengths and weaknesses of each data source. Our specific objectives were as follows:

### Objective 1

To describe the gap between those who live alone versus those who live with others, in a range of mental health outcomes in both population-based surveys and recorded within EHRs.

### Objective 2

To examine whether there was an effect of the pandemic on the mental health gap between those who live alone and those who live with others by examining pre-pandemic and during pandemic levels of distress in the population and frequency of healthcare contacts due to mental ill-health. We also examine whether the association between living alone and mental health pre-pandemic and during the pandemic differed by sociodemographic subgroups and according to feelings of loneliness.

## Methods

### Participants

The population of interest was community-dwelling adults living in private households.

#### Longitudinal population surveys

Data were drawn from 10 ongoing longitudinal population studies in the UK that had data available prior to and during the COVID-19 pandemic. The details of each study (design, sample frames, current age range, timing of the most recent pre-pandemic and COVID-19 surveys, response rates and analytical sample size) are presented in table 1. Five of these studies were age-homogeneous birth cohorts and the remaining five covered different age ranges. Ethics statements and data availability information for each study are available in online supplemental file 1, and funding statements are available in online supplemental file 2. Further details on the cohorts are provided in online supplemental file 3.

10.1136/bmjment-2023-300842.supp1 Supplementary data

**Table 1:** Details of each longitudinal population survey including design, time points, response rates, measures used and % living alone

Study population	Design and sample frame	2020 age range in years	Most recent pre-pandemic survey	Details of COVID-19 surveys (response rate)	Psychological distress measure used	Analytical N	% living alone
NS: Next Steps, formerly known as Longitudinal Study of Young People in England	Sample recruited via secondary schools in England at around age 13 with regular follow-up surveys thereafter.	29–31	2015	Three surveys: May (20.3%); Sep–Oct (31.8%); Feb–Mar (29%)	General Health Questionnaire 12 (GHQ-12)	1262	19.3
BCS70: British Cohort Study 1970	Cohort of all children born in Great Britain (ie, England, Wales & Scotland) in 1 week in 1970, with regular follow-up surveys from birth.	50	2016	Three surveys: May (40.4%); Sep–Oct (43.9%); Feb–Mar (40%)	9-item Malaise Inventory	2793	8.8
NCDS: National Child Development Study	Cohort of all children born in Great Britain (ie, England, Wales & Scotland) in 1 week in 1958, with regular follow-up surveys from birth.	62	2013	Three surveys: May (57.9%); Sep–Oct (53.9%); Feb–Mar (52%)	9-item Malaise Inventory	3772	9.8
NSHD: National Survey of Health and Development	Cohort of all children born in Great Britain (ie, England, Wales & Scotland) in 1 week in 1946, with regular follow-up surveys from birth.	74	2015	Three surveys: May (68.2%); Sep–Oct (61.5%); Feb–Mar (90%)	GHQ-12	1640	21.7
ALSPAC G1: Avon Longitudinal Study of Parents and Children-Generation 1	Cohort of children born in the South West of England between April 1991 and Dec 1992, with regular follow-up questionnaires from birth.	27–29	2017–2018	Three surveys: April 2020 (19%); June 2020 (17.4%); December 2020 (26.4%)	Short Mood and Feelings Questionnaire (SMFQ)	2252	6.2
USoc: Understanding Society: the UK Household Longitudinal Survey	A nationally representative longitudinal household panel study, based on a clustered-stratified probability sample of UK households, with all adults aged 16+ in chosen households surveyed annually.	16–96	2018–2019	Eight surveys: April (40.3%); May (33.6%); Jun (32.0%); July (31.2%); Sep (29.2%); Nov (27.3%); Jan 2021 (27.2%); Mar 2021 (28.8%)	GHQ-12	12 270	18.3
ELSA: English Longitudinal Study of Ageing	A nationally representative population study of individuals aged 50+ living in England, with biennial surveys and periodical refreshing of the sample to maintain representativeness.	52–90+	2018–2019	Two surveys: Jun–July (75%); Nov–Dec (73%)	Centre for Epidemiological Studies–Depression	5471	21.2
GS: Generation Scotland: the Scottish Family Health Study	A family-structured, population-based Scottish cohort, with participants aged 18–99 recruited between 2006 and 2011.	27–100	2006–2011	Three surveys: April–Jun 2020 (21.3%); Jul–Aug 2020 (15.4%); Feb 2021 (14.3%)	Patient Health Questionnaire 9 or 8	2984	9.1
TwinsUK: the UK Adult Twin Registry	A cohort of volunteer adult TwinsUK (55% monozygotic and 43% dizygotic) from around the UK who were sampled between 18 and 101 years of age.	22–96	2017–2018	Three surveys: April (64.3%); July (77.6%); November (76.1%)	Hospital and Anxiety Depression Scale	2327	20.6
ALSPAC G0: Avon Longitudinal Study of Parents and Children-Generation 0 (Parents)	Parents of the ALSPAC (G1) cohort described above, treated as a separate age-heterogeneous study population (original parents).	45–81	2011–2013	Three questionnaires: April 2020 (12.4%; June 2020 (12.2%); December 2020 (14.3%)	SMFQ	2773	7.4

To be included in the analytical sample, participants were required to have: (1) information on living status (alone vs not alone) from early 2020 (the first COVID-19 survey); (2) valid data on our primary outcome (psychological distress) pre-pandemic and during at least one COVID-19 data sweep; and (3) valid data on a key minimum set of covariates (sex, age, ethnicity, education, UK nation, urbanicity, occupational class, housing tenure, chronic illness; see online supplemental file for details of covariates in each study). Where possible, studies were weighted to be representative of their target population, accounting for sampling design and differential non-response.

#### Electronic health records

EHRs managed by the general practice (GP) software provider TPP were accessed through OpenSAFELY (https://www.opensafely.org/). The OpenSAFELY platform covers approximately 24 million adults and children currently registered with primary care practices in England using TPP SystmOne software, linked to hospitalisation and mortality data. OpenSAFELY allows data access to researchers through a Trusted Research Environment. Primary care records included coded diagnoses, prescriptions and physiological measures recorded as part of routine care. Free-text data are not available in OpenSAFELY. Data for this study were extracted each month between 1 March 2018 and 31 January 2022 for all adults (aged 18 years or older) who were registered with a GP using TPP SystmOne, had at least 3 months of continuous registration prior to that month, were registered with a TPP practice as of 1 February 2020, and had a valid address or postcode. Individuals who met the inclusion criteria were included until the first of: death, de-registration from primary care practice or the end of the study period. Those living in households with more than 15 individuals or with missing age, sex, Sustainability and Transformation Partnership (STP) region (a geographical area used in National Health Service administration), or Index of Multiple Deprivation (IMD) were excluded to ensure high data quality without including institutionalised living facilities (eg, care homes) and omit any institutional effects. Further details of OpenSAFELY are presented in online supplemental file 6.

Study size was based on all participants in each cohort for LPS meeting inclusion criteria of data availability (table 2), and the number of individuals registered with TPP and meeting the inclusion criteria for EHRs.

**Table 2:** Characteristics of patients in OpenSAFELY-TPP in January 2020

	Total population	Not living alone	Living alone
	N (%)	N (%)	N (%)
Total population in 2020	15 983 045	12 560 414	3 422 631
Age group			
18–40 years	5 759 423 (36.0)	4 825 262 (38.4)	934 161 (27.3)
41–60 years	5 272 785 (33.0)	4 325 383 (34.4)	947 402 (27.7)
61–80 years	3 966 060 (24.8)	2 905 601 (23.1)	1 060 459 (31.0)
>80 years	984 777 (6.20)	504 168 (4.0)	480 609 (14.0)
Sex			
Female	8 092 393 (50.6)	6 347 467 (50.5)	1 744 926 (51.0)
Male	7 890 652 (49.4)	6 212 947 (49.5)	1 677 705 (49.0)
Living status			
Living alone	3 422 631 (21.4)	–	3 422 631 (100)
Not living alone	12 560 414 (78.6)	12 560 414 (100)	–
Ethnicity			
White	11 094 879 (85.6)	8 601 261 (84.7)	2 493 618 (89.0)
Mixed	177 748 (1.4)	139 583 (1.4)	38 165 (1.4)
Asian	996 754 (7.7)	882 723 (8.7)	114 031 (4.1)
Black	351 631 (2.7)	276 676 (2.7)	74 955 (2.7)
Other	333 836 (2.6)	251 884 (2.5)	81 952 (2.9)
Missing	3 028 197	2 408 287	619 910
IMD quintile			
1 (least deprived)	3 161 810 (19.8)	2 455 384 (19.5)	706 426 (20.6)
2	3 220 734 (20.2)	2 485 660 (19.8)	735 074 (21.5)
3	3 410 618 (21.3)	2 650 307 (21.1)	760 311 (22.2)
4	3 276 903 (20.5)	2 596 043 (20.7)	680 860 (19.9)
5 (most deprived)	2 912 980 (18.2)	2 373 020 (18.9)	539 960 (15.8)
Region			
East	3 681 521 (23.0)	2 998 303 (23.9)	683 218 (20.0)
East Midlands	2 809 793 (17.6)	2 258 073 (18)	551 720 (16.1)
London	1 195 246 (7.5)	869 491 (6.9)	325 755 (9.5)
North East	775 155 (4.8)	608 067 (4.8)	167 088 (4.9)
North West	1 391 571 (8.7)	1 082 407 (8.6)	309 164 (9.0)
South East	1 099 976 (6.9)	839 697 (6.7)	260 279 (7.6)
South West	2 196 573 (13.7)	1 685 418 (13.4)	511 155 (14.9)
West Midlands	631 974 (4.0)	495 883 (3.9)	136 091 (4.0)
Yorkshire and the Humber	2 201 236 (13.8)	1 723 075 (13.7)	478 161 (14.0)
Household size			
>10	94 169 (0.6)	94 169 (0.7)	–
≤10	15 888 876 (99.4)	12 466 245 (99.3)	3 422 631 (100)
Household members			
All at a TPP practice	13 773 345 (86.2)	10 856 742 (86.4)	2 916 603 (85.2)
Not all at a TPP practice	2 209 700 (13.8)	1 703 672 (13.6)	506 028 (14.8)
Advised to shield	1 359 711 (8.5)	952 960 (7.6)	406 751 (11.9)
Previous mental illness	2 193 540 (13.7)	1 661 537 (13.2)	532 003 (15.5)

TPP practices are those using TPP SystmOne software.

IMD, Index of Multiple Deprivation.

### Exposure: living alone or not

Our primary exposure was living status derived in the longitudinal studies at the start of the pandemic between April and June 2020 (this time point was used as it was consistent across LPSs and housing status changes later in the pandemic might be affected by mental health) and in EHRs just before the start of the pandemic on 1 February 2020 (identification of the number of individuals in each household was done by TPP independently of the present study). Briefly, this was done by building a table of addresses for every person in the OpenSAFELY-TPP platform and setting household size based on the number of people with the same address. Full methods are described by Wing *et al*.^23^ Participants were defined as living alone if they had a household size of 1, and were defined as not living alone if they had any value >1.

### Mental health outcomes

In the LPS, our primary outcomes were measures of psychological distress (ie, symptoms of depression and anxiety) and life satisfaction (see table 1 for specific scales). These were assessed in the most recent pre-pandemic sweep of each cohort (T0; see table 1 for specific years) and three periods during the pandemic: (1) a period roughly corresponding to the first lockdown (April–June 2020, T1); (2) a period between July and October 2020, when initial restrictions were eased (T2); and (3) a final period (T3) covered November 2020–March 2021, when infection rates rose again, necessitating a second national lockdown. The continuous scales were standardised across the assessment waves within each cohort. We also derived binary outcome variables based on established cut-offs reflecting probable disorder (see online supplemental file 4 for criteria). Our secondary outcome, life satisfaction, was measured using questions, such as ‘Overall, how satisfied are you with your life nowadays?’, with responses indicated on a scale ranging from not at all to completely. Full details of the measures used in each study are available in online supplemental file 4.

In EHRs, mental health outcomes were considered as any morbidity code recorded in primary (GP, based on Clinical Terms Version 3 (CTV3) or Snomed codes) or secondary (hospital, based on Internaitonal Classification of Diseases Tenth version (ICD-10 codes)) care for diagnoses of depression, self-harm, anxiety, OCD, eating disorders or SMIs (schizophrenia, bipolar disorder or other psychoses) as defined in online supplemental table 1. Prescriptions issued to treat these conditions were not included as outcomes in the main analysis. Within each condition separately, a maximum of one event per individual per month was included.

### Analytical strategy

To answer objective 1 (estimate the mental health gap between people living alone and living with others), we compare the levels of distress and the rates of various mental health conditions in those living alone and those living with others.

To answer objective 2 (whether the gap changed during the pandemic), we use a multilevel modelling approach in the longitudinal studies with a time×living alone interaction to estimate whether there was a change in the difference during the pandemic. For EHRs, we used a quasi-experimental approach with an interrupted time series to estimate monthly period prevalence prior to and during the pandemic while accounting for seasonality, heteroskedasticity and autocorrelation.

The analysis steps in each data source are described below in greater detail.

#### Longitudinal population surveys

First, within each study, we estimated the association between living alone and our mental health outcomes at each time point cross-sectionally, using linear regression for continuous outcomes, and logistic regression for binary caseness outcomes. We tested for effect modification by whether the person was recommended to shield (identified as extremely clinically vulnerable to hospitalisation from COVID-19), loneliness, sex, age groups and prior mental health status (caseness at nearest pre-pandemic sweep).

To allow pooling and comparisons of effect sizes across studies, we standardised continuous psychological distress score across all time points within each cohort so estimates are interpretable on the same scale. We then estimated longitudinal multilevel models within each cohort, with a time×living alone (at T1) interaction term to test any changes in the gap during the pandemic.

Both cross-sectional and longitudinal models were estimated unadjusted, and then adjusted for the following covariates as available in each cohort: sex (male; female), age (coded in groups: 16–24, 25–34, 35–44, 45–54, 55–64, 65–74, 75+ years), education (degree vs no degree; parental education used for the Millennium Cohort Study), ethnicity (white, non-white), UK nation (England, Scotland, Wales or Northern Ireland), area (urban/rural), occupational class (manual, non-manual), home ownership (owned or mortgaged; other), disability (yes, no), prior chronic conditions/illness (yes, no). All LPS variables analysed in this study were retrospectively harmonised (ie, recoded) to ensure consistency across the studies. Full details of the data processing are documented in online supplemental file 5. Models were weighted to be representative of their target population, and account for sampling design and differential non-response, except for TwinsUK, Generation Scotland and ALSPAC, where no weights were available.

Results from each study were pooled using a random-effects meta-analysis with restricted maximum likelihood. Interaction coefficients between time and each of the modifiers were also meta-analysed to inform subsequent stratification. The I^2^ statistic, ratio of the between-study variability of the treatment effect to the sum of the between-study and within-study variability, was estimated as an indicator of heterogeneity between study estimates. In sensitivity analyses, random-effects meta-regression was conducted to investigate whether between-study heterogeneity could be explained by individual studies’ mental health measurement, time between the pre-pandemic and first pandemic measurement, and representativeness of their target population. All meta-analyses and meta-regressions were conducted in Stata V.17.

#### Electronic health records

We calculated the monthly period prevalence of each mental health outcome by dividing the number of people with the outcome by the total adult population (meeting inclusion criteria and currently registered with a GP) in that month. This was done each month between March 2018 and January 2022. The period prevalence was stratified by those living alone versus living with others. The change in period prevalence was estimated using a linear interrupted time series analysis with Newey-West robust SEs to account for heteroskedasticity. The interruption was defined to be a binary variable comparing pre-pandemic versus mid-pandemic periods (ie, after the start of the COVID-19 pandemic in England on 23 March 2020). We accounted for seasonal differences in period prevalence by including a categorical variable for spring (March, April, May), summer (June, July, August), autumn (September, October, November) and winter (December, January, February) with spring serving as our baseline. Lastly, we included a lag of 1 to account for autocorrelation between 2 consecutive months.

Subgroup differences in our results were tested using the same approach while introducing additional stratifying variables for age (using broad groups 18–39, 40–59, 60–79, 80+ years), sex, STP region, quintile of IMD, ethnicity (white, mixed, Asian, black, other, unknown), eligibility for shielding (defined as extremely clinically vulnerable to being hospitalised with COVID-19) and a binary indicator for history of mental health problems in the 5 years prior to February 2020. In sensitivity analysis, we performed analyses restricting our outcomes to: (1) those recorded in primary care and (2) those recorded in secondary care to explore whether there were differences between primary and secondary care due to the reduction in primary care services during the pandemic (see online supplemental table 1). For all variables in the analysis except ethnicity, recording is based on the presence or absence of codes and therefore there are no missing data. Data analysis was carried out in Stata V.17. Our code with full version control, disorder code lists and protocol are publicly available on GitHub (https://github.com/opensafely/lone_households).

## Results

Of the 37 544 participants in the LPS, 5716 lived alone (15.2%), and this figure was 21.4% for patients in the EHR (3 422 631 of 15 983 045). Descriptive statistics for each LPS are presented in table 1 and online supplemental table 2, and for EHRs, patient characteristics are given in table 2.

### Mental health gap between those who live alone and those who live with others

**Figure 1:**
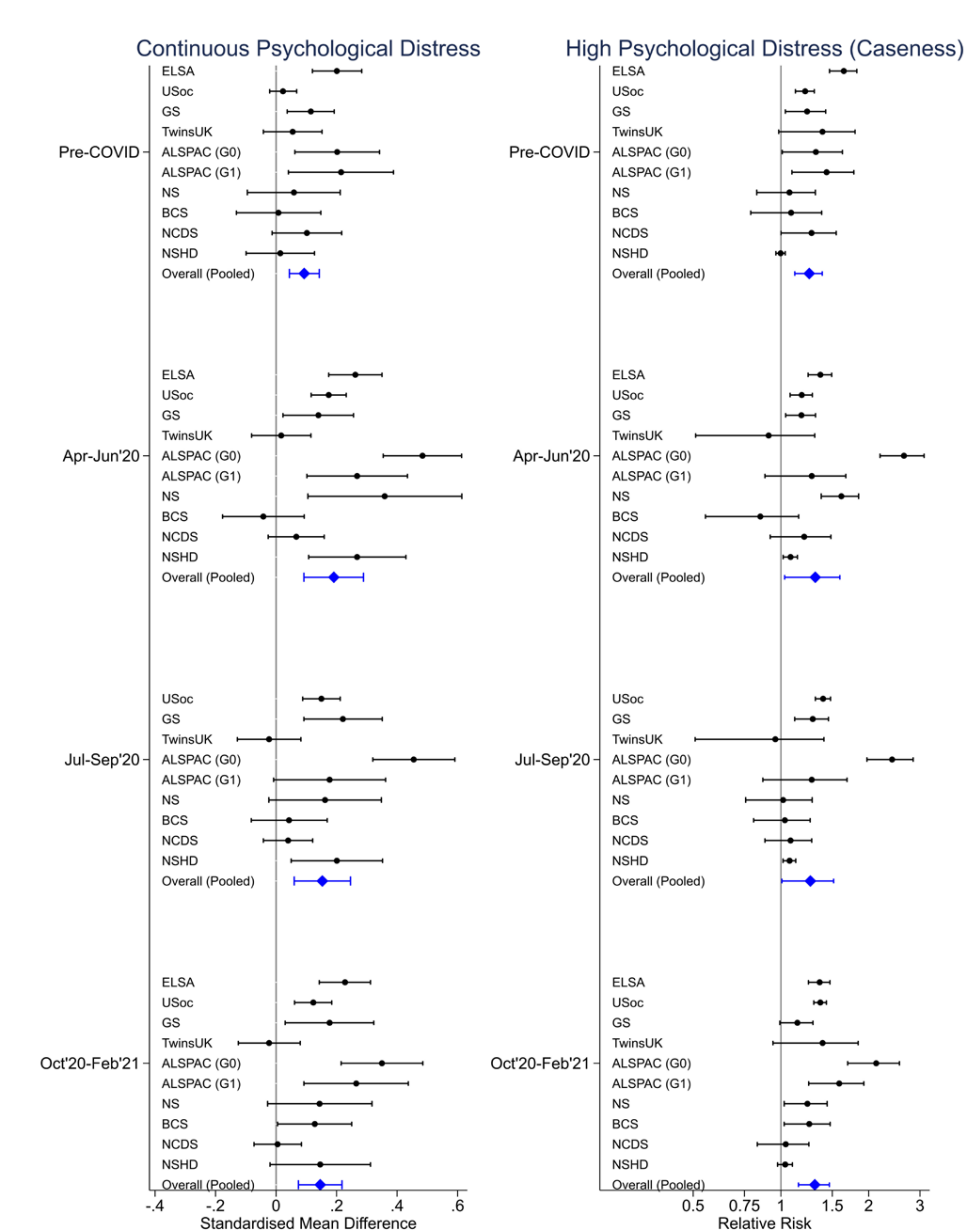
Regression estimates comparing those living alone with those living with others at each time point for each longitudinal study and the pooled estimate for the continuous standardised distress scores (left panel) and the binary score (right panel). ALSPAC (G0), Avon Longitudinal Study of Parents and Children-Generation 0 (Parents); ALSPAC (G1), Avon Longitudinal Study of Parents and Children-Generation 1; BCS, British Cohort Study; ELSA, English Longitudinal Study of Ageing; GS, Generation Scotland: the Scottish Family Health Study; NCDS, National Child Development Study; NS, Next Steps; NSHD, National Survey of Health and Development; TwinsUK, the UK Adult Twin Registry; USoc, Understanding Society: the UK Household Longitudinal Survey.

In the LPS, figure 1 shows modelling estimates for each time point for both continuous (on the scale of SDs) and binary coding (clinical cut-offs) of psychological distress (see online supplemental tables 3–8 for full details). At all time points and measures, symptoms were higher for those who lived alone. Before the pandemic, we observed greater mean psychological distress (standardised mean difference (SMD): 0.09 (95% CI: 0.04, 0.14)), higher risk of scoring above the cut-offs reflecting probable disorder (relative risk: 1.25 (95% CI: 1.12, 1.39)) and lower mean life satisfaction (SMD: −0.22 (95% CI: −0.30 to –0.15)) in those living alone. Full results of the meta-analyses are presented in online supplemental figure 1 and online supplemental table 9.

In EHRs, the pre-pandemic prevalence of mental health outcomes per 100 000 was higher among those living alone compared with those living with others. For example, depression (by 26 cases per 100 000 per month, 95% CI 18 to 33), anxiety (14 cases per 100 000 per month, 95% CI 6 to 21) and self-harm (26 cases per 100 000 per month, 95% CI 24 to 28) (online supplemental table 10). The difference in eating disorders was on average 3 cases more among those living alone per 100 000 (95% CI 2 to 3). There was a small average difference of −0.31 cases per 100 000 patients per month (95% CI 0 to 1) for OCD although statistically significant. For SMI, those living with others had approximately 58 fewer cases of SMI per 100 000 individuals per month than those living alone (95% CI 54 to 62) (online supplemental table 10).

### Effect of the pandemic on the mental health gap between those who live alone and those who live with others

As seen in figure 1, the mental health gaps between groups were similar in magnitude both before and during the pandemic. For the majority of LPSs, overall psychological distress and life satisfaction worsened over the course of the pandemic; however, there was not consistent evidence of a change in the gap between those living alone and with others (see online supplemental table 8 for time×living alone interactions).

**Figure 2:**
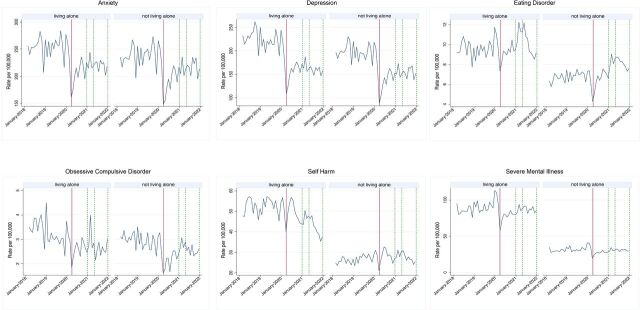
Period prevalence per 100 000 patients by mental health outcome from March 2018 to January 2022 in OpenSAFELY-TPP. Solid red lines indicate the introduction of the first lockdown in England in March 2020. Dotted green lines represent subsequent restrictive measures. All regression estimates can be found under ‘main effects’ in online supplemental file 3.

**Figure 3:**
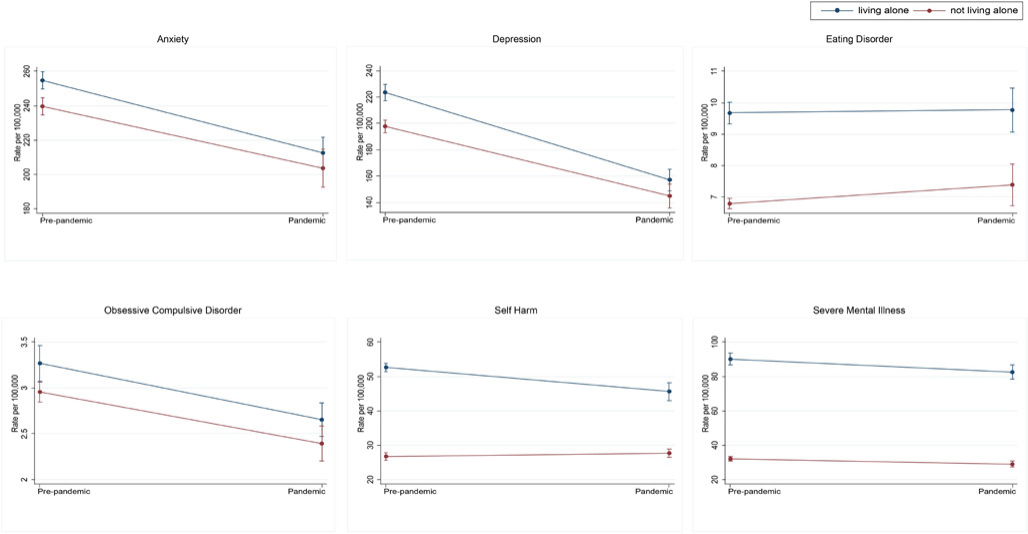
Interrupted time series marginal effects of pre-pandemic versus pandemic periods on outcome measures per 100 000 patients in OpenSAFELY-TPP. Blue lines represent people living alone while red lines are people living with others. Whiskers illustrate 95% CI.

In the EHRs (figures 2 and 3), records indicating anxiety and depression across people from all included households dropped during the pandemic, and there was no longer a difference in the gap in prevalence between those living alone and those living with others during the pandemic. For self-harm, while there was a reduction of 8 recorded cases per 100 000 (95% CI 5 to 11) individuals per month during the pandemic (robust SE=1.44, p<0.001), the difference between people living alone compared with living with others persisted (online supplemental table 10).

Contrary to anxiety, depression and self-harm, there was no evidence that the pandemic had an effect on monthly prevalence of eating disorder cases recorded in primary or secondary care (online supplemental table 10). The pre-pandemic difference observed between groups continued after the onset of the pandemic. Notably, the distributions of cases of eating disorder of those living alone versus living with others are dissimilar. While those living with others saw little variance in monthly prevalence prior to the pandemic, dispersion increased during the pandemic. Variation among those living alone was historically more pronounced but more closely resembled the variance of eating disorder in those living with others during pandemic periods.

Period prevalence of OCD presents a similar picture to eating disorders with lower variance in cases among those living with others prior to the pandemic, followed by a notable increase in dispersion during the pandemic. After the onset of the pandemic, there was no detectable difference in OCD between those living alone and those living with others. Furthermore, people from all household sizes saw a drop in recorded monthly OCD prevalence after the start of the pandemic. There was no noticeable pandemic effect on reported SMI among people living with others.

In the LPS, meta-analysis of study-specific interaction terms between each modifier of interest (age, sex, shielding status or reported levels of loneliness) and time period indicated that the gap in psychological distress and life satisfaction between those living alone and those living with others did not vary by any of these modifiers. Stratified estimates are displayed in online supplemental table 6.

In the EHRs, we did observe some differences between subgroups for some mental health outcomes (online supplemental tables 11–17). These must be interpreted with caution due to multiple testing and very large sample sizes, leading to small p values but potentially limited clinical significance.

### Sensitivity analyses

While individual estimates for both high psychological distress and life satisfaction varied between the LPSs, meta-regression analysis found that heterogeneity was somewhat explained by the type of mental health measure used, but could not be explained by time between pre-pandemic and during pandemic measures or the representativeness of the studies for their target population (online supplemental table 18). Leave-one-out meta-analysis found that no individual study significantly skewed the pooled estimates (online supplemental table 19).

## Discussion

Our study aimed to describe the disparity across a range of mental health outcomes between those who live alone versus with others, and whether the pandemic impacted on this disparity. Using data from longitudinal studies and EHRs, we found consistent evidence of poorer mental health in people who lived alone prior to the onset of the COVID-19 pandemic. Results from the longitudinal studies showed that the gap for psychological distress persisted through the pandemic. The EHR analyses for depression and anxiety, on the other hand, indicated a narrowing of the mental health gap in recorded cases after the beginning of the pandemic, highlighting the possibility that those living alone, although not having reduced need, showed greater reductions in healthcare-seeking and access.

Results from the LPS suggested that common mental health problems, such as symptoms of depression and anxiety, were greater in people who lived alone both prior to and during the pandemic. These symptoms increased for both groups during the pandemic; however, the size of the gap did not vary significantly across the pandemic. Indeed, living alone is unlikely to be a direct risk for mental illness—rather those who live alone may already be more vulnerable to poor mental health, receive less social support and experience higher levels of loneliness (which in turn may lead to greater mental ill-health).^5 24 25^ Therefore, the negative impact of living alone on mental health may be mitigated by strong social ties.^5 26^ A similar gap between lone and non-lone households was identified for life satisfaction, and this difference remained consistent prior to and during the pandemic.

The initial increase in common mental health symptoms during the pandemic did not translate to increased access to mental healthcare in people who lived alone. Our analyses of EHRs indicated that, while there was a small difference in service use between the two groups pre-pandemic for depression and anxiety, this gap narrowed during the pandemic as rates of diagnosis plunged for both people who lived alone and with others. The reduction in the gap suggests that there might have been more barriers to help-seeking for those who lived alone compared with those living with others during the pandemic (eg, in deciding to make or travel to GP appointments). The overall drop is consistent with other EHR studies that noted a sharp decline in primary care contacts across almost all physical and mental health conditions after the onset of the pandemic^27^ and is likely due to barriers to access that were an unintended consequence of the nationwide social restrictions introduced to combat the spread of the virus. It is noteworthy that the rates of diagnosis in EHRs had still not come back to pre-pandemic levels by 2022, although there being an increased need as indicated by higher distress levels in surveys.

Looking at lower-prevalence outcomes, results from the EHR analyses indicated that there were higher rates of eating disorders, self-harm, SMI and OCD in people who lived alone prior to the pandemic. While our analysis demonstrated that the pandemic was associated with an overall drop in the number of healthcare contacts for self-harm, SMI and OCD, the relative gaps between the two housing groups remained largely unchanged. However, eating disorders did not see a fall in records during the pandemic. The difference in patterns between the severe and the more common mental health conditions may have been for two reasons: first because severe conditions are more likely to result in healthcare contact, and second because their lower prevalence may have impacted our power to detect a difference. Lastly, EHR analysis revealed a notable increase in variance in rates of anxiety, depression and eating disorder, seemingly independent of a person’s living status. While period prevalence for these mental health outcomes illustrated high degree of precision prior to the COVID-19 outbreak, this is no longer the case during pandemic, indicating that some individuals continued to consult, while others likely avoided using healthcare. The finding has implications for future healthcare planning, impact and forecasting studies as estimates may be less reliable, and it is important to understand who was likely to avoid accessing healthcare and why.

The use of different data sources, with their different strengths and sources of bias, permits an examination of the levels of need and healthcare-seeking behaviours, highlighting contrasts in these during the pandemic. Evidence from both data sources demonstrates that those living alone experience greater distress and rates of all examined conditions. However, during the pandemic, there is some indication from the survey data that might have stayed the same or increased more in those living alone, but this did not translate to more healthcare-seeking behaviours in this group (as reflected in rates in EHRs), and instead for common disorders, the gap narrowed, suggesting potentially greater barriers to healthcare access for this group. Examples of such barriers during the pandemic might include practical barriers such as the unavailability of someone to accompany individuals to healthcare settings and psychological barriers such as greater anxiety related to leaving one’s residence to access healthcare.

This study focused on describing and delineating the differences between those living alone compared with others in a wide range of mental health outcomes and healthcare-seeking and diagnosis, rather than explaining the differences observed. Important explanatory considerations include understanding who is more likely to live alone and the health, disability, sociodemographic and psychological determinants of living alone. It is also possible that health and mental health are determinants of living alone,^28^ and this study did not aim to untangle temporality and causal direction in this relationship. In addition, many potential mechanisms are likely to play a role including social isolation, subjective and psychological feelings such as loneliness and perceived social support that further investigations should aim to better understand. In addition, although we do not observe effect modification by key demographic groupings such as age, sex and ethnicity in the LPS, it is possible that there are more complex intersectional effects at play (eg, older women, particular ethnic group at a certain age, etc); and future research might further investigate potential subgroups that might demonstrate differential effects.

Our study had a number of strengths. By drawing on two distinct forms of data (EHR and longitudinal surveys), we add robustness to our findings by balancing the strengths and weaknesses of each data source. Both had rich data before and during the pandemic. Most of the longitudinal cohorts included were weighted to be representative of their target population and accounted for sampling design and differential non-response. Furthermore, our harmonisation strategy across the 10 longitudinal cohorts allowed us to develop comparable exposures, outcomes and covariates, and pool estimates for similar time periods. However, despite this, the between-study heterogeneity of estimates was large, further highlighting the need to triangulate results from multiple sources, rather than relying on a single data source, when informing policy and health planning. Further benefits unique to our EHR analysis include the statistical power to study serious but low-prevalence mental illness and provide an indication of service use, which is important information for service planning.

However, the findings of our study should also be considered in light of the following limitations. The proportion of participants living alone overall in LPS (15%) was similar to national estimates (around 12%); however, in EHRs, the proportion (20%) was substantially higher than nationally, suggesting that individuals living alone are over-represented in healthcare settings, or reflecting potentially greater measurement error in this indicator in the EHR data. It is important to note that in both data sources, individuals who are homeless or have very insecure housing are less likely to be represented, and this is a highly vulnerable group in terms of their mental health.^29^ In the LPS, the timing of the assessments, both pre-COVID-19 and during the pandemic, was different between the longitudinal cohorts, although we tried to mitigate this by grouping assessments together into broad time frames that corresponded with key milestones in the pandemic (eg, first national lockdown, easing of restrictions, second national lockdown). The survey measures of psychological distress varied across the longitudinal cohorts, and although scores were standardised within each cohort for our analyses, the slightly different symptoms captured by different scales might contribute to heterogeneity in estimates. EHR-specific limitations are that records reflect healthcare use which, in turn, depends on several factors including the availability of healthcare, healthcare-seeking behaviour, and for mental health issues in particular, trust in the healthcare system. Differential access to services among people living alone compared with those who do not could also introduce ascertainment bias in either direction. We only included people who were registered (and therefore with household status recorded) on 1 February 2020, so we did not capture people who moved house during the pandemic, and who could have moved house because they lived alone or were experiencing a mental health problem. This was not possible to quantify in our EHR data sources. In the LPS, living status at T1 was used as the exposure in the longitudinal models, meaning changes in housing status were not accounted for. However, we conducted sensitivity analyses for the cross-sectional models in which living status later in the pandemic was used as exposure. In addition, we present descriptive statistics of living status across the four time points, with the proportion of participants living alone broadly consistent across time. Finally, the EHR analysis only had access to the coded GP and hospital records and only included diagnosed mental health conditions as outcomes, and some important symptoms of mental health conditions may be recorded as free text (EHRs also reflect clinician’s diagnostic beliefs and coding practices), leading to underascertainment. However, this GP coding behaviour is unlikely to be differential by household status, although as our findings suggest, there still might be important differences in healthcare-seeking behaviour by household status.

### Implications and conclusions

Our analyses offer some of the most robust evidence to date that people who live alone are at increased risk of both common and severe mental illness compared with people who live with others. However, we found little evidence to suggest that the medium-term mental health consequences of the pandemic were more keenly felt by those who live alone. Our findings highlight that accounting for household composition and lone households is an important consideration in allocation of services for mental health, especially given the much higher recording of SMIs in this group. As the number of people living alone continues to grow in the UK and other high-income countries, understanding the nuance between living alone, social isolation and loneliness, and their impact on mental health is increasingly important. Understanding the drivers and barriers to healthcare-seeking, especially as some of these might be different for those who live alone, will help inform practices to support this vulnerable group better.

### Data sharing

Details of data sharing for both the EHRs and LPSs used in this study are provided in the online supplemental materials.

### Software and reproducibility

For EHRs, data management and analysis were performed using the OpenSAFELY software libraries and Python, both implemented using Python V.3. Code for data management and analysis as well as code lists were archived online (https://github.com/opensafely/lone_households). All iterations of the prespecified study protocol are archived with version control. Further details are found in the online supplemental file 6.

For LPSs, datasheets and code are available online at: https://osf.io/wthpg/?view_only=a3a1b0837bea4e9ea1ace90079821a90.

## Data Availability

Data may be obtained from a third party and are not publicly available (see details in data sharing statement and in Supplementary file).
