# Supplementary material for: Living alone and mental health: parallel analyses in UK longitudinal population surveys and electronic health records prior to and during the COVID-19 pandemic
Source: BMJ Ment Health. 2023 Aug 10;26(1):e300842. doi: 10.1136/bmjment-2023-300842 (PMC10577768; doi:10.1136/bmjment-2023-300842)

Living alone and mental health: parallel analyses in longitudinal population surveys and electronic health records prior to and during the COVID-19 pandemic

Supplementary materials

Contents

Supplementary file 1. Ethics and data availability statements 3
Supplementary file 2. Funding statements for longitudinal population surveys 4
Supplementary file 3. Further study information - longitudinal population surveys (as required by the host institutions) 5
Supplementary file 4. Details of measures used in longitudinal population surveys 6
Supplementary file 5. Variable coding 8
Supplementary file 6. Further details on Open SAFELY 17
Table S1. Mental health outcomes used in EHR study 18
Table S2. Descriptive statistics for outcome measures in LPS by cohort, time and living status 19
Table S3. Regression coefficients from cross-sectional models with continuous outcomes 20
Table S4 Regression coefficients (non-exponentiated) from cross-sectional models with binary outcomes 25
Table S5. Interactions effects from cross-sectional models with continuous outcomes 30
Table S6. Regression coefficients from stratified cross-sectional models with continuous outcomes 43
Table S7. Regression coefficients from sensitivity analysis (excluding those living in households of larger than ten) 59
Table S8. Results from longitudinal multi-level models (time x lone household interactions) 62
Table S9. Results from the meta-analysis in LPS 64
Table S10. Main effects from EHR analyses 65
Table S11. Sex-stratified estimates from EHR analyses 65
Table S12. Age band-stratified estimates from EHR analyses 65
Table S13. Ethnicity-stratified estimates from EHR analyses 66
Table S14. IMD-stratified estimates from EHR analyses 66
Table S15. Region-stratified estimates from EHR analyses 67
Table S16. Shielded-stratified estimates from EHR analyses 67
Table S17. Previous mental illness-stratified estimates from EHR analyses 68

McElroy E, *et al. BMJ Ment Health* 2023; 26:e300842. doi: 10.1136/bmjment-2023-300842

Table S18. Heterogeneity between estimates of psychological distress explained by cohort differences (meta-regression analyses) 69
Table S19 Leave-One-Out Analysis 70
Figure S1. Results of meta-analyses in LPS 71

Supplementary file 1. Ethics and data availability statements

The most recent sweeps of the Millennium Cohort Study (MCS), Next Steps (NS), the British Cohort Study 1970 (BCS70), the National Child Development Study (NCDS) and the National Survey of Health and Development (NSHD) and have all been granted ethical approval by the National Health Service (NHS) Research Ethics Committee and all participants have given informed consent. Data for MCS (SN 8682), NS (SN 5545), BCS70 (SN 8547), NCDS (SN 6137) and all four COVID-19 surveys (SN 8658) are available through the UK Data Service. NSHD data are available on request to the NSHD Data Sharing Committee. Interested researchers can apply to access the NSHD data via a standard application procedure. Data requests should be submitted to mrclha.swiftinfo@ucl.ac.uk; further details can be found at http://www.nshd.mrc.ac.uk/data.aspx. doi:10.5522/NSHD/Q101; doi:10.5522/NSHD/Q10.

Ethical approval was obtained from the Avon Longitudinal Study of Parents and Children (ALSPAC) Ethics and Law Committee and the Local Research Ethics Committees. The study website contains details of all the data that is available through a fully searchable data dictionary and variable search tool: http://www.bristol.ac.uk/alspac/researchers/our-data. ALSPAC data is available to researchers through an online proposal system. Information regarding access can be found on the ALSPAC website (http://www.bristol.ac.uk/media-library/sites/alspac/documents/researchers/data-access/ALSPAC_Access_Policy.pdf).

The University of Essex Ethics Committee has approved all data collection for the Understanding Society (USoc) main study and COVID-19 waves. No additional ethical approval was necessary for this secondary data analysis. All data are available through the UK Data Service (SN 6614 and SN 8644).

Waves 1-9 of the English Longitudinal Study of Aging (ELSA) were approved through the National Research Ethics Service, while the COVID-19 Sub-study was approved by the UCL Research Ethics Committee. All participants provided informed consent. All data are available through the UK Data Service (SN 8688 and 5050).

Generation Scotland (GS) obtained ethical approval from the East of Scotland Committee on Medical Research Ethics (on behalf of the National Health Service). Reference number 20/ES/0021. Access to data is approved by the Generation Scotland Access Committee. See https://www.ed.ac.uk/generation-scotland/for-researchers/access or email access@generationscotland.org for further details.

All waves of TwinsUK (TwinsUK) have received ethical approval associated with TwinsUK Biobank (19/NW/0187), TwinsUK (EC04/015) or Healthy Ageing Twin Study (H.A.T.S) (07/H0802/84) studies from NHS Research Ethics Committees at the Department of Twin Research and Genetic Epidemiology, King's College London. The TwinsUK Resource Executive Committee (TREC) oversees management, data sharing and collaborations involving the TwinsUK registry (for further details see https://twinsuk.ac.uk/resources-for-researchers/access-our-data/).

Supplementary file 2. Funding statements for longitudinal population surveys

ALSPAC: The UK Medical Research Council and Wellcome (Grant Ref: 217065/Z/19/Z) and the University of Bristol provide core support for ALSPAC. A comprehensive list of grants funding is available on the ALSPAC website (http://www.bristol.ac.uk/alspac/external/documents/grant-acknowledgements.pdf). We are extremely grateful to all the families who took part in this study, the midwives for their help in recruiting them, and the whole ALSPAC team, which includes interviewers, computer and laboratory technicians, clerical workers, research scientists, volunteers, managers, receptionists and nurses. Part of this data was collected using REDCap, see the REDCap website for details: https://projectredcap.org/resources/citations/). This work was supported by Wellcome through the Wellcome Longitudinal Population Studies COVID-19 Secretariat and Steering Group (UK LPS COVID co-ordination, Grant Ref: 221574/Z/20/Z) and supported by the Elizabeth Blackwell Institute, University of Bristol, Wellcome Trust Institutional Strategic Support Fund and Rosetrees Trust (Grant Ref: 204813/Z/16/Z; R105121). ASFK is funded by an Economics and Social Research Council (ESRC) Postdoctoral Fellowship (ES/V011650/1).

USOC: Understanding Society is an initiative funded by the Economic and Social Research Council and various Government Departments, with scientific leadership by the Institute for Social and Economic Research, University of Essex, and survey delivery by NatCen Social Research and Kantar Public. The Understanding Society COVID-19 study is funded by the Economic and Social Research Council (ES/K005146/1) and the Health Foundation (2076161). The research data are distributed by the UK Data Service.

MCS, NS, BCS, NCDS, NSHD: The Millennium Cohort Study, Next Steps, 1970 British Cohort Study and 1958 National Child Development Study are supported by the Centre for Longitudinal Studies, Resource Centre 2015-20 grant (ES/M001660/1) and a host of other co-funders. The 1946 NSHD cohort is hosted by the the MRC Unit for Lifelong Health and Ageing funded by the Medical Research Council (MC_UU_00019/1Theme 1: Cohorts and Data Collection). The COVID-19 data collections in these five cohorts were funded by the UKRI grant Understanding the economic, social and health impacts of COVID-19 using lifetime data: evidence from 5 nationally representative UK cohorts (ES/V012789/1)

ELSA: The English Longitudinal Study of Ageing was developed by a team of researchers based at University College London, NatCen Social Research, the Institute for Fiscal Studies, the University of Manchester and the University of East Anglia. The data were collected by NatCen Social Research. The funding is currently provided by the National Institute on Aging in the US, and a consortium of UK government departments coordinated by the National Institute for Health Research. Funding has also been received by the Economic and Social Research Council. The English Longitudinal Study of Ageing Covid-19 Substudy was supported by the UK Economic and Social Research Grant (ESRC) ES/V003941/1.

GS: Generation Scotland received core support from the Chief Scientist Office of the Scottish Government Health Directorates [CZD/16/6] and the Scottish Funding Council [HR03006]. Genotyping of the GS:SFHS samples was carried out by the Genetics Core Laboratory at the Wellcome Trust Clinical Research Facility, Edinburgh, Scotland and was funded by the Medical Research Council UK and the Wellcome Trust (Wellcome Trust Strategic Award “STratifying Resilience and Depression Longitudinally” (STRADL)

Reference 104036/Z/14/Z). Generation Scotland is funded by the Wellcome Trust (216767/Z/19/Z).

TwinsUK: TwinsUK receives funding from the Wellcome Trust (WT212904/Z/18/Z), the National Institute for Health Research (NIHR) Biomedical Research Centre based at Guy's and St Thomas' NHS Foundation Trust and King's College London. The TwinsUK COVID-19 personal experience study was funded by the King's Together Rapid COVID-19 Call award, under the projects original title ‘Keeping together through coronavirus: The physical and mental health implications of self-isolation due to the Covid-19 TwinsUK is also supported by the Chronic Disease Research Foundation and Zoe Global Ltd. The funders had no role in study design, data collection and analysis, decision to publish, or preparation of the manuscript.

Supplementary file 3. Further study information - longitudinal population surveys (as required by the host institutions)

ALSPAC: Pregnant women resident in Avon, UK with expected dates of delivery 1st April 1991 to 31st December 1992 were invited to take part in the study. The initial number of pregnancies enrolled is 14,541 (for these at least one questionnaire has been returned or a “Children in Focus” clinic had been attended by 19/07/99). Of these initial pregnancies, there was a total of 14,676 foetuses, resulting in 14,062 live births and 13,988 children who were alive at 1 year of age. When the oldest children were approximately 7 years of age, an attempt was made to bolster the initial sample with eligible cases who had failed to join the study originally. As a result, when considering variables collected from the age of seven onwards (and potentially abstracted from obstetric notes) there are data available for more than the 14,541 pregnancies mentioned above. The number of new pregnancies not in the initial sample (known as Phase I enrolment) that are currently represented on the built files and reflecting enrolment status at the age of 24 is 913 (456, 262 and 195 recruited during Phases II, III and IV respectively), resulting in an additional 913 children being enrolled. The phases of enrolment are described in more detail in the cohort profile paper and its update (see footnote 4 below). The total sample size for analyses using any data collected after the age of seven is therefore 15,454 pregnancies, resulting in 15,589 foetuses. Of these 14,901 were alive at 1 year of age. A 10% sample of the ALSPAC cohort, known as the Children in Focus (CiF) group, attended clinics at the University of Bristol at various time intervals between 4 to 61 months of age. The CiF group were chosen at random from the last 6 months of ALSPAC births (1432 families attended at least one clinic). Excluded were those mothers who had moved out of the area or were lost to follow-up, and those partaking in another study of infant development in Avon.

Supplementary file 4. Details of measures used in longitudinal population surveys

MCS: The K-61 is a 6-item measure of psychological distress (i.e., general anxiety and depression). Responses are rated on a 5-point Likert-type scale, and capture distress over a period of four weeks prior to administration of the scale. Scores range from 0 to 24, with a conservative cut-off of 13+ applied to indicate probable psychological distress.

ALSPAC: Self-reported depressive symptoms were measured using the short mood and feelings questionnaire (SMFQ).5 The SMFQ is a 13-item questionnaire that measures the presence of depression symptoms in the previous two weeks and was administered via postal questionnaire or in research clinics. Each item is scored between 0-2, resulting in a summed score between 0-26. Scores of ≥11 on the SMFQ have good specificity and sensitivity for probable depression. In ALSPAC-G0, pre-pandemic depressive symptoms were measured using the Edinburgh Postnatal Depression Scale or EPDS, between 2011-2013. The EPDS consists of 10 items probing depressive symptoms in the last two weeks with scores ranging between 0-30 and higher scores indicating greater depression. Scores ≥ 12 have been validated against probable major depression. The SMFQ was used for pandemic measures of depressive symptoms in ALSPAC-G0.

NS: The 12-item General Health Questionnaire (GHQ)3 was used to detect symptoms of psychological distress in NS and USoc. The GHQ is a screening instrument designed to detect symptoms of psychological distress (i.e., general anxiety and depression). Each item is scored 0-3 resulting in scores ranging from 0-36. For classifying participants into high/low psychological distress, we used the common approach of scoring each item as 0-0-1-1, summing the results, and considering scores of 4+ as above the threshold for high psychological distress.

BCS70, NCDS: The 9-item version of the Malaise Inventory2 was used to assess general psychological distress. Items are scored using a simple ‘Yes/No’ response, meaning continuous scores range from 0-9. Scores of four or more are indicative of probable psychiatric distress.

NSHD: The General Health Questionnaire (GHQ)3 was also used to detect symptoms of psychological distress in NSHD. For the pre-pandemic sweep, the 28-item GHQ was used, though the 12-item GHQ was used for the subsequent 3 sweeps. A common set of 6 GHQ questions were identified and used in theses analyses. For continuous analyses, each item is scored 0-3 resulting in scores ranging from 0-18. For binary analyses, each item is scored as 0-0-1-1, resulting in scores ranging from 0-6 with a threshold of 2. Sensitivity analyses were conducted to confirm the validity of the 6-item GHQ.

USoc: The 12-item General Health Questionnaire (GHQ)3 was used, as described for NS.

ELSA: Depressive symptoms were measured using an abbreviated 8-item version of the validated Center for Epidemiologic Studies Depression Scale (CES-D).8 Respondents were asked whether they had experienced any depressive symptoms, such as feeling sad or having restless sleep, in the week prior to interview. For the binary classification, we considered respondents who reported four or more depressive symptoms on the CES-D scale as having elevated depressive symptoms.

TwinsUK: The Hospital Anxiety and Depression Scale (HADS)9 is a 14-item scale used to measure levels of psychiatric distress in non-psychiatric patient populations. Responses are indicated on a 4-point ordinal Likert scale.

GS: Depression and anxiety were assessed during the pandemic sweeps using the GAD-7 and the Patient Health Questionnaire (PHQ-9)4. The PHQ-9 is a nine-item, validated tool for the assessment of depressive symptoms experienced in the previous two weeks. Participants are asked to indicate how often they have been bothered by problems such as "Little interest or pleasure in doing things?" on a four-point Likert scale from 0 "Not at all" to 3 "Nearly every day". The score is the sum of the nine items, to a total of 27. A score of 10 or more indicates major depression. The 28-item GHQ3 was used to assess pre-pandemic psychological distress, and so a comparable composite measure was created from the GAD-7 and the PHQ-9 scales to enable evaluation of change over time. Correlations between each GHQ-28 item and items of the GAD-7 and PHQ-9 scales were examined, and the text of the most highly correlated (r > 0.25) questions was then reviewed and sense-checked; matched items are presented in table S1.2.T1 below. Cut-off scores were determined using ROC curves, which found a general cut-off of >4, a depression cut-off of >5 and an anxiety cut-off of >2. The latter matched the general "proportion" of PHQ-9/GAD-7 scores required for a cut-off.

**Pre-pandemic**				**Pandemic**			
**Measure**	**Subscale**	**Item**	**Question**	**Measure**	**Subscale**	**Item**	**Question**
GHQ-28	Somatic	3	Been feeling run down and out of sorts	PHQ-9	-	4	Feeling tired or having little energy
GHQ-28	Anxiety	2	Had difficulty in staying asleep once you are off	PHQ-9	-	3	Trouble falling or staying asleep, or sleeping too much
GHQ-28	Anxiety	4	Been getting edgy and bad-tempered	GAD-7	-	6	Becoming easily annoyed or irritable
GHQ-28	Anxiety	5	Been getting scared or panicky for no good reason	GAD-7	-	1	Feeling nervous, anxious or on edge
GHQ-28	Depression	1	Been thinking of yourself as a worthless person	PHQ-9	-	6	Feeling bad about yourself - or that you are a failure or have let yourself or your family down
GHQ-28	Depression	4	Thought of the possibility that you might make away with yourself	PHQ-9	-	9	Thoughts that you would be better off dead or of hurting yourself in some way
GHQ-28	Depression	5	Found at times you couldn't do anything because your nerves were too bad	PHQ-9	-	7	Trouble concentrating on things, such as reading the newspaper or watching television

Supplementary file 5. Variable coding

Variables	*Study*	*Question(s)*	*Recoding if needed*	*Notes*
*** Lone household * 0=Non-lone household; 1=Lives alone**				*** reference period**
	**NS<br>BCS 70<br>NCDS<br>NSHD**	How many people do you currently live with? Please include yourself.	1= 1 (lives alone); 0 = 2+ (non-lone household)	Reference period: T1 = Apr'20
	**USOC**	Thinking about the people who live in your household. Without counting yourself, how many are…? Aged 0-4; Aged 5-15; Aged 16-18; Aged 19-69; Aged 70+	0= 1 (lives alone)<br>1+ =0 (lives with others)	Used first valid data from either April, May or June Surveys.
	**ELSA**	[If living at usual home or at someone else's home] How many people (including you) are currently living in the residence you are staying in?	1= 1 (lives alone); 0 = 2+ (non-lone household)	Current situation in Jun/July 2020
	**GS**	1. Do you currently live alone? 2. Including yourself, how many people live in your household?	1. Yes = 1 (lives alone)<br>No = 0 (does not live alone)<br>Prefer not to answer = NA (excluded)<br>2. 1 - 12 plus (2-10 = 0; 11+ = excluded)	
	**TWINS UK**	Who do you live with?	o I live with other people 1==0<br>o I live by myself 2==1	
	**ALSPAC**	Number of people the participant lives with?	0 = Live alone; 1 = Live with at least one person	
*** Loneliness * 0=Not lonely; 1=Lonely**				
	**NS<br>BCS 70<br>NCDS<br>NSHD<br>USOC<br>ELSA**	How often do you feel lonely? 1= Hardly ever; 2= Some of the time; 3= Often	1+2=0; 3=1	

McElroy E, *et al. BMJ Ment Health* 2023; 26:e300842. doi: 10.1136/bmjment-2023-300842

	**GS**	How often have you felt lonely during the past week? 1 = None, or almost none of the time; 2 = Some of the time; 3 = Most of the time; 4 = All, or almost all of the time; 99 = Don't know; 98 = Prefer not to answer	1+2=0; 3+4=1, 98+99 = NA
	**TWINS UK**	How often do you feel like this NOW? How often do you feel alone? 1= Hardly ever; 2= Some of the time; 3= Often	1+2=0; 3=1
	**ALSPAC**	Not Available	
*** Sex * 0=Male; 1=Female**			
	**All**	0=Male; 1=Female	
*** Ethnicity * 0=White; 1=Ethnic Minority**		Note: Ethnicity is self-reported in these studies	
	**NS**	1=White; 2=Mixed; 3=Indian; 4=Pakistani; 5=Bangladeshi; 6=Black Caribbean; 7=Black African; 8=Other	1=0; 2/8=1
	**BCS70**	Not Available	
	**NCDS**	Not Available	
	**NSHD**	Not Available	
	**ALSPAC**	G0 (Parents) 1=White; 2=Black carribean; 3=Black african; 4=Other black; 5=Indian; 6=Pakistani; 7=Bangladeshi; 8=Chinese; 9=Other<br>G1 (Children) 1=White; 2=Mixed/Multiple Ethnic group; 3=Asian; 4=Black/African/Caribbean/Black British; 5=Arab or Other	1=0; 2/9=1
	**USOC**	1=White British; 2=Irish (White); 3=Gypsy or Irish Traveller (white); 4=Any other white background; 5=White and black caribbean (mixed); 6=White and black african (mixed); 7=White and Asian (mixed); 8=Any other mixed background; 9=Indian (Asian or Asian British); 10=Pakistani (Asian or Asian British); 11=Bangladeshi (Asian or Asian British); 12=Chinese (Asian or Asian British); 13=Any other Asian background (Asian or Asian British); 14=Caribbean (Black or Black British); 15=African (Black or Black British); 16=Any other Black background (Black or Black British); 17=Arab (other Ethnic group); 97=Any other ethnic group	1-4=0; 5-97=1
	**ELSA**	1.White; 2=Mixed ethnic group; 3=Black; 4=Black British; 5=Asian; 6=Asian British	1=0; 2/6=1

	**GS**	1=White Scottish; 2=White English; 3=White Welsh; 4=White N. Irish; 5=White Irish; 6=White Gypsy/Irish traveller; 7=White Polish; 8=Any other white; 9=Asian/British Asian - Indian; 10=Asian/British Asian - Pakistani; 11=Asian/British Asian - Bangladeshi; 12=Asian/British Asian - Chinese; 13=Any other Asian background; 14=Black or Black British - African; 15=Black or Black British - Carribean; 16=Any other Black/African/Caribbean background; 17=Arab or Arab British; 18=Mixed - White and Black Caribbean; 19=Mixed - White and Black African; 20=Mixed - White and Asian; 21=Any other Mixed/Multiple ethnic background; 22=Any other ethnic group	1-8=0; 9-22=1
	**TWINS UK**	1=White- English, Welsh, Scottish, Northern Irish, Irish; 2=White- Other white background; 3=Mixed/multiple ethnic groups - White and Black Caribbean; 4=Mixed/multiple ethnic groups - White and Black African; 5=Mixed/multiple ethnic groups - White and Asian; 6=Mixed/multiple ethnic groups - Other mixed/ multiple ethnic background; 7=Asian/Asian British- Indian; 8=Asian/Asian British - Pakistani; 9=Asian/Asian British - Bangladeshi; 10=Asian/Asian British - Chinese; 11=Asian/Asian British - Other Asian background; 12=Black/Black British - African; 13=Black/Black British - Caribbean; 14=Black/Black British - Other Black Background; 15=Middle-Eastern; 16=Other ethnic group	1/2=0; 3/16=1
		Note: Ethnicity is self-reported in these studies	
*** Education * 0=No Degree; 1=Degree**			
	**NS BCS 70 NCDS**	0=None; 1=Nvq1; 2=Nvq2; 3=Nvq3; 4=Nvq4; 5=Nvq5 *parent's education for MCS	1/3=0; 4/5=1
	**NSHD**	0=None attempted; 1.=Vocational course, proficiency only; 2=Sub GCE or sub Burnham C; 3=GCE 'O' level or Burnham C; 4=GCE 'A' Level or Burnham B; 5=Burnham A2; 6= 1st Degree or graduate equivalent; 7= Higher degree, Masters; 8= Higher degree, doctorate; 9=Unknown	0/5 OR 9=0; 6/8=1
	**ALSPAC**	1=Degree; 2=A levels/AS levels or equivalent; 3=O levels; 4=Vocational; 5=CSE *parent's education for G1 (Children)	1=1; 2/5=0

**USOC**	1.Higher degree 2. 1st degree or equivalent 3. Diploma in Higher Education 4. Teaching qualification (not PGCE) 5. Nursing or other medical qualification 6. Other higher degree 7. A-Level 8. Welsh baccalaureate 9. International baccalaureate 10. AS Level 11. Scottish Highers 12. Certificate of 6th year studies 13. GCSE/O-Level 14. Certificate of secondary education 15. Standard or lower 16. Other school certificate 96. No qualifications	1/6=1; 7/96=0
**ELSA**	1=Nvq4/nvq5/degree or equivalent; 2=Higher Education below degree; 3=Nvq3/GCE A level equivalent; 4=Nvq2/GCE O level equivalent; 5=Nvq1/CSE other grade equivalent; 6=Foreign/other; 7=No qualification	1=1; 2/7=0
**GS**	1=No qualifications; 2=Other (please specify); 3=School leavers certificate; 4=CSEs or equivalent; 5=Standard grade, National 4 or 5, O levels, GCSEs or equivalent; 6=Higher grade, A levels, AS levels or equivalent; 7=NVQ or HND or HNC or equivalent; 8=Other professional or technical qualification; 9=Undergraduate degree; 10=Postgraduate degree	1/8=0; 9 OR 10=1
**TWINS UK**	1=No qualification; 2=NVQ1/SVQ1; 3=O-level/GCSE/NVQ2/SVQ2/Scottish intermediate; 4=Scottish Higher, NVQ3, City and Guilds, Pitman; 5=A-level, Scottish Advanced Higher; 6=Higher vocational training (e.g. Diploma, NVQ4, SVQ4); 7=Undergraduate degree; 8=Postgraduate degree (e.g. Masters or PhD), NVQ5, SVQ5	1/6=0; 7 OR 8=1

*** Occupational Social Class * 1=Managerial/Admin/Professional; 2=Intermediate; 3=Manual/routine; 4=Other/Missing**

NS-SEC: National Statistics Socioeconomic Classification.
RGSC: Registrat General's Social Class
ONS SOC: Office of National Statistics Standard Occupational Classification

	**NS BCS 70 NCDS**	[NS-SEC] 1=Higher managerial and professional; 2=Lower managerial and rofessional; 3=Intermediate occupations; 4=Small employers and own account workers; 5=Lower supervisory and technical; 6=Semi-routine occupations; 7=Routine occupations; 8=Never worked and long-term unemployed *parent's occupational social class for MCS	2=1; 3-4=2; 5-7=3; 8=4
	**NSHD**	[RGSC] 1=I Professional; 2=II Managerial and Technical; 3=IIINM Skilled non-manual; 4=IIIM Skilled manual; 5=IV Partly skilled; 6=V Unskilled; 7=Missing	2=1; 3/5=2; 6=3; 7=4
	**ALSPAC**	[RGSC] 1=I Professional; 2=II Managerial and Technical; 3=IIINM Skilled non-manual; 4=IIIM Skilled manual; 5=IV Partly skilled; 6=V Unskilled; 7=Armed Forces *parent's occupational social class for G1 (Children)	2=1; 3/5=2; 6=3; 7=4
	**USOC**	[NS-SEC] 1=Higher managerial and professional; 2=Lower managerial and rofessional; 3=Intermediate occupations; 4=Small employers and own account workers; 5=Lower supervisory and technical; 6=Semi-routine occupations; 7=Routine occupations; 8=Never worked or long-term unemployed	2=1; 3-4=2; 5-7=3; 8=4
	**ELSA**	[NS-SEC] 1=Higher managerial and professional; 2=Lower managerial and rofessional; 3=Intermediate occupations; 4=Small employers and own account workers; 5=Lower supervisory and technical; 6=Semi-routine occupations; 7=Routine occupations; 8=Never worked or long-term unemployed	2=1; 3-4=2; 5-7=3; 8=4
	**GS**	[ONS SOC] 1=Managers, directors, senior officials; 2=Associate professional and technical occupations; 3=Administrative and secretarial occupations; 4=Skilled trades occupations; 5=Sales and customer service occupations; 6=Process, plant and machine operatives; 7=Elementary (unskilled) occupations; 8=Never worked	1/3=1; 4/5=2; 6/7=3; 8=4
	**TWINS UK**	Not Available	
*** Shielding Status * 1=Shielding; 0=Not shielding**			
	**All studies except ALSPAC and MCS (given their age)**		

McElroy E, *et al. BMJ Ment Health* 2023; 26:e300842. doi: 10.1136/bmjment-2023-300842

	**NS<br>BCS 70<br>NCDS<br>NSHD**	[SHIELD] Did you at any time receive a letter or text message from the NHS or Chief Medical Officer saying that you have been identified as someone at risk of severe illness if you catch Coronavirus, because you have an underlying disease or health condition 1. Yes 2. No	2=0
	**USOC**	Have you received a letter from the NHS or Chief Medical Officer saying that you have been identified as someone at risk of severe illness if you catch coronavirus, because you have an underlying disease or health condition? 1=Yes, 2=No	2=0
	**ELSA**	“Have you been contacted by the NHS or your GP and advised that you are vulnerable and at risk of severe illness if you catch coronavirus (Covid-19), and should stay at home at all times and avoid any face-to-face contact?” 1=Yes, 2=No	2=0
	**GS**	Have you been contacted by letter or text message to say you are<br>at severe risk from COVID-19 due to an underlying health condition and should be shielding? 1 = Yes; 2= No.	2=0
	**TWINS UK**	Wave 2 (July/August 2020) Have you received a letter or text message over the past few months to say you are at high risk from COVID-19 due to an underlying health condition, and should be ‘shielding’? 1 = Yes; 2= No<br>Wave 4 (March/April 2021): Were you contacted by letter or text message to say you are at severe risk from COVID-19 due to an underlying health condition and should be shielding? 1 = Yes; 2= No	
	**ALSPAC**	Not Available	
*** Home ownership * 1=Home Owner; 0=Not Home Owner**			
	**NS<br>BCS 70<br>NCDS<br>NSHD**	TENURE | Do you (or your household) own or rent your home or have some other arrangement? 1. Own - outright 2. Own - buying with help of a mortgage / loan 3. Pay part rent and part mortgage (shared / equity ownership) 4. Rent it 5. Live here rent-free, including rent-free in relative's / friend's / employer’s property; exclude squatting 6. Squatting 7. Other arrangement	1/2=1; 3/7=0

	**USOC**	Derived from a series of questions: 1=Owned outright; 2=Owned with mortgage; 3=Local authority rent; 4=Housing association rent; 5=Rented from employer; 6=Rented private unfurnished; 7=Rented private furnished; 8=Other	1/2=1; 3/8=0
	**ELSA**	In which of these ways do you occupy this accommodation? 1=Own it outright; 2=Buying it with the help of a mortgage or loan; 3=Pay part rent and part mortgage shared ownership; 4=Rent it; 5=Live here rent free (including rent free in relative's / friend's property; excluding squatting); 6=Squatting	1/3=1; 4/6=0
	**ALSPAC**	Not Available	
	**GS**	What is the status of the accommodation in which you and your household live? 1=Own outright; 2=Own with mortgage; 3=Rent from local authority/housing association; 4=Rent from private landlord or agency; 5=Pay part rent and part mortgage;6=Live rent free; 7=Other; 8=Don't know; 9=Prefer not to answer	1/2=1; 3/9=0
	**TWINS UK**	Which describes the home you live in? Owned outright (1); Owned with the help of a mortgage (2); Shared ownership (part owned, part rented) (3); Rented (4); Living rent free (5); Other (Please specify below) (6)	1:3 = home owner; 4, 5 = non-home owner
*** Pre-Pandemic Physical Chronic Condition/Illness ***			
	**NCDS**	Whether CM has physical/mental health conditions/illnesses expected to last 12+mths. 1=Yes; 2=No	2=0
	**BCS70**	Any physical/mental health conditions lasting or expected to last 12 months. 1=Yes; 2=No	2=0
	**NS**	Has longstanding illness. 1=Yes; 2=No	2=0
	**NSHD**	CM suffering from a longstanding illness. 1=Yes; 2=No	2=0
	**USOC**	Do you have any long-standing physical or mental impairment, illness or disability? By 'long-standing' I mean anything that has troubled you over a period of at least 12 months or that is likely to trouble you over a period of at least 12 months. 1=Yes, 2=No<br><br>***I also have a variable derived from a list of physical chronic coniditions, let me know if you'd rather I used that***	2=0

McElroy E, *et al. BMJ Ment Health* 2023; 26:e300842. doi: 10.1136/bmjment-2023-300842

	**ELSA**	Q1: Do you have any long-standing illness, disability or infirmity? By long-standing I mean anything that has troubled you over a period of time, or that is likely to affect you over a period of time. 1=Yes; 2=No<br>Q2: (If Yes in Q1) (Does this / Do these) illness(es) or disability(ies) limit your activities in any way? 1=Yes; 2=No	If Q1=1 & Q2=1
	**ALSPAC**	Participant has a history of diabetes, heart disease, hypertension, is overweight, kidney disease, liver disease, anaemia, other lung conditions, cancer, condition affecting the brain and nerves or weakened immune system	0 = None; 1 = at least 1
	**GS**	Not Available	
	**TWINS UK**	Not Available	
*** Pre-Pandemic Disability * 1=If respondent disabled/has disability that limits their activity/work; 0=No Disabilities**			
	**All studies except GS and ALSPAC**		
	**NCDS BCS70 NS**	(Derived) Disability classification Equality act (2010). 0 = Not disabled; 1 = Disabled	
	**NSHD**	Do you have any long-term illness, health problem or disability that limits the activities/work you can do?	0 = No; 1 = Yes
	**USOC**	How much difficulty do you have with [separate items for: Mobility; Lifting, carrying or moving objects; Manual dexterity; Continence; Hearing; Sight; Physical Coordination]? Would you say… 1=Some difficulty; 2=A lot of difficulty; 3=Unable to do this	2/3 on any item=1; else=0
	**ELSA**	Please tell me if you have any difficulty with these everyday activities because of a physical, mental, emotional or memory problem. Exclude any difficulties you expect to last less than three months. 1-15=List of activities; 96=None of the everyday activities listed	1 if !=96
	**ALSPAC**	Not Available	
	**GS**	Not Available	

**TWINS UK**	Do you currently have a long-term disability that seriously restricts your activities? 0 = no; 1 = yes	
*** Urban/rural * 1=rural; 0=urban**		
**Prioritising T1**		
**NCDS BCS70 NS**	Not Available	
**NSHD**	Not Available	
**USOC**	Binary indicator classifying the address as falling into an (1) urban or (2) rural area. This is derived from the Office for National Statistics Rural and Urban Classification of Output Areas 2001 (UKDS Study Number 7454). The indicator assumes a value of (1) if the address falls within urban settlements with a population of 10,000 or more, or (2) otherwise.	
**ELSA**	Binary indicator classifying the address as falling into an (1) urban or (2) rural area. This is derived from the Office for National Statistics Rural and Urban Classification of Output Areas 2011. The indicator assumes a value of (1) if the address falls within urban settlements with a population of 10,000 or more, or (2) otherwise.	2=0
**ALSPAC**	Not Available	
**GS**	Based on the Scottish Government Urban-Rural Classification 2016. 1= urban; 2 = rural	2=0
**TWINS UK**	Binary indicator classifying the address as falling into an (1) urban or (2) rural area. This is derived from the Office for National Statistics Rural and Urban Classification of Output Areas 2011. The indicator assumes a value of (1) if the address falls within urban settlements with a population of 10,000 or more, or (2) otherwise.	

Supplementary file 6. Further details on Open SAFELY

DATA SHARING

All data were linked, stored and analysed securely within the OpenSAFELY platform https://opensafely.org/. Data include pseudonymized data such as coded diagnoses, medications and physiological parameters. No free text data are included. All code is shared openly for review and re-use under MIT open license ([https://github.com/opensafely/lone_households]). Detailed pseudonymised patient data is potentially re-identifiable and therefore not shared. We rapidly delivered the OpenSAFELY data analysis platform without prior funding to deliver timely analyses on urgent research questions in the context of the global Covid-19 health emergency: now that the platform is established we are developing a formal process for external users to request access in collaboration with NHS England; details of this process are available at OpenSAFELY.org.

SOFTWARE AND REPRODUCIBILITY

Data management and analysis was performed using the OpenSAFELY software libraries and Python, both implemented using Python 3. This analysis was delivered using federated analysis through the OpenSAFELY platform: codelists and code for data management and data analysis were specified once using the OpenSAFELY tools; then transmitted securely to the OpenSAFELY-TPP platform within TPP's secure environment, and separately to the OpenSAFELY-EMIS platform within EMIS's secure environment, where they were each executed separately against local patient data; summary results were then reviewed for disclosiveness, released, and combined for the final outputs. All code for the OpenSAFELY platform for data management, analysis and secure code execution is shared for review and re-use under open licenses at GitHub.com/OpenSAFELY. Data management was performed using Python 3.8, with analysis carried out using Stata 17. Code for data management and analysis as well as codelists archived online [https://github.com/opensafely/lone_households]. All iterations of the pre-specified study protocol are archived with version control.

ETHICS STATEMENT

We received ethics approval to conduct the data linkage and analyses by the London - City & East Research Ethics Committee on the 2nd of April 2020 (REC reference: 20/LO/0651) and LSHTM Ethics Board (ref 21863). No further ethical or research governance approval was required by the University of Oxford but copies of the approval documents were reviewed and held on record.

INFORMATION GOVERNANCE

NHS England is the data controller; TPP is the data processor; and the key researchers on OpenSAFELY are acting on behalf of NHS England. This implementation of OpenSAFELY is hosted within the TPP environment, which is accredited to the ISO 27001 information security standard and is NHS IG Toolkit compliant 52,53; patient data have been pseudonymized for analysis and linkage using industry standard cryptographic hashing techniques; all pseudonymized datasets transmitted for linkage onto OpenSAFELY are encrypted; access to the platform is through a virtual private network (VPN) connection; the researchers hold contracts with NHS England and only access the platform to initiate database queries and statistical models; all database activity is logged; and only aggregate statistical outputs leave the platform environment following best practice for anonymization of results such as statistical disclosure control for low cell counts54. The OpenSAFELY research platform adheres to the data protection principles of the UK Data Protection Act

2018 and the EU General Data Protection Regulation (GDPR) 2016. In March 2020, the Secretary of State for Health and Social Care used powers under the UK Health Service (Control of Patient Information) Regulations 2002 (COPI) to require organizations to process confidential patient information for the purposes of protecting public health, providing healthcare services to the public and monitoring and managing the COVID-19 outbreak and incidents of exposure55. Together, these provide the legal bases to link patient datasets on the OpenSAFELY platform. GP practices, from which the primary care data are obtained, are required to share relevant health information to support the public health response to the pandemic and have been informed of the OpenSAFELY analytics platform.

Table S1. Mental health outcomes used in EHR study

Outcome	Definition	Source
Depression	Any record of major depressive disorder, dysthymia, mixed anxiety and depression, and adjustment disorders with depressed mood; we will also include codes for depressive symptoms	Primary care
Self harm/suicide	Records that indicate explicit or undetermined intention to self-harm, non-suicidal or suicidal self-harm (including overdoses with drugs commonly implicated in suicide, such as paracetamol)	Primary care, hospital admission, emergency care attendance, death records
Anxiety	Any record of symptoms or diagnoses of social phobia, agoraphobia, panic, generalised anxiety disorder, and mixed anxiety and depression	Primary care
Obsessive compulsive disorder	Codes for hypochondriasis, hoarding disorder, and body focused repetitive behaviour disorders	Primary care
Eating disorders	Anorexia nervosa, bulimia nervosa, body dysmorphic disorders, and other specified feeding and eating disorders	Primary care, hospital admission, emergency care attendance, death records
Serious mental illness	Diagnoses of schizophrenia and other psychotic disorders, and bipolar disorders	Primary care, hospital admission, emergency care attendance

Table S2. Descriptive statistics for outcome measures in LPS by cohort, time and living status

			Pre-Pandemic			*Peri-Pandemic 1*			*Peri-Pandemic 2*			*Peri-Pandemic 3*		
**Longitudinal Study**	**Mental Health Outcome**	**Living Alone Status**	**N**	**Mean Score (SD)**	**High Symptoms (%)**	**N**	**Mean Score (SD)**	**High Symptoms (%)**	**N**	**Mean Score (SD)**	**High Symptoms (%)**	**N**	**Mean Score (SD)**	**High Symptoms (%)**
**NS**	Psychological Distress	*Living Alone*	249	11.6 (6.2)	25.9	156	14.4 (6.6)	47.8	144	14.1 (5.8)	32.3	161	14.0 (5.9)	41.3
		Living With Others	1013	11.4 (5.5)	23.9	1104	12.8 (5.5)	30.9	1038	13.0 (5.4)	30.6	1087	13.5 (5.4)	34.2
	Low Life Satisfaction	*Living Alone*	249	2.2 (0.8)	23.9	161	6.4 (2.2)	40.1	145	6.8 (1.8)	33.7	160	6.4 (1.9)	42.1
		Living With Others	1013	2.0 (0.8)	19.8	1128	7.2 (1.9)	28.9	1042	7.2 (1.8)	26.4	1093	6.6 (2.1)	39.9
**BCS**	Psychological Distress	*Living Alone*	229	1.7 (2.0)	20.2	377	1.7 (1.9)	15.8	347	2.0 (2.3)	21.2	339	2.0 (2.2)	22.6
		Living With Others	2564	1.5 (1.9)	14.9	2558	1.7 (2.0)	16.5	2326	1.9 (2.0)	19.2	2386	1.7 (1.9)	17.4
	Low Life Satisfaction	*Living Alone*	229	7.6 (1.8)	35.2	382	6.8 (2.0)	17.0	348	6.6 (2.1)	39.2	341	6.5 (2.1)	42.4
		Living With Others	2559	8.7 (1.6)	16.5	2578	7.3 (1.9)	25.0	2330	7.3 (1.8)	24.4	2390	6.9 (1.9)	33.5
**NCDS**	Psychological Distress	*Living Alone*	369	1.5 (1.8)	14.7	841	1.4 (1.8)	12.9	815	1.5 (1.9)	14.5	829	1.4 (1.8)	13.5
		Living With Others	3403	1.8 (1.7)	10.4	2936	1.3 (1.8)	9.1	2878	1.3 (1.8)	11.9	2895	1.3 (1.7)	11.4
	Low Life Satisfaction	*Living Alone*	369	6.6 1.8)	39.5	860	6.9 (2.2)	32.7	815	6.9 (2.0)	34.0	834	6.6 (2.2)	41.7
		Living With Others	3403	7.7 (1.5)	16.4	2974	7.6 (1.9)	20.6	2882	7.6 (1.8)	20.9	2898	7.1 (1.9)	32.1
**NSHD**	Psychological Distress	*Living Alone*	304	4.4 (1.8)	20.7	353	6.8 (2.5)	69.1	357	6.0 (2.2)	55.5	251	5.5 (2.1)	43.0
		Living With Others	1127	4.2 (1.8)	16.2	1266	6.2 (2.2)	62.2	1170	5.6 (2.1)	44.5	862	5.0 (1.9)	32.1
	Low Life Satisfaction	*Living Alone*	264	7.7 (2.2)	15.9	252	6.9 (2.2)	23.8	363	6.9 (2.0)	25.9	251	6.4 (2.0)	32.7
		Living With Others	998	7.7 (1.8)	12.2	859	7.7 (1.8)	12.2	1170	7.5 (1.9)	14.0	863	7.2 (1.9)	19.2
**ALSPAC-G1**	Psychological Distress	*Living Alone*	141	8.2 (6.4)	33.3	161	7.9 (6.5)	24.2	127	7.6 (6.0)	27.6	145	8.2 (7.0)	31.7
		Living With Others	2111	6.7 (6.2)	22.6	2632	6.1 (5.0)	17.7	1946	6.4 (5.7)	21.1	2323	6.4 (6.0)	20.7
	Low Life Satisfaction	*Living Alone*	-	-	-	-	-	-	-	-	-	-	-	-
		Living With Others	-	-	-	-	-	-	-	-	-	-	-	-
**USOC**	Psychological Distress	*Living Alone*	3328	11.7 (5.4)	20.7	1148	13.7 (6.4)	31.8	1089	13.3 (6.3)	26.7	1066	13.6 (6.3)	28.2
		Living With Others	18859	11.3 (5.6)	19.5	8508	12.4 (6.2)	24.6	7645	11.8 (5.7)	20.0	7670	12.5 (6.2)	24.3
	Low Life Satisfaction	*Living Alone*	3328	5.0 (1.4)	30.5	1190	4.4 (1.4)	49.1	1093	4.5 (1.4)	45.4	1067	4.5 (1.4)	43.7
		Living With Others	18859	5.1 (1.4)	26.9	8942	4.8 (1.6)	35.2	7661	4.9 (1.5)	32.0	7964	4.9 (1.5)	31.2
**ELSA**	Psychological Distress	*Living Alone*	1348	1.8 (2.3)	4.1	1451	2.6 (2.5)	7.8	-	-	-	1303	2.9 (2.7)	9.3
		Living With Others	4123	1.8 (1.6)	7.2	4020	1.9 (2.0)	14.4	-	-	-	3742	2.1 (2.3)	18.1
	Low Life Satisfaction	*Living Alone*	1214	6.9 (2.6)	7.6	1447	6.7 (2.5)	10.2	-	-	-	1300	6.5 (2.5)	10.9
		Living With Others	3876	7.6 (2.0)	18.1	4004	7.1 (2.1)	24.4	-	-	-	3737	7.0 (2.2)	26.2
**GS**	Psychological Distress	*Living Alone*	452	3.0 (3.0)	21.9	433	4.1 (4.5)	24.9	334	4.1 (4.2)	32.9	304	4.9 (4.6)	40.8
		Living With Others	2530	2.5 (2.4)	16.9	2447	3.6 (3.8)	30.6	1771	3.2 (3.8)	26.0	1628	4.2 (4.1)	35.1
	Low Life Satisfaction	*Living Alone*	452	7.7 (1.8)	17.7	452	5.8 (2.3)	57.8	347	6.6 (2.2)	38.2	324	5.7 (2.3)	56.5
		Living With Others	2528	8.2 (1.6)	11.1	2528	6.5 (2.2)	42.7	1836	7.2 (1.9)	28.5	1700	6.3 (2.2)	47.3
**TWINSUK**	Psychological Distress	*Living Alone*	480	7.5 (5.9)	7.1	480	8.5 (6.5)	6.5	480	9.76 (5.3)	5.2	480	10 (5.7)	7.1
		Living With Others	1847	7.2 (5.9)	5.7	1847	8.6 (6.5)	8.1	1847	10 (5.5)	6	1847	10.4 (5.7)	6.7
	Low Life Satisfaction	*Living Alone*	-	-	-	480	6.8 (2.2)	36.7	480	7.0 (2.03)	30	480	7.2 (2.0)	25.8
		Living With Others	-	-	-	1847	7.0 (2.0)	34.2	1847	7.4 (1.9)	22.4	1847	7.5 (1.9)	23.8
**ALSPAC-G0**	Psychological Distress	*Living Alone*	205	7.4 (6.4)	23.4	237	4.9 (4.9)	11.4	213	5.0 (5.1)	12.7	219	4.7 (5.0)	12.3
		Living With Others	2568	6.3 (5.2)	17.3	3047	2.8 (3.5)	3.8	2614	3.0 (3.7)	4.9	2667	3.2 (3.9)	5.8
	Low Life Satisfaction	*Living Alone*	-	-	-	-	-	-	-	-	-	-	-	-
		Living With Others	-	-	-	-	-	-	-	-	-	-	-	-

Table S3. Regression coefficients from cross-sectional models with continuous outcomes

cohort	outcome (Z)	TP	exposure	coef	se	l_ci	u_ci	n	Adjusted for
**NCDS**	Psych distress	T0	T0 living alone	0.17	0.06	0.06	0.29	3772	--
**NCDS**	Psych distress	T0	T0 living alone	0.10	0.06	-0.01	0.22	3551	sex, education, UK nation, occupation, housing tenure, disability, chronic illness
**NCDS**	Psych distress	T1	T1 living alone	0.17	0.06	0.06	0.28	3777	--
**NCDS**	Psych distress	T1	T1 living alone	0.07	0.05	-0.03	0.16	3567	sex, education, UK nation, occupation, housing tenure, disability, chronic illness
**NCDS**	Psych distress	T2	T1 living alone	0.11	0.04	0.02	0.19	3693	--
**NCDS**	Psych distress	T2	T1 living alone	0.04	0.04	-0.04	0.12	3692	sex, education, UK nation, occupation, housing tenure, disability, chronic illness
**NCDS**	Psych distress	T3	T1 living alone	0.07	0.04	-0.01	0.16	3724	--
**NCDS**	Psych distress	T3	T1 living alone	0.01	0.04	-0.07	0.08	3512	sex, education, UK nation, occupation, housing tenure, disability, chronic illness
**NCDS**	Life satisfaction	T0	T0 living alone	-0.56	0.05	-0.66	-0.45	3772	--
**NCDS**	Life satisfaction	T0	T0 living alone	-0.50	0.05	-0.61	-0.39	3551	sex, education, UK nation, occupation, housing tenure, disability, chronic illness
**NCDS**	Life satisfaction	T1	T1 living alone	-0.37	0.07	-0.50	-0.24	3834	--
**NCDS**	Life satisfaction	T1	T1 living alone	-0.32	0.06	-0.43	-0.21	3623	sex, education, UK nation, occupation, housing tenure, disability, chronic illness
**NCDS**	Life satisfaction	T2	T1 living alone	-0.35	0.04	-0.43	-0.26	3697	--
**NCDS**	Life satisfaction	T2	T1 living alone	-0.31	0.04	-0.39	-0.22	3696	sex, education, UK nation, occupation, housing tenure, disability, chronic illness
**NCDS**	Life satisfaction	T3	T1 living alone	-0.29	0.05	-0.38	-0.19	3732	--
**NCDS**	Life satisfaction	T3	T1 living alone	-0.26	0.05	-0.35	-0.17	3520	sex, education, UK nation, occupation, housing tenure, disability, chronic illness
**NSHD**	Psych distress	T0	T0 living alone	0.08	0.06	-0.03	0.19	1431	--
**NSHD**	Psych distress	T0	T0 living alone	0.01	0.06	-0.10	0.13	1300	sex, education, UK nation, tenure, disability
**NSHD**	Psych distress	T1	T1 living alone	0.36	0.08	0.21	0.51	1618	--
**NSHD**	Psych distress	T1	T1 living alone	0.27	0.08	0.11	0.43	1439	sex, education, UK nation, tenure, disability
**NSHD**	Psych distress	T2	T1 living alone	0.32	0.07	0.18	0.47	1526	--
**NSHD**	Psych distress	T2	T1 living alone	0.20	0.08	0.05	0.35	1445	sex, education, UK nation, tenure, disability
**NSHD**	Psych distress	T3	T1 living alone	0.23	0.08	0.08	0.39	1112	--
**NSHD**	Psych distress	T3	T1 living alone	0.15	0.08	-0.02	0.31	1053	sex, education, UK nation, tenure, disability
**GS**	Psych distress	T0	Live Alone	0.12	0.04	0.06	0.19	2984	--
**GS**	Psych distress	T1	Live Alone	0.13	0.06	0.02	0.24	2882	--
**GS**	Psych distress	T2	Live Alone	0.23	0.07	0.11	0.35	2106	--
**GS**	Psych distress	T3	Live Alone	0.22	0.08	0.08	0.36	1932	--
**GS**	Life satisfaction	T0	Live Alone	--	--	--	--	--	*No T0 LifeSat score

**GS**	Life satisfaction	T1	Live Alone	-0.33	0.05	-0.44	-0.23	2982	--
**GS**	Life satisfaction	T2	Live Alone	-0.26	0.06	-0.36	-0.15	2184	--
**GS**	Life satisfaction	T3	Live Alone	-0.28	0.06	-0.40	-0.16	2024	--
**GS**	Psych distress	T0	Live Alone	0.11	0.04	0.05	0.18	2984	sex, age, education, ethnicity, tenure, occupation, urbanicity
**GS**	Psych distress	T1	Live Alone	0.14	0.06	0.03	0.25	2882	sex, age, education, ethnicity, tenure, occupation, urbanicity
**GS**	Psych distress	T2	Live Alone	0.22	0.07	0.10	0.34	2106	sex, age, education, ethnicity, tenure, occupation, urbanicity
**GS**	Psych distress	T3	Live Alone	0.18	0.07	0.04	0.31	1932	sex, age, education, ethnicity, tenure, occupation, urbanicity
**GS**	Life satisfaction	T0	Live Alone	--	--	--	--	--	*No T0 LifeSat score
**GS**	Life satisfaction	T1	Live Alone	-0.33	0.05	-0.43	-0.23	2982	sex, age, education, ethnicity, tenure, occupation, urbanicity
**GS**	Life satisfaction	T2	Live Alone	-0.27	0.06	-0.37	-0.16	2184	sex, age, education, ethnicity, tenure, occupation, urbanicity
**GS**	Life satisfaction	T3	Live Alone	-0.25	0.06	-0.37	-0.13	2024	sex, age, education, ethnicity, tenure, occupation, urbanicity
**USOC**	Psych distress	T0	T0 living alone	0.05	0.02	0.01	0.10	22187	--
**USOC**	Psych distress	T0	T0 living alone	0.02	0.02	-0.02	0.07	22187	sex, age, education, ethnicity, UK nation, rural, social class, tenure, disability, illness
**USOC**	Psych distress	T1	T1 living alone	0.15	0.03	0.09	0.21	12088	--
**USOC**	Psych distress	T1	T1 living alone	0.17	0.03	0.12	0.23	12088	sex, age, education, ethnicity, UK nation, rural, social class, tenure, disability, illness
**USOC**	Psych distress	T2	T1 living alone	0.15	0.03	0.09	0.22	9905	--
**USOC**	Psych distress	T2	T1 living alone	0.15	0.03	0.09	0.21	9905	sex, age, education, ethnicity, UK nation, rural, social class, tenure, disability, illness
**USOC**	Psych distress	T3	T1 living alone	0.12	0.03	0.06	0.18	9913	--
**USOC**	Psych distress	T3	T1 living alone	0.12	0.03	0.06	0.18	9913	sex, age, education, ethnicity, UK nation, rural, social class, tenure, disability, illness
**USOC**	Life satisfaction	T0	T0 living alone	-0.09	0.02	-0.14	-0.05	22187	--
**USOC**	Life satisfaction	T0	T0 living alone	-0.06	0.02	-0.11	-0.02	22187	sex, age, education, ethnicity, UK nation, rural, social class, tenure, disability, illness
**USOC**	Life satisfaction	T1	T1 living alone	-0.29	0.04	-0.37	-0.21	10132	--
**USOC**	Life satisfaction	T1	T1 living alone	-0.26	0.04	-0.35	-0.18	10132	sex, age, education, ethnicity, UK nation, rural, social class, tenure, disability, illness
**USOC**	Life satisfaction	T2	T1 living alone	-0.21	0.03	-0.27	-0.15	9905	--
**USOC**	Life satisfaction	T2	T1 living alone	-0.17	0.03	-0.22	-0.11	9905	sex, age, education, ethnicity, UK nation, rural, social class, tenure, disability, illness
**USOC**	Life satisfaction	T3	T1 living alone	-0.24	0.03	-0.29	-0.18	9913	--
**USOC**	Life satisfaction	T3	T1 living alone	-0.20	0.03	-0.25	-0.14	9913	sex, age, education, ethnicity, UK nation, rural, social class, tenure, disability, illness
**BCS**	Psych distress	T0	T0 living alone	0.08	0.08	-0.08	0.24	2793	--
**BCS**	Psych distress	T0	T0 living alone	0.01	0.07	-0.13	0.15	2461	sex, education, UK nation, occupation, housing tenure, disability, chronic illness
**BCS**	Psych distress	T1	T1 living alone	0.02	0.07	-0.12	0.15	2935	--
**BCS**	Psych distress	T1	T1 living alone	-0.04	0.07	-0.18	0.09	2616	sex, education, UK nation, occupation, housing tenure, disability, chronic illness
**BCS**	Psych distress	T2	T1 living alone	0.09	0.07	-0.04	0.22	2673	--

**BCS**	Psych distress	T2	T1 living alone	0.04	0.06	-0.08	0.17	2673	sex, education, UK nation, occupation, housing tenure, disability, chronic illness
**BCS**	Psych distress	T3	T1 living alone	0.16	0.07	0.02	0.30	2725	--
**BCS**	Psych distress	T3	T1 living alone	0.13	0.06	0.01	0.25	2406	sex, education, UK nation, occupation, housing tenure, disability, chronic illness
**BCS**	Life satisfaction	T0	T0 living alone	-0.49	0.07	-0.64	-0.35	2788	--
**BCS**	Life satisfaction	T0	T0 living alone	-0.43	0.07	-0.56	-0.30	2457	sex, education, UK nation, occupation, housing tenure, disability, chronic illness
**BCS**	Life satisfaction	T1	T1 living alone	-0.24	0.08	-0.40	-0.08	2960	--
**BCS**	Life satisfaction	T1	T1 living alone	-0.17	0.09	-0.33	0.00	2638	sex, education, UK nation, occupation, housing tenure, disability, chronic illness
**BCS**	Life satisfaction	T2	T1 living alone	-0.39	0.06	-0.51	-0.26	2678	--
**BCS**	Life satisfaction	T2	T1 living alone	-0.32	0.06	-0.44	-0.21	2678	sex, education, UK nation, occupation, housing tenure, disability, chronic illness
**BCS**	Life satisfaction	T3	T1 living alone	-0.24	0.07	-0.38	-0.10	2731	--
**BCS**	Life satisfaction	T3	T1 living alone	-0.19	0.06	-0.31	-0.06	2412	sex, education, UK nation, occupation, housing tenure, disability, chronic illness
**Next Steps**	Psych distress	T0	T0 living alone	0.03	0.09	-0.15	0.20	1262	--
**Next Steps**	Psych distress	T0	T0 living alone	0.06	0.08	-0.10	0.21	1114	sex, ethnicity, education, UK nation, occupation, housing tenure, disability, chronic illness
**Next Steps**	Psych distress	T1	T1 living alone	0.28	0.16	-0.04	0.59	1260	--
**Next Steps**	Psych distress	T1	T1 living alone	0.36	0.13	0.10	0.61	1135	sex, ethnicity, education, UK nation, occupation, housing tenure, disability, chronic illness
**Next Steps**	Psych distress	T2	T1 living alone	0.19	0.10	-0.01	0.38	1182	--
**Next Steps**	Psych distress	T2	T1 living alone	0.16	0.09	-0.02	0.35	1182	sex, ethnicity, education, UK nation, occupation, housing tenure, disability, chronic illness
**Next Steps**	Psych distress	T3	T1 living alone	0.10	0.10	-0.10	0.29	1248	--
**Next Steps**	Psych distress	T3	T1 living alone	0.14	0.09	-0.03	0.32	1103	sex, ethnicity, education, UK nation, occupation, housing tenure, disability, chronic illness
**Next Steps**	Life satisfaction	T0	T0 living alone	0.05	0.02	0.01	0.09	1262	--
**Next Steps**	Life satisfaction	T0	T0 living alone	0.04	0.02	0.00	0.09	1114	sex, ethnicity, education, UK nation, occupation, housing tenure, disability, chronic illness
**Next Steps**	Life satisfaction	T1	T1 living alone	-0.27	0.11	-0.48	-0.06	1289	--
**Next Steps**	Life satisfaction	T1	T1 living alone	-0.25	0.11	-0.47	-0.04	1158	sex, ethnicity, education, UK nation, occupation, housing tenure, disability, chronic illness
**Next Steps**	Life satisfaction	T2	T1 living alone	-0.14	0.06	-0.27	-0.02	1187	--
**Next Steps**	Life satisfaction	T2	T1 living alone	-0.12	0.06	-0.24	0.00	1187	sex, ethnicity, education, UK nation, occupation, housing tenure, disability, chronic illness
**Next Steps**	Life satisfaction	T3	T1 living alone	-0.06	0.07	-0.20	0.07	1253	--
**Next Steps**	Life satisfaction	T3	T1 living alone	-0.05	0.06	-0.17	0.07	1106	sex, ethnicity, education, UK nation, occupation, housing tenure, disability, chronic illness
**ELSA**	Psych distress	T0	T0 living alone	0.35	0.05	0.26	0.44	5471	--
**ELSA**	Psych distress	T0	T0 living alone	0.20	0.04	0.12	0.28	5471	Sex ; Age; Education; Ethnicity, Occupational class; Home ownership; Disability; chronic il
**ELSA**	Psych distress	T1	T1 living alone	0.37	0.05	0.28	0.47	5470	--
**ELSA**	Psych distress	T1	T1 living alone	0.26	0.04	0.17	0.35	5470	Sex ; Age; Education; Ethnicity, Occupational class; Home ownership; Disability; chronic il
**ELSA**	Psych distress	T3	T1 living alone	0.34	0.05	0.25	0.44	5046	--

McElroy E, *et al. BMJ Ment Health* 2023; 26:e300842. doi: 10.1136/bmjment-2023-300842

**ELSA**	Psych distress	T3	T1 living alone	0.23	0.04	0.14	0.31	5046	Sex ; Age; Education; Ethnicity, Occupational class; Home ownership; Disability; chronic il
**ELSA**	Life Satisfaction	T0	T0 living alone	-0.38	0.04	-0.46	-0.29	5090	--
**ELSA**	Life Satisfaction	T0	T0 living alone	-0.33	0.04	-0.41	-0.24	5090	Sex ; Age; Education; Ethnicity, Occupational class; Home ownership; Disability; chronic il
**ELSA**	Life Satisfaction	T1	T1 living alone	-0.16	0.04	-0.25	-0.08	5453	--
**ELSA**	Life Satisfaction	T1	T1 living alone	-0.12	0.04	-0.21	-0.04	5453	Sex ; Age; Education; Ethnicity, Occupational class; Home ownership; Disability; chronic il
**ELSA**	Life Satisfaction	T3	T1 living alone	-0.23	0.05	-0.32	-0.14	5038	--
**ELSA**	Life Satisfaction	T3	T1 living alone	-0.21	0.05	-0.30	-0.12	5038	Sex ; Age; Education; Ethnicity, Occupational class; Home ownership; Disability; chronic il
**TwinsUK**	Psych distress	T0	Live Alone	0.05	0.05	-0.05	0.15	2327	--
**TwinsUK**	Psych distress	T1	Live Alone	-0.03	0.05	-0.13	0.07	2323	--
**TwinsUK**	Psych distress	T2	Live Alone	-0.05	0.06	-0.16	0.06	2020	--
**TwinsUK**	Psych distress	T3	Live Alone	-0.07	0.05	-0.17	0.04	2141	--
**TwinsUK**	Life satisfaction	T0	Live Alone	--	--	--	--		
**TwinsUK**	Life satisfaction	T1	Live Alone	-0.08	0.05	-0.19	0.02	2327	--
**TwinsUK**	Life satisfaction	T2	Live Alone	-0.19	0.05	-0.30	-0.08	2026	--
**TwinsUK**	Life satisfaction	T3	Live Alone	-0.12	0.05	-0.22	-0.02	2155	--
**TwinsUK**	Psych distress	T0	Live Alone	0.06	0.05	-0.04	0.15	2327	sex, age, ethnicity, edu, country, ruc, homeownership, disability, srh_pp_bin
**TwinsUK**	Psych distress	T1	Live Alone	0.02	0.05	-0.08	0.11	2323	sex, age, ethnicity, edu, country, ruc, homeownership, disability, srh_pp_bin
**TwinsUK**	Psych distress	T2	Live Alone	-0.02	0.05	-0.13	0.08	2020	sex, age, ethnicity, edu, country, ruc, homeownership, disability, srh_pp_bin
**TwinsUK**	Psych distress	T3	Live Alone	-0.02	0.05	-0.13	0.08	2141	sex, age, ethnicity, edu, country, ruc, homeownership, disability, srh_pp_bin
**TwinsUK**	Life satisfaction	T0	Live Alone	-	-	-	-	-	
**TwinsUK**	Life satisfaction	T1	Live Alone	-0.12	0.05	-0.22	-0.02	2327	sex, age, ethnicity, edu, country, ruc, homeownership, disability, srh_pp_bin
**TwinsUK**	Life satisfaction	T2	Live Alone	-0.15	0.05	-0.26	-0.05	2026	sex, age, ethnicity, edu, country, ruc, homeownership, disability, srh_pp_bin
**TwinsUK**	Life satisfaction	T3	Live Alone	-0.13	0.05	-0.24	-0.03	2155	sex, age, ethnicity, edu, country, ruc, homeownership, disability, srh_pp_bin
**ALSPAC-G1**	Psych distress	T0	Live Alone	0.23	0.08	0.07	0.40	2252	--
**ALSPAC-G1**	Psych distress	T1	Live Alone	0.31	0.08	0.15	0.47	2793	--
**ALSPAC-G1**	Psych distress	T2	Live Alone	0.21	0.09	0.03	0.39	2073	--
**ALSPAC-G1**	Psych distress	T3	Live Alone	0.28	0.08	0.12	0.45	2468	--
**ALSPAC-G1**	Life satisfaction	T0	Live Alone	-0.29	0.09	-0.46	-0.12	2178	--
**ALSPAC-G1**	Life satisfaction	T1	Live Alone	-0.34	0.08	-0.50	-0.18	2813	--

**ALSPAC-G1**	Life satisfaction	T2	Live Alone	-0.16	0.09	-0.34	0.01	2080	--
**ALSPAC-G1**	Life satisfaction	T3	Live Alone	-0.26	0.08	-0.43	-0.10	2474	--
**ALSPAC-G1**	Psych distress	T0	Live Alone	0.21	0.09	0.04	0.39	1809	sex, ethnicity, occupational class, education, chronic illness
**ALSPAC-G1**	Psych distress	T1	Live Alone	0.27	0.08	0.10	0.43	2064	sex, ethnicity, occupational class, education, chronic illness
**ALSPAC-G1**	Psych distress	T2	Live Alone	0.18	0.09	-0.01	0.36	1610	sex, ethnicity, occupational class, education, chronic illness
**ALSPAC-G1**	Psych distress	T3	Live Alone	0.26	0.09	0.09	0.44	1919	sex, ethnicity, occupational class, education, chronic illness
**ALSPAC-G1**	Life satisfaction	T0	Live Alone	-0.27	0.09	-0.45	-0.09	1720	sex, ethnicity, occupational class, education, chronic illness
**ALSPAC-G1**	Life satisfaction	T1	Live Alone	-0.34	0.09	-0.51	-0.17	2075	sex, ethnicity, occupational class, education, chronic illness
**ALSPAC-G1**	Life satisfaction	T2	Live Alone	-0.16	0.10	-0.35	0.03	1616	sex, ethnicity, occupational class, education, chronic illness
**ALSPAC-G1**	Life satisfaction	T3	Live Alone	-0.29	0.09	-0.47	-0.12	1920	sex, ethnicity, occupational class, education, chronic illness
**ALSPAC-G0**	Psych distress	T0	Live Alone	0.23	0.07	0.09	0.37	2773	--
**ALSPAC-G0**	Psych distress	T1	Live Alone	0.56	0.07	0.43	0.69	3284	--
**ALSPAC-G0**	Psych distress	T2	Live Alone	0.51	0.07	0.37	0.65	2827	--
**ALSPAC-G0**	Psych distress	T3	Live Alone	0.38	0.07	0.24	0.51	2886	--
**ALSPAC-G0**	Psych distress	T0	Live alone	0.20	0.07	0.06	0.34	2702	sex, age, ethnicity, occupational class, education, chronic illness
**ALSPAC-G0**	Psych distress	T1	Live alone	0.48	0.07	0.35	0.61	3177	sex, age, ethnicity, occupational class, education, chronic illness
**ALSPAC-G0**	Psych distress	T2	Live alone	0.46	0.07	0.32	0.59	2746	sex, age, ethnicity, occupational class, education, chronic illness
**ALSPAC-G0**	Psych distress	T3	Live alone	0.35	0.07	0.22	0.48	2808	sex, age, ethnicity, occupational class, education, chronic illness

Note: TP = timepoint; coef = coefficient; se = standard error ; l_ci = lower 95% confidence interval ; u_ci = cupper 95% confidence interval

Table S4 Regression coefficients (non-exponentiated) from cross-sectional models with binary outcomes

cohort	outcome	TP	exposure	coef	se	l_ci	u_ci	n	Adjusted for
**NCDS**	Psych distress	T0	T0 living alone	0.35	0.14	0.08	0.62	3772	--
**NCDS**	Psych distress	T0	T0 living alone	0.24	0.14	-0.03	0.52	3551	sex, education, UK nation, occupation, housing tenure, disability, chronic illness
**NCDS**	Psych distress	T1	T1 living alone	0.35	0.14	0.08	0.63	3777	--
**NCDS**	Psych distress	T1	T1 living alone	0.18	0.14	-0.10	0.47	3567	sex, education, UK nation, occupation, housing tenure, disability, chronic illness
**NCDS**	Psych distress	T2	T1 living alone	0.20	0.10	0.00	0.40	3693	--
**NCDS**	Psych distress	T2	T1 living alone	0.08	0.10	-0.12	0.27	3692	sex, education, UK nation, occupation, housing tenure, disability, chronic illness
**NCDS**	Psych distress	T3	T1 living alone	0.16	0.11	-0.04	0.37	3724	--
**NCDS**	Psych distress	T3	T1 living alone	0.04	0.11	-0.17	0.25	3512	sex, education, UK nation, occupation, housing tenure, disability, chronic illness
**NCDS**	Life satisfaction	T0	T0 living alone	-0.32	0.04	-0.41	-0.24	3772	--
**NCDS**	Life satisfaction	T0	T0 living alone	-0.29	0.04	-0.38	-0.21	3551	sex, education, UK nation, occupation, housing tenure, disability, chronic illness
**NCDS**	Life satisfaction	T1	T1 living alone	-0.17	0.03	-0.23	-0.10	3834	--
**NCDS**	Life satisfaction	T1	T1 living alone	-0.13	0.03	-0.20	-0.07	3623	sex, education, UK nation, occupation, housing tenure, disability, chronic illness
**NCDS**	Life satisfaction	T2	T1 living alone	-0.18	0.03	-0.23	-0.13	3697	--
**NCDS**	Life satisfaction	T2	T1 living alone	-0.16	0.03	-0.21	-0.10	3696	sex, education, UK nation, occupation, housing tenure, disability, chronic illness
**NCDS**	Life satisfaction	T3	T1 living alone	-0.15	0.03	-0.22	-0.09	3732	--
**NCDS**	Life satisfaction	T3	T1 living alone	-0.16	0.03	-0.22	-0.09	3520	sex, education, UK nation, occupation, housing tenure, disability, chronic illness
**BCS**	Psych distress	T0	T0 living alone	0.30	0.17	-0.04	0.64	2793	--
**BCS**	Psych distress	T0	T0 living alone	0.08	0.15	-0.22	0.38	2461	sex, education, UK nation, occupation, housing tenure, disability, chronic illness
**BCS**	Psych distress	T1	T1 living alone	-0.04	0.18	-0.40	0.32	2935	--
**BCS**	Psych distress	T1	T1 living alone	-0.16	0.15	-0.46	0.14	2616	sex, education, UK nation, occupation, housing tenure, disability, chronic illness
**BCS**	Psych distress	T2	T1 living alone	0.10	0.12	-0.13	0.32	2673	--
**BCS**	Psych distress	T2	T1 living alone	0.03	0.12	-0.19	0.26	2673	sex, education, UK nation, occupation, housing tenure, disability, chronic illness
**BCS**	Psych distress	T3	T1 living alone	0.26	0.13	0.01	0.51	2725	--
**BCS**	Psych distress	T3	T1 living alone	0.22	0.11	0.00	0.45	2406	sex, education, UK nation, occupation, housing tenure, disability, chronic illness
**BCS**	Life satisfaction	T0	T0 living alone	-0.25	0.06	-0.37	-0.14	2788	--
**BCS**	Life satisfaction	T0	T0 living alone	-0.20	0.05	-0.29	-0.10	2457	sex, education, UK nation, occupation, housing tenure, disability, chronic illness
**BCS**	Life satisfaction	T1	T1 living alone	-0.17	0.07	-0.31	-0.04	2960	--
**BCS**	Life satisfaction	T1	T1 living alone	-0.10	0.06	-0.21	0.01	2638	sex, education, UK nation, occupation, housing tenure, disability, chronic illness
**BCS**	Life satisfaction	T2	T1 living alone	-0.22	0.05	-0.31	-0.13	2678	--

**BCS**	Life satisfaction	T2	T1 living alone	-0.19	0.05	-0.27	-0.10	2678	sex, education, UK nation, occupation, housing tenure, disability, chronic illness
**BCS**	Life satisfaction	T3	T1 living alone	-0.14	0.05	-0.25	-0.04	2731	--
**BCS**	Life satisfaction	T3	T1 living alone	-0.04	0.05	-0.14	0.05	2412	sex, education, UK nation, occupation, housing tenure, disability, chronic illness
**NS**	Psych distress	T0	T0 living alone	0.08	0.13	-0.17	0.33	1262	--
**NS**	Psych distress	T0	T0 living alone	0.07	0.12	-0.18	0.31	1114	sex, ethnicity, education, UK nation, occupation, housing tenure, disability, chronic ill
**NS**	Psych distress	T1	T1 living alone	0.44	0.15	0.14	0.73	1260	--
**NS**	Psych distress	T1	T1 living alone	0.48	0.12	0.24	0.71	1135	sex, ethnicity, education, UK nation, occupation, housing tenure, disability, chronic ill
**NS**	Psych distress	T2	T1 living alone	0.05	0.13	-0.21	0.32	1182	--
**NS**	Psych distress	T2	T1 living alone	0.02	0.13	-0.24	0.28	1182	sex, ethnicity, education, UK nation, occupation, housing tenure, disability, chronic ill
**NS**	Psych distress	T3	T1 living alone	0.19	0.12	-0.04	0.42	1248	--
**NS**	Psych distress	T3	T1 living alone	0.21	0.11	0.00	0.42	1103	sex, ethnicity, education, UK nation, occupation, housing tenure, disability, chronic ill
**NS**	Life satisfaction	T0	T0 living alone	-0.05	0.04	-0.13	0.03	1262	--
**NS**	Life satisfaction	T0	T0 living alone	-0.07	0.04	-0.15	0.01	1114	sex, education, UK nation, occupation, housing tenure, disability, chronic illness
**NS**	Life satisfaction	T1	T1 living alone	-0.17	0.10	-0.37	0.02	1289	--
**NS**	Life satisfaction	T1	T1 living alone	-0.15	0.11	-0.38	0.07	1158	sex, education, UK nation, occupation, housing tenure, disability, chronic illness
**NS**	Life satisfaction	T2	T1 living alone	-0.11	0.07	-0.24	0.03	1187	--
**NS**	Life satisfaction	T2	T1 living alone	-0.09	0.07	-0.22	0.04	1187	sex, education, UK nation, occupation, housing tenure, disability, chronic illness
**NS**	Life satisfaction	T3	T1 living alone	-0.04	0.08	-0.20	0.12	1253	--
**NS**	Life satisfaction	T3	T1 living alone	-0.08	0.09	-0.25	0.10	1106	sex, education, UK nation, occupation, housing tenure, disability, chronic illness
**NSHD**	Psych distress	T0	T0 living alone	0.02	0.02	-0.01	0.06	1431	**--**
**NSHD**	Psych distress	T0	T0 living alone	0.00	0.02	-0.04	0.03	1300	sex, education, UK nation, tenure, disability
**NSHD**	Psych distress	T1	T1 living alone	0.10	0.03	0.05	0.16	1618	**--**
**NSHD**	Psych distress	T1	T1 living alone	0.08	0.03	0.02	0.14	1439	sex, education, UK nation, tenure, disability
**NSHD**	Psych distress	T2	T1 living alone	0.10	0.03	0.05	0.16	1526	**--**
**NSHD**	Psych distress	T2	T1 living alone	0.07	0.03	0.01	0.12	1445	sex, education, UK nation, tenure, disability
**NSHD**	Psych distress	T3	T1 living alone	0.06	0.03	0.00	0.12	1112	**--**
**NSHD**	Psych distress	T3	T1 living alone	0.03	0.03	-0.03	0.09	1053	sex, education, UK nation, tenure, disability
**GS**	Psych distress	T0	Live Alone	0.26	0.10	0.03	0.47	2984	--
**GS**	Psych distress	T1	Live Alone	0.13	0.07	-0.05	0.30	2882	--
**GS**	Psych distress	T2	Live Alone	0.23	0.09	0.02	0.44	2106	--

**GS**	Psych distress	T3	Live Alone	0.15	0.08	-0.05	0.34	1932	--
**GS**	Life satisfaction	T0	Live Alone	--	--	--	--	--	No T0 Life Satisfaction
**GS**	Life satisfaction	T1	Live Alone	0.36	0.06	0.20	0.51	2982	--
**GS**	Life satisfaction	T2	Live Alone	0.44	0.10	0.21	0.66	2184	--
**GS**	Life satisfaction	T3	Live Alone	0.38	0.07	0.20	0.56	2024	--
**GS**	Psych distress	T0	Live Alone	0.21	0.10	-0.02	0.43	2984	sex, age, education, ethnicity, tenure, occupation, urbanicity
**GS**	Psych distress	T1	Live Alone	0.16	0.07	-0.02	0.34	2882	sex, age, education, ethnicity, tenure, occupation, urbanicity
**GS**	Psych distress	T2	Live Alone	0.25	0.09	0.03	0.46	2106	sex, age, education, ethnicity, tenure, occupation, urbanicity
**GS**	Psych distress	T3	Live Alone	0.13	0.08	-0.07	0.32	1932	sex, age, education, ethnicity, tenure, occupation, urbanicity
**GS**	Life satisfaction	T0	Live Alone	--	--	--	--	--	No T0 Life Satisfaction
**GS**	Life satisfaction	T1	Live Alone	0.34	0.06	0.18	0.50	2982	sex, age, education, ethnicity, tenure, occupation, urbanicity
**GS**	Life satisfaction	T2	Live Alone	0.45	0.10	0.21	0.68	2184	sex, age, education, ethnicity, tenure, occupation, urbanicity
**GS**	Life satisfaction	T3	Live Alone	0.36	0.07	0.17	0.54	2024	sex, age, education, ethnicity, tenure, occupation, urbanicity
**USOC**	Psych distress	T0	T0 living alone	0.06	0.04	-0.02	0.15	22187	--
**USOC**	Psych distress	T1	T1 living alone	0.19	0.05	0.10	0.28	12088	--
**USOC**	Psych distress	T2	T1 living alone	0.31	0.06	0.20	0.43	9905	--
**USOC**	Psych distress	T3	T1 living alone	0.16	0.05	0.06	0.27	9913	--
**USOC**	Life satisfaction	T0	T0 living alone	0.13	0.04	0.06	0.20	22187	--
**USOC**	Life satisfaction	T1	T1 living alone	0.33	0.04	0.25	0.42	10132	--
**USOC**	Life satisfaction	T2	T1 living alone	0.32	0.04	0.24	0.40	9905	--
**USOC**	Life satisfaction	T3	T1 living alone	0.31	0.03	0.24	0.38	9913	--
**USOC**	Psych distress	T0	T0 living alone	0.04	0.04	-0.04	0.13	22187	sex, age, education, ethnicity, UK nation, rural, social class, tenure, disability, illness
**USOC**	Psych distress	T1	T1 living alone	0.29	0.05	0.20	0.38	12088	sex, age, education, ethnicity, UK nation, rural, social class, tenure, disability, illness
**USOC**	Psych distress	T2	T1 living alone	0.31	0.06	0.19	0.43	9905	sex, age, education, ethnicity, UK nation, rural, social class, tenure, disability, illness
**USOC**	Psych distress	T3	T1 living alone	0.18	0.05	0.07	0.29	9913	sex, age, education, ethnicity, UK nation, rural, social class, tenure, disability, illness
**USOC**	Life satisfaction	T0	T0 living alone	0.04	0.04	-0.03	0.11	22187	sex, age, education, ethnicity, UK nation, rural, social class, tenure, disability, illness
**USOC**	Life satisfaction	T1	T1 living alone	0.29	0.04	0.20	0.37	10132	sex, age, education, ethnicity, UK nation, rural, social class, tenure, disability, illness
**USOC**	Life satisfaction	T2	T1 living alone	0.23	0.04	0.15	0.31	9905	sex, age, education, ethnicity, UK nation, rural, social class, tenure, disability, illness
**USOC**	Life satisfaction	T3	T1 living alone	0.23	0.04	0.16	0.30	9913	sex, age, education, ethnicity, UK nation, rural, social class, tenure, disability, illness
**ELSA**	Psych distress	T0	T0 living alone	0.75	0.09	0.58	0.93	5471	**--**
**ELSA**	Psych distress	T0	T0 living alone	0.50	0.09	0.32	0.67	5471	Sex ; Age; Education; Ethnicity, Occupational class; Home ownership; Disability; Chr
**ELSA**	Psych distress	T1	T1 living alone	0.48	0.07	0.35	0.61	5470	**--**

**ELSA**	Psych distress	T1	T1 living alone	0.31	0.06	0.19	0.44	5470	Sex ; Age; Education; Ethnicity, Occupational class; Home ownership; Disability; Chr
**ELSA**	Psych distress	T3	T1 living alone	0.42	0.06	0.30	0.54	5046	**--**
**ELSA**	Psych distress	T3	T1 living alone	0.31	0.06	0.19	0.42	5046	Sex ; Age; Education; Ethnicity, Occupational class; Home ownership; Disability; Chr
**ELSA**	Low Life Satisfaction	T0	T0 living alone	0.49	0.06	0.37	0.60	5090	**--**
**ELSA**	Low Life Satisfaction	T0	T0 living alone	0.37	0.06	0.26	0.49	5090	Sex ; Age; Education; Ethnicity, Occupational class; Home ownership; Disability; Chr
**ELSA**	Low Life Satisfaction	T1	T1 living alone	0.21	0.05	0.11	0.31	5453	**--**
**ELSA**	Low Life Satisfaction	T1	T1 living alone	0.14	0.05	0.03	0.24	5453	Sex ; Age; Education; Ethnicity, Occupational class; Home ownership; Disability; Chr
**ELSA**	Low Life Satisfaction	T3	T1 living alone	0.24	0.05	0.14	0.34	5038	**--**
**ELSA**	Low Life Satisfaction	T3	T1 living alone	0.19	0.05	0.08	0.29	5038	Sex ; Age; Education; Ethnicity, Occupational class; Home ownership; Disability; Chr
**TwinsUK**	Psych distress	T0	Live Alone	0.27	0.25	-0.22	0.75	2327	--
**TwinsUK**	Psych distress	T1	Live Alone	-0.23	0.20	-0.62	0.16	2327	--
**TwinsUK**	Psych distress	T2	Live Alone	-0.06	0.26	-0.57	0.45	2026	--
**TwinsUK**	Psych distress	T3	Live Alone	0.13	0.24	-0.35	0.60	2148	--
**TwinsUK**	Life satisfaction	T0	Live Alone	-	-	-	-	-	
**TwinsUK**	Life satisfaction	T1	Live Alone	-0.04	0.06	-0.16	0.09	2327	
**TwinsUK**	Life satisfaction	T2	Live Alone	-0.13	0.07	-0.26	0.01	2026	
**TwinsUK**	Life satisfaction	T3	Live Alone	-0.03	0.06	-0.16	0.09	2155	
**TwinsUK**	Psych distress	T0	Live Alone	0.33	0.21	-0.08	0.74	2327	sex, age, ethnicity, edu, country, ruc, homeownership, disability, srh_pp_bin
**TwinsUK**	Psych distress	T1	Live Alone	-0.10	0.20	-0.49	0.30	2327	sex, age, ethnicity, edu, country, ruc, homeownership, disability, srh_pp_bin
**TwinsUK**	Psych distress	T2	Live Alone	-0.04	0.23	-0.49	0.40	2026	sex, age, ethnicity, edu, country, ruc, homeownership, disability, srh_pp_bin
**TwinsUK**	Psych distress	T3	Live Alone	0.33	0.23	-0.12	0.78	2148	sex, age, ethnicity, edu, country, ruc, homeownership, disability, srh_pp_bin
**TwinsUK**	Life satisfaction	T0	Live Alone	-	-	-	-		
**TwinsUK**	Life satisfaction	T1	Live Alone	-0.13	0.05	-0.24	-0.03	2327	sex, age, ethnicity, edu, country, ruc, homeownership, disability, srh_pp_bin
**TwinsUK**	Life satisfaction	T2	Live Alone	-0.17	0.06	-0.28	-0.06	2026	sex, age, ethnicity, edu, country, ruc, homeownership, disability, srh_pp_bin
**TwinsUK**	Life satisfaction	T3	Live Alone	-0.14	0.05	-0.24	-0.04	2155	sex, age, ethnicity, edu, country, ruc, homeownership, disability, srh_pp_bin
**ALSPAC-G1**	Psych distress	T0	Live Alone	0.39	0.15	0.09	0.69	2252	--
**ALSPAC-G1**	Psych distress	T1	Live Alone	0.31	0.17	-0.01	0.64	2793	--
**ALSPAC-G1**	Psych distress	T2	Live Alone	0.27	0.18	-0.08	0.61	2073	--
**ALSPAC-G1**	Psych distress	T3	Live Alone	0.43	0.15	0.12	0.73	2468	--
**ALSPAC-G1**	Life satisfaction	T0	Live Alone	0.39	0.18	0.04	0.75	2178	--

**ALSPAC-G1**	Life satisfaction	T1	Live Alone	0.31	0.13	0.06	0.56	2813	--
**ALSPAC-G1**	Life satisfaction	T2	Live Alone	0.26	0.14	-0.02	0.54	2080	--
**ALSPAC-G1**	Life satisfaction	T3	Live Alone	0.34	0.13	0.08	0.60	2474	--
**ALSPAC-G1**	Psych distress	T0	Live Alone	0.36	0.18	0.02	0.70	1809	sex, ethnicity, occupational class, education, chronic illness
**ALSPAC-G1**	Psych distress	T1	Live Alone	0.24	0.20	-0.15	0.64	2064	sex, ethnicity, occupational class, education, chronic illness
**ALSPAC-G1**	Psych distress	T2	Live Alone	0.24	0.21	-0.17	0.65	1610	sex, ethnicity, occupational class, education, chronic illness
**ALSPAC-G1**	Psych distress	T3	Live Alone	0.46	0.17	0.12	0.80	1919	sex, ethnicity, occupational class, education, chronic illness
**ALSPAC-G1**	Life satisfaction	T0	Live Alone	0.32	0.21	-0.09	0.73	1720	sex, ethnicity, occupational class, education, chronic illness
**ALSPAC-G1**	Life satisfaction	T1	Live Alone	0.36	0.14	0.08	0.64	2075	sex, ethnicity, occupational class, education, chronic illness
**ALSPAC-G1**	Life satisfaction	T2	Live Alone	0.22	0.17	-0.11	0.55	1616	sex, ethnicity, occupational class, education, chronic illness
**ALSPAC-G1**	Life satisfaction	T3	Live Alone	0.41	0.15	0.12	0.70	1920	sex, ethnicity, occupational class, education, chronic illness
**ALSPAC-G0**	Psych distress	T0	Live Alone	0.31	0.15	0.01	0.60	2773	--
**ALSPAC-G0**	Psych distress	T1	Live Alone	1.09	0.21	0.67	1.51	3284	--
**ALSPAC-G0**	Psych distress	T2	Live Alone	0.94	0.21	0.53	1.36	2827	--
**ALSPAC-G0**	Psych distress	T3	Live Alone	0.76	0.21	0.35	1.17	2886	--
**ALSPAC-G0**	Psych distress	T0	Live alone	0.28	0.16	-0.03	0.58	2702	sex, age, ethnicity, occupational class, education, chronic illness
**ALSPAC-G0**	Psych distress	T1	Live alone	0.97	0.23	0.52	1.43	3177	sex, age, ethnicity, occupational class, education, chronic illness
**ALSPAC-G0**	Psych distress	T2	Live alone	0.88	0.22	0.44	1.31	2746	sex, age, ethnicity, occupational class, education, chronic illness
**ALSPAC-G0**	Psych distress	T3	Live alone	0.75	0.22	0.32	1.18	2808	sex, age, ethnicity, occupational class, education, chronic illness

Note: TP = timepoint; coef = coefficient; se = standard error ; l_ci = lower 95% confidence interval ; u_ci = cupper 95% confidence interval

Table S5. Interactions effects from cross-sectional models with continuous outcomes

cohort	outcome (Z)	TP	exposure	modifier	modifier category 1	coef_interaction	coef_se	lower_ci	upper_ci	n
**NCDS**	Psych distress	0	0 living alone	loneliness at 0	lonely	-0.31	0.23	-0.76	0.13	3448
**NCDS**	Psych distress	0	0 living alone	shielding status at 1	shielding	-0.13	0.20	-0.52	0.26	3527
**NCDS**	Psych distress	0	0 living alone	sex	female	-0.21	0.12	-0.44	0.02	3551
**NCDS**	Psych distress	0	0 living alone	Prior mental ill-health	yes	-0.14	0.10	-0.34	0.07	3551
**NCDS**	Psych distress	1	1 living alone	loneliness at 1	lonely	-0.42	0.23	-0.88	0.03	3547
**NCDS**	Psych distress	1	1 living alone	shielding status at 1	shielding	-0.05	0.21	-0.47	0.37	3545
**NCDS**	Psych distress	1	1 living alone	sex	female	0.01	0.09	-0.18	0.19	3567
**NCDS**	Psych distress	1	1 living alone	Prior mental ill-health	yes	0.08	0.21	-0.33	0.49	3567
**NCDS**	Psych distress	2	1 living alone	loneliness at 1	lonely	-0.48	0.20	-0.87	-0.09	3581
**NCDS**	Psych distress	2	1 living alone	shielding status at 1	shielding	0.03	0.17	-0.30	0.37	3667
**NCDS**	Psych distress	2	1 living alone	sex	female	-0.07	0.08	-0.24	0.09	3692
**NCDS**	Psych distress	2	1 living alone	Prior mental ill-health	yes	-0.20	0.15	-0.50	0.10	3692
**NCDS**	Psych distress	3	1 living alone	loneliness at 1	lonely	-0.49	0.21	-0.90	-0.07	3414
**NCDS**	Psych distress	3	1 living alone	shielding status at 1	shielding	0.00	0.18	-0.36	0.36	3489
**NCDS**	Psych distress	3	1 living alone	sex	female	-0.03	0.08	-0.18	0.13	3512
**NCDS**	Psych distress	3	1 living alone	Prior mental ill-health	yes	-0.15	0.15	-0.44	0.15	3512
**NCDS**	Life satisfaction	0	1 living alone	loneliness at 1	lonely	0.29	0.19	-0.09	0.66	3448
**NCDS**	Life satisfaction	0	1 living alone	shielding status at 1	shielding	0.10	0.23	-0.36	0.55	3527
**NCDS**	Life satisfaction	0	1 living alone	sex	female	0.01	0.11	-0.20	0.23	3551
**NCDS**	Life satisfaction	0	1 living alone	Prior mental ill-health	yes	-0.11	0.15	-0.41	0.18	3551
**NCDS**	Life satisfaction	1	1 living alone	loneliness at 1	lonely	0.20	0.22	-0.23	0.62	3595
**NCDS**	Life satisfaction	1	1 living alone	shielding status at 1	shielding	0.14	0.22	-0.29	0.57	3600
**NCDS**	Life satisfaction	1	1 living alone	sex	female	0.14	0.12	-0.09	0.36	3623
**NCDS**	Life satisfaction	1	1 living alone	Prior mental ill-health	yes	-0.05	0.21	-0.46	0.37	3623
**NCDS**	Life satisfaction	2	1 living alone	loneliness at 1	lonely	0.22	0.19	-0.15	0.60	3585
**NCDS**	Life satisfaction	2	1 living alone	shielding status at 1	shielding	-0.12	0.20	-0.52	0.27	3671
**NCDS**	Life satisfaction	2	1 living alone	sex	female	-0.02	0.08	-0.19	0.15	3696
**NCDS**	Life satisfaction	2	1 living alone	Prior mental ill-health	yes	0.18	0.14	-0.09	0.46	3696
**NCDS**	Life satisfaction	3	1 living alone	loneliness at 1	lonely	0.10	0.20	-0.29	0.49	3420
**NCDS**	Life satisfaction	3	1 living alone	shielding status at 1	shielding	-0.08	0.20	-0.47	0.32	3496

**NCDS**	Life satisfaction	3	1 living alone	sex	female	-0.10	0.09	-0.28	0.07	3520
**NCDS**	Life satisfaction	3	1 living alone	Prior mental ill-health	yes	-0.01	0.14	-0.29	0.27	3520
**GS**	Psych distress	0	Live Alone	1 loneliness	lonely	0.06	0.19	-0.19	0.31	2968
**GS**	Psych distress	0	Live Alone	shielding	shielding status at 1	-0.06	0.26	-0.39	0.27	2984
**GS**	Psych distress	0	Live Alone	sex	female	0.02	0.09	-0.13	0.17	2984
**GS**	Psych distress	0	Live Alone	prepandemic MH	prior mental ill health	0.19	0.08	0.09	0.29	2984
**GS**	Psych distress	0	Live Alone	age group	35 - 44	-0.37	0.23	-0.74	0.01	2984
**GS**	Psych distress	0	Live Alone	age group	45 - 54	0.01	0.22	-0.33	0.34	2984
**GS**	Psych distress	0	Live Alone	age group	54 - 64	-0.19	0.20	-0.50	0.12	2984
**GS**	Psych distress	0	Live Alone	age group	65 - 74	-0.29	0.20	-0.59	0.02	2984
**GS**	Psych distress	0	Live Alone	age group	75+	-0.14	0.22	-0.54	0.26	2984
**GS**	Life satisfaction	1	Live Alone	1 loneliness	lonely	0.31	0.18	-0.05	0.66	2968
**GS**	Life satisfaction	1	Live Alone	shielding	shielding status at 1	0.04	0.25	-0.45	0.52	2982
**GS**	Life satisfaction	1	Live Alone	sex	female	0.02	0.11	-0.19	0.24	2982
**GS**	Life satisfaction	1	Live Alone	prepandemic MH	prior mental ill health	-0.02	0.13	-0.26	0.23	2982
**GS**	Life satisfaction	1	Live Alone	age group	35 - 44	0.15	0.28	-0.40	0.71	2982
**GS**	Life satisfaction	1	Live Alone	age group	45 - 54	-0.20	0.27	-0.69	0.29	2982
**GS**	Life satisfaction	1	Live Alone	age group	54 - 64	0.19	0.25	-0.26	0.64	2982
**GS**	Life satisfaction	1	Live Alone	age group	65 - 74	0.16	0.24	-0.29	0.60	2982
**GS**	Life satisfaction	1	Live Alone	age group	75+	0.55	0.30	-0.04	1.14	2982
**GS**	Psych distress	1	Live Alone	1 loneliness	lonely	-0.36	0.28	-0.72	0.00	2871
**GS**	Psych distress	1	Live Alone	shielding	shielding status at 1	0.10	0.31	-0.40	0.60	2882
**GS**	Psych distress	1	Live Alone	sex	female	-0.16	0.13	-0.39	0.06	2882
**GS**	Psych distress	1	Live Alone	prepandemic MH	prior mental ill health	0.10	0.18	-0.15	0.35	2882
**GS**	Psych distress	1	Live Alone	age group	35 - 44	-0.36	0.41	-0.94	0.22	2882
**GS**	Psych distress	1	Live Alone	age group	45 - 54	-0.20	0.37	-0.73	0.32	2882
**GS**	Psych distress	1	Live Alone	age group	54 - 64	-0.40	0.34	-0.89	0.08	2882
**GS**	Psych distress	1	Live Alone	age group	65 - 74	-0.65	0.33	-1.12	-0.17	2882
**GS**	Psych distress	1	Live Alone	age group	75+	-0.60	0.37	-1.22	0.02	2882
**GS**	Life satisfaction	2	Live Alone	1 loneliness	lonely	0.25	0.24	-0.14	0.64	2172
**GS**	Life satisfaction	2	Live Alone	shielding	shielding status at 1	-0.06	0.26	-0.55	0.43	2184
**GS**	Life satisfaction	2	Live Alone	sex	female	0.00	0.12	-0.22	0.23	2184

**GS**	Life satisfaction	2	Live Alone	prepandemic MH	prior mental ill health	0.07	0.15	-0.17	0.32	2184
**GS**	Life satisfaction	2	Live Alone	age group	35 - 44	0.10	0.37	-0.55	0.75	2184
**GS**	Life satisfaction	2	Live Alone	age group	45 - 54	-0.12	0.36	-0.69	0.45	2184
**GS**	Life satisfaction	2	Live Alone	age group	54 - 64	-0.02	0.34	-0.56	0.53	2184
**GS**	Life satisfaction	2	Live Alone	age group	65 - 74	0.13	0.33	-0.41	0.66	2184
**GS**	Life satisfaction	2	Live Alone	age group	75+	0.37	0.36	-0.30	1.03	2184
**GS**	Psych distress	2	Live Alone	1 loneliness	lonely	-0.71	0.33	-1.15	-0.26	2096
**GS**	Psych distress	2	Live Alone	shielding	shielding status at 1	0.41	0.39	-0.14	0.97	2106
**GS**	Psych distress	2	Live Alone	sex	female	-0.17	0.15	-0.42	0.09	2106
**GS**	Psych distress	2	Live Alone	prepandemic MH	prior mental ill health	-0.07	0.20	-0.35	0.20	2106
**GS**	Psych distress	2	Live Alone	age group	35 - 44	-0.58	0.55	-1.34	0.18	2106
**GS**	Psych distress	2	Live Alone	age group	45 - 54	-0.39	0.50	-1.06	0.29	2106
**GS**	Psych distress	2	Live Alone	age group	54 - 64	-0.50	0.49	-1.14	0.14	2106
**GS**	Psych distress	2	Live Alone	age group	65 - 74	-0.66	0.48	-1.28	-0.03	2106
**GS**	Psych distress	2	Live Alone	age group	75+	-0.86	0.50	-1.63	-0.09	2106
**GS**	Life satisfaction	3	Live Alone	1 loneliness	lonely	0.49	0.25	0.05	0.93	2013
**GS**	Life satisfaction	3	Live Alone	shielding	shielding status at 1	-0.43	0.31	-1.00	0.14	2024
**GS**	Life satisfaction	3	Live Alone	sex	female	0.03	0.13	-0.22	0.29	2024
**GS**	Life satisfaction	3	Live Alone	prepandemic MH	prior mental ill health	-0.07	0.15	-0.36	0.22	2024
**GS**	Life satisfaction	3	Live Alone	age group	35 - 44	-0.05	0.35	-0.74	0.65	2024
**GS**	Life satisfaction	3	Live Alone	age group	45 - 54	-0.31	0.34	-0.91	0.29	2024
**GS**	Life satisfaction	3	Live Alone	age group	54 - 64	-0.27	0.32	-0.83	0.29	2024
**GS**	Life satisfaction	3	Live Alone	age group	65 - 74	-0.15	0.31	-0.70	0.40	2024
**GS**	Life satisfaction	3	Live Alone	age group	75+	-0.01	0.35	-0.74	0.72	2024
**GS**	Psych distress	3	Live Alone	1 loneliness	lonely	-0.45	0.32	-0.94	0.03	1923
**GS**	Psych distress	3	Live Alone	shielding	shielding status at 1	0.30	0.47	-0.35	0.95	1932
**GS**	Psych distress	3	Live Alone	sex	female	-0.27	0.16	-0.56	0.02	1932
**GS**	Psych distress	3	Live Alone	prepandemic MH	prior mental ill health	0.06	0.21	-0.25	0.37	1932
**GS**	Psych distress	3	Live Alone	age group	35 - 44	0.49	0.46	-0.28	1.26	1932
**GS**	Psych distress	3	Live Alone	age group	45 - 54	0.31	0.39	-0.36	0.97	1932
**GS**	Psych distress	3	Live Alone	age group	54 - 64	0.43	0.38	-0.19	1.06	1932
**GS**	Psych distress	3	Live Alone	age group	65 - 74	0.21	0.37	-0.41	0.82	1932
**GS**	Psych distress	3	Live Alone	age group	75+	0.14	0.41	-0.71	0.99	1932

**USOC**	Psych distress	0	Live Alone	shielding	shielding status at 1	0.21	0.13	-0.05	0.46	12088
**USOC**	Life satisfaction	0	Live Alone	shielding	shielding status at 1	-0.07	0.12	-0.30	0.16	12088
**USOC**	Life satisfaction	1	Live Alone	shielding	shielding status at 1	-0.18	0.15	-0.48	0.12	10132
**USOC**	Psych distress	0	Live Alone	0 loneliness	lonely	-0.20	0.09	-0.37	-0.03	22187
**USOC**	Life satisfaction	0	Live Alone	0 loneliness	lonely	0.03	0.07	-0.11	0.18	22187
**USOC**	Life satisfaction	1	Live Alone	0 loneliness	lonely	0.04	0.11	-0.19	0.26	10132
**USOC**	Psych distress	0	Live Alone	sex	female	-0.04	0.04	-0.13	0.04	22187
**USOC**	Life satisfaction	0	Live Alone	sex	female	0.04	0.04	-0.04	0.12	22187
**USOC**	Life satisfaction	1	Live Alone	sex	female	0.09	0.08	-0.07	0.26	10132
**USOC**	Psych distress	0	Live Alone	age group	16-24	0.20	0.16	-0.11	0.52	22187
**USOC**	Psych distress	0	Live Alone	age group	25-34	-0.06	0.11	-0.27	0.15	22187
**USOC**	Psych distress	0	Live Alone	age group	35-44	-0.05	0.11	-0.27	0.17	22187
**USOC**	Psych distress	0	Live Alone	age group	55-64	0.09	0.08	-0.07	0.25	22187
**USOC**	Psych distress	0	Live Alone	age group	65-74	-0.08	0.07	-0.22	0.06	22187
**USOC**	Psych distress	0	Live Alone	age group	75+	-0.18	0.07	-0.32	-0.04	22187
**USOC**	Life satisfaction	0	Live Alone	age group	16-24	-0.03	0.14	-0.31	0.25	22187
**USOC**	Life satisfaction	0	Live Alone	age group	25-34	0.09	0.09	-0.09	0.27	22187
**USOC**	Life satisfaction	0	Live Alone	age group	35-44	-0.03	0.11	-0.24	0.18	22187
**USOC**	Life satisfaction	0	Live Alone	age group	55-64	-0.05	0.08	-0.21	0.11	22187
**USOC**	Life satisfaction	0	Live Alone	age group	65-74	0.05	0.07	-0.09	0.19	22187
**USOC**	Life satisfaction	0	Live Alone	age group	75+	0.20	0.08	0.05	0.36	22187
**USOC**	Life satisfaction	1	Live Alone	age group	16-24	0.18	0.35	-0.51	0.87	10132
**USOC**	Life satisfaction	1	Live Alone	age group	25-34	-0.40	0.20	-0.80	-0.01	10132
**USOC**	Life satisfaction	1	Live Alone	age group	35-44	-0.39	0.17	-0.72	-0.05	10132
**USOC**	Life satisfaction	1	Live Alone	age group	55-64	-0.10	0.13	-0.35	0.16	10132
**USOC**	Life satisfaction	1	Live Alone	age group	65-74	-0.14	0.13	-0.38	0.11	10132
**USOC**	Life satisfaction	1	Live Alone	age group	75+	0.09	0.15	-0.20	0.38	10132
**USOC**	Life satisfaction	1	Live Alone	prepandemic MH	prior mental ill-health	-0.14	0.10	-0.33	0.05	10132
**USOC**	Psych distress	1	Live Alone	shielding	shielding status at 1	0.10	0.12	-0.13	0.32	12088
**USOC**	Psych distress	2	Live Alone	shielding	shielding status at 1	0.12	0.13	-0.13	0.37	9905
**USOC**	Psych distress	3	Live Alone	shielding	shielding status at 1	0.02	0.12	-0.22	0.26	9913
**USOC**	Life satisfaction	2	Live Alone	shielding	shielding status at 1	-0.01	0.11	-0.23	0.21	9905

**USOC**	Life satisfaction	3	Live Alone	shielding	shielding status at 1	-0.07	0.10	-0.27	0.13	9913
**USOC**	Psych distress	1	Live Alone	1 loneliness	lonely	-0.08	0.09	-0.26	0.11	12088
**USOC**	Psych distress	2	Live Alone	1 loneliness	lonely	0.01	0.11	-0.21	0.24	9905
**USOC**	Psych distress	3	Live Alone	1 loneliness	lonely	-0.05	0.11	-0.27	0.17	9913
**USOC**	Life satisfaction	2	Live Alone	1 loneliness	lonely	-0.08	0.09	-0.26	0.09	9905
**USOC**	Life satisfaction	3	Live Alone	1 loneliness	lonely	0.02	0.08	-0.15	0.18	9913
**USOC**	Psych distress	1	Live Alone	sex	female	-0.07	0.06	-0.19	0.04	12088
**USOC**	Psych distress	2	Live Alone	sex	female	-0.09	0.06	-0.21	0.03	9905
**USOC**	Psych distress	3	Live Alone	sex	female	-0.03	0.06	-0.15	0.09	9913
**USOC**	Life satisfaction	2	Live Alone	sex	female	0.17	0.06	0.05	0.29	9905
**USOC**	Life satisfaction	3	Live Alone	sex	female	0.18	0.06	0.07	0.29	9913
**USOC**	Psych distress	1	Live Alone	age group	16-24	-0.49	0.37	-1.21	0.24	12088
**USOC**	Psych distress	1	Live Alone	age group	25-34	0.10	0.16	-0.21	0.42	12088
**USOC**	Psych distress	1	Live Alone	age group	35-44	-0.13	0.15	-0.43	0.17	12088
**USOC**	Psych distress	1	Live Alone	age group	55-64	-0.07	0.10	-0.26	0.13	12088
**USOC**	Psych distress	1	Live Alone	age group	65-74	-0.06	0.10	-0.25	0.12	12088
**USOC**	Psych distress	1	Live Alone	age group	75+	-0.16	0.10	-0.36	0.05	12088
**USOC**	Psych distress	2	Live Alone	age group	16-24	-0.50	0.32	-1.13	0.13	9905
**USOC**	Psych distress	2	Live Alone	age group	25-34	0.28	0.18	-0.08	0.64	9905
**USOC**	Psych distress	2	Live Alone	age group	35-44	0.03	0.17	-0.31	0.37	9905
**USOC**	Psych distress	2	Live Alone	age group	55-64	-0.05	0.10	-0.25	0.15	9905
**USOC**	Psych distress	2	Live Alone	age group	65-74	-0.07	0.10	-0.27	0.12	9905
**USOC**	Psych distress	2	Live Alone	age group	75+	-0.18	0.11	-0.40	0.03	9905
**USOC**	Psych distress	3	Live Alone	age group	16-24	-0.37	0.41	-1.17	0.42	9913
**USOC**	Psych distress	3	Live Alone	age group	25-34	0.00	0.17	-0.34	0.34	9913
**USOC**	Psych distress	3	Live Alone	age group	35-44	-0.04	0.16	-0.35	0.26	9913
**USOC**	Psych distress	3	Live Alone	age group	55-64	-0.06	0.10	-0.26	0.15	9913
**USOC**	Psych distress	3	Live Alone	age group	65-74	-0.04	0.10	-0.24	0.16	9913
**USOC**	Psych distress	3	Live Alone	age group	75+	-0.09	0.11	-0.31	0.12	9913
**USOC**	Life satisfaction	2	Live Alone	age group	16-24	0.28	0.26	-0.22	0.78	9905
**USOC**	Life satisfaction	2	Live Alone	age group	25-34	-0.17	0.13	-0.43	0.09	9905
**USOC**	Life satisfaction	2	Live Alone	age group	35-44	-0.23	0.14	-0.50	0.03	9905
**USOC**	Life satisfaction	2	Live Alone	age group	55-64	-0.02	0.10	-0.21	0.17	9905

McElroy E, et al. BMJ Ment Health 2023; 26:e300842. doi: 10.1136/bmjment-2023-300842

**USOC**	Life satisfaction	2	Live Alone	age group	65-74	0.13	0.09	-0.05	0.31	9905
**USOC**	Life satisfaction	2	Live Alone	age group	75+	0.33	0.11	0.11	0.54	9905
**USOC**	Life satisfaction	3	Live Alone	age group	16-24	0.43	0.24	-0.04	0.89	9913
**USOC**	Life satisfaction	3	Live Alone	age group	25-34	0.00	0.13	-0.25	0.24	9913
**USOC**	Life satisfaction	3	Live Alone	age group	35-44	-0.06	0.12	-0.30	0.19	9913
**USOC**	Life satisfaction	3	Live Alone	age group	55-64	-0.05	0.09	-0.22	0.13	9913
**USOC**	Life satisfaction	3	Live Alone	age group	65-74	0.05	0.09	-0.12	0.23	9913
**USOC**	Life satisfaction	3	Live Alone	age group	75+	0.06	0.10	-0.14	0.26	9913
**USOC**	Psych distress	1	Live Alone	prepandemic MH	prior mental ill-health	0.14	0.08	-0.02	0.30	12088
**USOC**	Psych distress	2	Live Alone	prepandemic MH	prior mental ill-health	0.22	0.09	0.05	0.40	9905
**USOC**	Psych distress	3	Live Alone	prepandemic MH	prior mental ill-health	0.13	0.09	-0.05	0.30	9913
**USOC**	Life satisfaction	2	Live Alone	prepandemic MH	prior mental ill-health	-0.26	0.07	-0.40	-0.11	9905
**USOC**	Life satisfaction	3	Live Alone	prepandemic MH	prior mental ill-health	-0.19	0.07	-0.32	-0.05	9913
**BCS**	Psych distress	0	0 living alone	loneliness at 0	lonely	-0.34	0.26	-0.84	0.16	2405
**BCS**	Psych distress	0	0 living alone	shielding status at 1	shielding	0.09	0.31	-0.51	0.69	2455
**BCS**	Psych distress	0	0 living alone	sex	female	0.22	0.14	-0.06	0.49	2461
**BCS**	Psych distress	0	0 living alone	Prior mental ill-health	yes	-0.02	0.12	-0.25	0.21	2461
**BCS**	Psych distress	1	1 living alone	loneliness at 1	lonely	-0.43	0.20	-0.83	-0.03	2608
**BCS**	Psych distress	1	1 living alone	shielding status at 1	shielding	-0.10	0.36	-0.81	0.61	2609
**BCS**	Psych distress	1	1 living alone	sex	female	0.00	0.13	-0.26	0.27	2616
**BCS**	Psych distress	1	1 living alone	Prior mental ill-health	yes	-0.12	0.18	-0.47	0.24	2616
**BCS**	Psych distress	2	1 living alone	loneliness at 1	lonely	-0.42	0.22	-0.85	0.02	2611
**BCS**	Psych distress	2	1 living alone	shielding status at 1	shielding	0.52	0.24	0.05	0.99	2666
**BCS**	Psych distress	2	1 living alone	sex	female	0.00	0.13	-0.25	0.25	2673
**BCS**	Psych distress	2	1 living alone	Prior mental ill-health	yes	0.28	0.15	-0.03	0.58	2673
**BCS**	Psych distress	3	1 living alone	loneliness at 1	lonely	-0.17	0.22	-0.60	0.26	2356
**BCS**	Psych distress	3	1 living alone	shielding status at 1	shielding	0.32	0.28	-0.23	0.88	2401
**BCS**	Psych distress	3	1 living alone	sex	female	0.00	0.13	-0.24	0.25	2406
**BCS**	Psych distress	3	1 living alone	Prior mental ill-health	yes	0.20	0.17	-0.13	0.52	2406
**BCS**	Life satisfaction	0	1 living alone	loneliness at 1	lonely	-0.37	0.23	-0.83	0.08	2401
**BCS**	Life satisfaction	0	1 living alone	shielding status at 1	shielding	0.31	0.31	-0.29	0.92	2451
**BCS**	Life satisfaction	0	1 living alone	sex	female	0.05	0.13	-0.22	0.31	2457

**BCS**	Life satisfaction	0	1 living alone	Prior mental ill-health	yes	-0.25	0.19	-0.62	0.12	2457
**BCS**	Life satisfaction	1	1 living alone	loneliness at 1	lonely	-0.06	0.24	-0.53	0.40	2628
**BCS**	Life satisfaction	1	1 living alone	shielding status at 1	shielding	0.03	0.44	-0.83	0.88	2631
**BCS**	Life satisfaction	1	1 living alone	sex	female	0.01	0.16	-0.31	0.33	2638
**BCS**	Life satisfaction	1	1 living alone	Prior mental ill-health	yes	-0.08	0.21	-0.49	0.32	2638
**BCS**	Life satisfaction	2	1 living alone	loneliness at 1	lonely	-0.15	0.20	-0.54	0.25	2616
**BCS**	Life satisfaction	2	1 living alone	shielding status at 1	shielding	-0.29	0.26	-0.80	0.23	2671
**BCS**	Life satisfaction	2	1 living alone	sex	female	0.18	0.12	-0.05	0.42	2678
**BCS**	Life satisfaction	2	1 living alone	Prior mental ill-health	yes	-0.05	0.16	-0.36	0.26	2678
**BCS**	Life satisfaction	3	1 living alone	loneliness at 1	lonely	-0.06	0.22	-0.50	0.38	2362
**BCS**	Life satisfaction	3	1 living alone	shielding status at 1	shielding	-0.11	0.28	-0.66	0.45	2407
**BCS**	Life satisfaction	3	1 living alone	sex	female	0.15	0.13	-0.10	0.41	2412
**BCS**	Life satisfaction	3	1 living alone	Prior mental ill-health	yes	0.05	0.18	-0.29	0.40	2412
**NS**	Psych distress	0	0 living alone	loneliness at 0	lonely	0.05	0.32	-0.58	0.68	1080
**NS**	Psych distress	0	0 living alone	shielding status at 1	shielding	-0.47	0.36	-1.18	0.24	1110
**NS**	Psych distress	0	0 living alone	sex	female	-0.40	0.15	-0.70	-0.10	1114
**NS**	Psych distress	0	0 living alone	Prior mental ill-health	yes	0.17	0.14	-0.11	0.44	1114
**NS**	Psych distress	1	1 living alone	loneliness at 1	lonely	-0.15	0.36	-0.86	0.55	1135
**NS**	Psych distress	1	1 living alone	shielding status at 1	shielding	0.13	0.42	-0.70	0.97	1131
**NS**	Psych distress	1	1 living alone	sex	female	0.09	0.22	-0.35	0.52	1135
**NS**	Psych distress	1	1 living alone	Prior mental ill-health	yes	-0.03	0.28	-0.58	0.52	1135
**NS**	Psych distress	2	1 living alone	loneliness at 1	lonely	-0.61	0.28	-1.16	-0.06	1141
**NS**	Psych distress	2	1 living alone	shielding status at 1	shielding	-0.27	0.51	-1.27	0.73	1178
**NS**	Psych distress	2	1 living alone	sex	female	-0.14	0.18	-0.49	0.22	1182
**NS**	Psych distress	2	1 living alone	Prior mental ill-health	yes	0.03	0.23	-0.42	0.48	1182
**NS**	Psych distress	3	1 living alone	loneliness at 1	lonely	-0.64	0.28	-1.18	-0.10	1071
**NS**	Psych distress	3	1 living alone	shielding status at 1	shielding	-0.15	0.58	-1.30	0.99	1099
**NS**	Psych distress	3	1 living alone	sex	female	-0.26	0.18	-0.61	0.09	1103
**NS**	Psych distress	3	1 living alone	Prior mental ill-health	yes	-0.05	0.23	-0.51	0.41	1103
**NS**	Life satisfaction	0	1 living alone	loneliness at 1	lonely	0.00	0.08	-0.17	0.16	1080
**NS**	Life satisfaction	0	1 living alone	shielding status at 1	shielding	-0.25	0.07	-0.40	-0.11	1110
**NS**	Life satisfaction	0	1 living alone	sex	female	-0.07	0.04	-0.15	0.01	1114

**NS**	Life satisfaction	0	1 living alone	Prior mental ill-health	yes	0.04	0.05	-0.06	0.14	1114
**NS**	Life satisfaction	1	1 living alone	loneliness at 1	lonely	-0.17	0.26	-0.68	0.35	1157
**NS**	Life satisfaction	1	1 living alone	shielding status at 1	shielding	0.10	0.29	-0.47	0.68	1154
**NS**	Life satisfaction	1	1 living alone	sex	female	0.04	0.20	-0.35	0.44	1158
**NS**	Life satisfaction	1	1 living alone	Prior mental ill-health	yes	0.31	0.24	-0.16	0.79	1158
**NS**	Life satisfaction	2	1 living alone	loneliness at 1	lonely	0.15	0.17	-0.19	0.48	1145
**NS**	Life satisfaction	2	1 living alone	shielding status at 1	shielding	-0.01	0.29	-0.58	0.56	1183
**NS**	Life satisfaction	2	1 living alone	sex	female	-0.03	0.11	-0.25	0.18	1187
**NS**	Life satisfaction	2	1 living alone	Prior mental ill-health	yes	0.01	0.15	-0.29	0.31	1187
**NS**	Life satisfaction	3	1 living alone	loneliness at 1	lonely	0.29	0.18	-0.06	0.64	1074
**NS**	Life satisfaction	3	1 living alone	shielding status at 1	shielding	0.22	0.28	-0.33	0.76	1102
**NS**	Life satisfaction	3	1 living alone	sex	female	0.25	0.12	0.01	0.50	1106
**NS**	Life satisfaction	3	1 living alone	Prior mental ill-health	yes	-0.07	0.16	-0.38	0.24	1106
**ELSA**	Psych distress	0	0 living alone	shielding status at 1	shielding	-0.09	0.12	-0.32	0.15	5463
**ELSA**	Psych distress	0	0 living alone	lionliness 0	often lonely	-0.26	0.20	-0.66	0.14	5117
**ELSA**	Psych distress	0	0 living alone	sex	female	0.16	0.09	-0.03	0.34	5470
**ELSA**	Psych distress	0	0 living alone	age group	55-64	-0.05	0.20	-0.43	0.34	5470
**ELSA**	Psych distress	0	0 living alone	age group	65-74	-0.15	0.17	-0.49	0.20	5470
**ELSA**	Psych distress	0	0 living alone	age group	75+	-0.13	0.18	-0.48	0.23	5470
**ELSA**	Psych distress	1	1 living alone	shielding status at 1	shielding	-0.08	0.10	-0.29	0.12	5463
**ELSA**	Psych distress	1	1 living alone	lionliness 1	often lonely	-0.14	0.13	-0.39	0.11	5465
**ELSA**	Psych distress	1	1 living alone	sex	female	0.05	0.09	-0.13	0.23	5470
**ELSA**	Psych distress	1	1 living alone	age group	55-64	-0.17	0.20	-0.57	0.22	5470
**ELSA**	Psych distress	1	1 living alone	age group	65-74	-0.16	0.18	-0.51	0.19	5470
**ELSA**	Psych distress	1	1 living alone	age group	75+	-0.32	0.18	-0.67	0.03	5470
**ELSA**	Psych distress	1	1 living alone	prior mental ill-health	depressed at 0	-0.05	0.12	-0.29	0.19	5470
**ELSA**	Psych distress	3	1 living alone	shielding status at 1	shielding	0.06	0.10	-0.14	0.26	5040
**ELSA**	Psych distress	3	1 living alone	lionliness 1	often lonely	0.12	0.13	-0.14	0.38	5042
**ELSA**	Psych distress	3	1 living alone	sex	female	-0.08	0.09	-0.25	0.09	5046
**ELSA**	Psych distress	3	1 living alone	age group	55-64	0.12	0.19	-0.26	0.50	5046
**ELSA**	Psych distress	3	1 living alone	age group	65-74	-0.01	0.18	-0.36	0.34	5046
**ELSA**	Psych distress	3	1 living alone	age group	75+	-0.12	0.18	-0.47	0.23	5046

**ELSA**	Psych distress	3	1 living alone	prior mental ill-health	depressed at 0	-0.06	0.11	-0.27	0.16	5046
**ELSA**	Life satisfaction	0	0 living alone	shielding status at 1	shielding	0.03	0.12	-0.21	0.26	5085
**ELSA**	Life satisfaction	0	0 living alone	lionliness 0	often lonely	0.27	0.20	-0.11	0.65	5058
**ELSA**	Life satisfaction	0	0 living alone	sex	female	-0.05	0.09	-0.23	0.14	5090
**ELSA**	Life satisfaction	0	0 living alone	age group	55-64	0.09	0.21	-0.32	0.51	5090
**ELSA**	Life satisfaction	0	0 living alone	age group	65-74	0.02	0.20	-0.36	0.40	5090
**ELSA**	Life satisfaction	0	0 living alone	age group	75+	0.12	0.20	-0.27	0.51	5090
**ELSA**	Life satisfaction	0	1 living alone	prior mental ill-health	depressed at 0	0.18	0.14	-0.10	0.46	5090
**ELSA**	Life satisfaction	1	1 living alone	shielding status at 1	shielding	0.01	0.11	-0.20	0.23	5446
**ELSA**	Life satisfaction	1	1 living alone	lionliness 1	often lonely	0.22	0.16	-0.10	0.54	5448
**ELSA**	Life satisfaction	1	1 living alone	sex	female	0.04	0.08	-0.13	0.20	5453
**ELSA**	Life satisfaction	1	1 living alone	age group	55-64	0.02	0.21	-0.38	0.43	5453
**ELSA**	Life satisfaction	1	1 living alone	age group	65-74	-0.09	0.19	-0.47	0.28	5453
**ELSA**	Life satisfaction	1	1 living alone	age group	75+	0.19	0.19	-0.19	0.57	5453
**ELSA**	Life satisfaction	1	1 living alone	prior mental ill-health	depressed at 0	0.18	0.12	-0.06	0.41	5453
**ELSA**	Life satisfaction	3	1 living alone	shielding status at 1	shielding	-0.03	0.11	-0.25	0.18	5031
**ELSA**	Life satisfaction	3	1 living alone	lionliness 1	often lonely	-0.01	0.17	-0.35	0.33	5034
**ELSA**	Life satisfaction	3	1 living alone	sex	female	0.01	0.09	-0.17	0.20	5038
**ELSA**	Life satisfaction	3	1 living alone	age group	55-64	-0.16	0.21	-0.58	0.25	5038
**ELSA**	Life satisfaction	3	1 living alone	age group	65-74	-0.12	0.19	-0.50	0.25	5038
**ELSA**	Life satisfaction	3	1 living alone	age group	75+	0.01	0.19	-0.37	0.39	5038
**ELSA**	Life satisfaction	3	1 living alone	prior mental ill-health	depressed at 0	0.18	0.14	-0.09	0.45	5038
**TwinsUK**	Psych distress	0	Live Alone	1 loneliness	lonely	1.12	0.14	0.93	1.31	2325
**TwinsUK**	Psych distress	0	Live Alone	shielding	shielding status at 1	0.20	0.05	0.11	0.30	2326
**TwinsUK**	Psych distress	0	Live Alone	sex	female	0.25	0.07	0.09	0.40	2327
**TwinsUK**	Psych distress	0	Live Alone	prepandemic MH	prior mental ill health	2.48	0.09	2.32	2.63	2327
**TwinsUK**	Psych distress	0	Live Alone	age group	16-24	-0.36	0.53	-1.20	0.49	2327
**TwinsUK**	Psych distress	0	Live Alone	age group	25-34	0.15	0.24	-0.29	0.60	2327
**TwinsUK**	Psych distress	0	Live Alone	age group	35-44	-0.01	0.15	-0.27	0.25	2327
**TwinsUK**	Psych distress	0	Live Alone	age group	55-64	-0.10	0.11	-0.32	0.11	2327
**TwinsUK**	Psych distress	0	Live Alone	age group	65-74	-0.18	0.15	-0.49	0.12	2327
**TwinsUK**	Psych distress	0	Live Alone	age group	75+	-0.08	0.21	-0.52	0.35	2327

McElroy E, *et al. BMJ Ment Health* 2023; 26:e300842. doi: 10.1136/bmjment-2023-300842

**TwinsUK**	Psych distress	1	Live Alone	1 loneliness	lonely	1.65	0.13	1.45	1.86	2321
**TwinsUK**	Psych distress	1	Live Alone	shielding	shielding status at 1	0.23	0.06	0.12	0.33	2322
**TwinsUK**	Psych distress	1	Live Alone	sex	female	0.33	0.08	0.15	0.50	2323
**TwinsUK**	Psych distress	1	Live Alone	prepandemic MH	prior mental ill health	1.34	0.13	1.14	1.55	2323
**TwinsUK**	Psych distress	1	Live Alone	age group	16-24	-0.16	0.50	-1.11	0.78	2323
**TwinsUK**	Psych distress	1	Live Alone	age group	25-34	0.41	0.23	-0.09	0.90	2323
**TwinsUK**	Psych distress	1	Live Alone	age group	35-44	0.32	0.15	0.02	0.61	2323
**TwinsUK**	Psych distress	1	Live Alone	age group	55-64	0.01	0.12	-0.22	0.25	2323
**TwinsUK**	Psych distress	1	Live Alone	age group	65-74	-0.17	0.17	-0.52	0.17	2323
**TwinsUK**	Psych distress	1	Live Alone	age group	75+	-0.21	0.24	-0.70	0.27	2323
**TwinsUK**	Psych distress	2	Live Alone	1 loneliness	lonely	1.22	0.15	1.03	1.42	2321
**TwinsUK**	Psych distress	2	Live Alone	shielding	shielding status at 1	0.22	0.05	0.12	0.31	2019
**TwinsUK**	Psych distress	2	Live Alone	sex	female	0.35	0.07	0.19	0.51	2020
**TwinsUK**	Psych distress	2	Live Alone	prepandemic MH	prior mental ill health	1.33	0.12	1.15	1.51	2020
**TwinsUK**	Psych distress	2	Live Alone	age group	16-24	0.64	0.66	-0.30	1.58	2020
**TwinsUK**	Psych distress	2	Live Alone	age group	25-34	0.62	0.21	0.17	1.06	2020
**TwinsUK**	Psych distress	2	Live Alone	age group	35-44	0.31	0.14	0.05	0.58	2020
**TwinsUK**	Psych distress	2	Live Alone	age group	55-64	0.04	0.11	-0.18	0.25	2020
**TwinsUK**	Psych distress	2	Live Alone	age group	65-74	-0.07	0.16	-0.38	0.24	2020
**TwinsUK**	Psych distress	2	Live Alone	age group	75+	-0.09	0.22	-0.53	0.35	2020
**TwinsUK**	Psych distress	3	Live Alone	1 loneliness	lonely	1.12	0.12	0.93	1.31	2139
**TwinsUK**	Psych distress	3	Live Alone	shielding	shielding status at 1	0.22	0.05	0.12	0.31	2140
**TwinsUK**	Psych distress	3	Live Alone	sex	female	0.34	0.07	0.18	0.50	2141
**TwinsUK**	Psych distress	3	Live Alone	prepandemic MH	prior mental ill health	1.34	0.12	1.16	1.53	2141
**TwinsUK**	Psych distress	3	Live Alone	age group 45-54(ref)	16-24	-0.05	0.76	-1.01	0.91	2141
**TwinsUK**	Psych distress	3	Live Alone	age group	25-34	0.47	0.24	0.01	0.93	2141
**TwinsUK**	Psych distress	3	Live Alone	age group	35-44	0.13	0.14	-0.13	0.40	2141
**TwinsUK**	Psych distress	3	Live Alone	age group	55-64	-0.23	0.12	-0.45	-0.02	2141
**TwinsUK**	Psych distress	3	Live Alone	age group	65-74	-0.42	0.16	-0.73	-0.10	2141
**TwinsUK**	Psych distress	3	Live Alone	age group	75+	-0.45	0.22	-0.89	-0.01	2141
**TwinsUK**	Life satisfaction	1	Live Alone	1 loneliness	lonely	-1.21	0.12	-1.41	-1.00	2325
**TwinsUK**	Life satisfaction	1	Live Alone	shielding	shielding status at 1	-0.05	0.05	-0.15	0.05	2327
**TwinsUK**	Life satisfaction	1	Live Alone	sex	female	-0.11	0.07	-0.27	0.06	2327

McElroy E, *et al. BMJ Ment Health* 2023; 26:e300842. doi: 10.1136/bmjment-2023-300842

**TwinsUK**	Life satisfaction	1	Live Alone	prepandemic MH	prior mental ill health	-0.61	0.12	-0.82	-0.41	2327
**TwinsUK**	Life satisfaction	1	Live Alone	age group	16-24	-0.23	0.37	-1.13	0.66	2327
**TwinsUK**	Life satisfaction	1	Live Alone	age group	25-34	-0.42	0.23	-0.88	0.05	2327
**TwinsUK**	Life satisfaction	1	Live Alone	age group	35-44	-0.29	0.15	-0.57	-0.02	2327
**TwinsUK**	Life satisfaction	1	Live Alone	age group	55-64	-0.01	0.11	-0.23	0.21	2327
**TwinsUK**	Life satisfaction	1	Live Alone	age group	65-74	0.11	0.17	-0.21	0.43	2327
**TwinsUK**	Life satisfaction	1	Live Alone	age group	75+	0.20	0.23	-0.25	0.66	2327
**TwinsUK**	Life satisfaction	2	Live Alone	1 loneliness	lonely	-1.16	0.14	-1.38	-0.94	2024
**TwinsUK**	Life satisfaction	2	Live Alone	shielding	shielding status at 1	-0.16	0.06	-0.26	-0.05	2025
**TwinsUK**	Life satisfaction	2	Live Alone	sex	female	-0.26	0.08	-0.43	-0.09	2026
**TwinsUK**	Life satisfaction	2	Live Alone	prepandemic MH	prior mental ill health	-0.76	0.13	-0.97	-0.55	2026
**TwinsUK**	Life satisfaction	2	Live Alone	age group	16-24	-1.03	0.64	-2.04	-0.01	2026
**TwinsUK**	Life satisfaction	2	Live Alone	age group	25-34	-0.62	0.23	-1.10	-0.14	2026
**TwinsUK**	Life satisfaction	2	Live Alone	age group	35-44	-0.27	0.15	-0.55	0.02	2026
**TwinsUK**	Life satisfaction	2	Live Alone	age group	55-64	-0.02	0.12	-0.26	0.21	2026
**TwinsUK**	Life satisfaction	2	Live Alone	age group	65-74	0.02	0.17	-0.31	0.36	2026
**TwinsUK**	Life satisfaction	2	Live Alone	age group	75+	0.03	0.25	-0.45	0.51	2026
**TwinsUK**	Life satisfaction	3	Live Alone	1 loneliness	lonely	-0.99	0.13	-1.18	-0.79	2153
**TwinsUK**	Life satisfaction	3	Live Alone	shielding	shielding status at 1	-0.14	0.05	-0.24	-0.04	2154
**TwinsUK**	Life satisfaction	3	Live Alone	sex	female	-0.13	0.07	-0.28	0.03	2155
**TwinsUK**	Life satisfaction	3	Live Alone	prepandemic MH	prior mental ill health	-0.61	0.13	-0.81	-0.42	2155
**TwinsUK**	Life satisfaction	3	Live Alone	age group 45-54(ref)	16-24	0.05	0.47	-0.92	1.01	2155
**TwinsUK**	Life satisfaction	3	Live Alone	age group	25-34	-0.43	0.23	-0.89	0.03	2155
**TwinsUK**	Life satisfaction	3	Live Alone	age group	35-44	-0.16	0.14	-0.43	0.10	2155
**TwinsUK**	Life satisfaction	3	Live Alone	age group	55-64	0.04	0.11	-0.18	0.25	2155
**TwinsUK**	Life satisfaction	3	Live Alone	age group	65-74	0.14	0.16	-0.17	0.45	2155
**TwinsUK**	Life satisfaction	3	Live Alone	age group	75+	0.07	0.22	-0.36	0.51	2155
**ALSPAC-G1**	Psych distress	0	Live Alone	sex	female	0.23	0.19	-0.15	0.61	1809
**ALSPAC-G1**	Psych distress	0	Live Alone	prepandemic MH	prior mental ill health	-0.20	0.11	-0.41	0.01	1809
**ALSPAC-G1**	Psych distress	1	Live Alone	sex	female	0.09	0.19	-0.27	0.46	2064
**ALSPAC-G1**	Psych distress	1	Live Alone	prepandemic MH	prior mental ill health	0.11	0.18	-0.23	0.46	1774
**ALSPAC-G1**	Psych distress	2	Live Alone	sex	female	0.19	0.20	-0.21	0.59	1610

**ALSPAC-G1**	Psych distress	2	Live Alone	prepandemic MH	prior mental ill health	0.11	0.20	-0.27	0.50	1413
**ALSPAC-G1**	Psych distress	3	Live Alone	sex	female	0.14	0.19	-0.24	0.52	1919
**ALSPAC-G1**	Psych distress	3	Live Alone	prepandemic MH	prior mental ill health	0.29	0.18	-0.06	0.65	1675
**ALSPAC-G1**	Life satisfaction	0	Live Alone	sex	female	0.07	0.20	-0.32	0.46	1720
**ALSPAC-G1**	Life satisfaction	0	Live Alone	prepandemic MH	prior mental ill health	0.23	0.16	-0.08	0.54	1720
**ALSPAC-G1**	Life satisfaction	1	Live Alone	sex	female	0.05	0.19	-0.31	0.42	2075
**ALSPAC-G1**	Life satisfaction	1	Live Alone	prepandemic MH	prior mental ill health	0.37	0.21	-0.05	0.79	1696
**ALSPAC-G1**	Life satisfaction	2	Live Alone	sex	female	-0.20	0.21	-0.60	0.21	1616
**ALSPAC-G1**	Life satisfaction	2	Live Alone	prepandemic MH	prior mental ill health	0.39	0.24	-0.07	0.86	1351
**ALSPAC-G1**	Life satisfaction	3	Live Alone	sex	female	-0.11	0.20	-0.49	0.27	1920
**ALSPAC-G1**	Life satisfaction	3	Live Alone	prepandemic MH	prior mental ill health	0.13	0.22	-0.29	0.56	1600
**ALSPAC-G0**	Psych distress	0	Live Alone	sex	female	0.21	0.17	-0.12	0.54	2702
**ALSPAC-G0**	Psych distress	0	Live Alone	prepandemic MH	prior mental ill health	0.32	0.11	0.11	0.52	2702
**ALSPAC-G0**	Psych distress	1	Live Alone	sex	female	0.23	0.15	-0.07	0.54	3177
**ALSPAC-G0**	Psych distress	1	Live Alone	prepandemic MH	prior mental ill health	0.31	0.16	0.00	0.63	2579
**ALSPAC-G0**	Psych distress	2	Live Alone	sex	female	0.10	0.16	-0.22	0.42	2746
**ALSPAC-G0**	Psych distress	2	Live Alone	prepandemic MH	prior mental ill health	0.48	0.17	0.14	0.82	2279
**ALSPAC-G0**	Psych distress	3	Live Alone	sex	female	0.07	0.16	-0.25	0.38	2808
**ALSPAC-G0**	Psych distress	3	Live Alone	prepandemic MH	prior mental ill health	0.40	0.17	0.07	0.73	2349
**ALSPAC-G0**	Psych distress	0	Live Alone	age group	45 - 54	-0.02	0.26	-0.52	0.49	2702
**ALSPAC-G0**	Psych distress	0	Live Alone	age group	65 - 74	-0.61	0.19	-0.99	-0.24	2702
**ALSPAC-G0**	Psych distress	0	Live Alone	age group	75+	0.70	1.02	-1.30	2.70	2702
**ALSPAC-G0**	Psych distress	1	Live Alone	age group	45 - 54	-0.14	0.23	-0.59	0.30	3177
**ALSPAC-G0**	Psych distress	1	Live Alone	age group	65 - 74	-0.08	0.18	-0.44	0.27	3177
**ALSPAC-G0**	Psych distress	1	Live Alone	age group	75+	-0.81	1.02	-2.80	1.18	3177
**ALSPAC-G0**	Psych distress	2	Live Alone	age group	45 - 54	0.03	0.24	-0.44	0.50	2746
**ALSPAC-G0**	Psych distress	2	Live Alone	age group	65 - 74	-0.11	0.19	-0.48	0.26	2746
**ALSPAC-G0**	Psych distress	2	Live Alone	age group	75+	-0.44	1.02	-2.43	1.56	2746
**ALSPAC-G0**	Psych distress	3	Live Alone	age group	45 - 54	0.15	0.27	-0.38	0.69	2808
**ALSPAC-G0**	Psych distress	3	Live Alone	age group	65 - 74	0.02	0.17	-0.31	0.35	2808
**ALSPAC-G0**	Psych distress	3	Live Alone	age group	75+	-0.14	1.01	-2.13	1.85	2808

Note: TP = timepoint; coef = coefficient; se = standard error ; l_ci = lower 95% confidence interval ; u_ci = cupper 95% confidence interval. Models adjust for covariates.

Table S6. Regression coefficients from stratified cross-sectional models with continuous outcomes

cohort	outcome (Z)	TP	stratifier	stratified group	coef	coef_se	lower_ci	upper_ci	n
**NCDS**	Psych distress	T0	Loneliness at T0	not lonely	0.09	0.06	-0.03	0.21	3290
**NCDS**	Psych distress	T0	Loneliness at T0	lonely	-0.16	0.25	-0.65	0.32	158
**NCDS**	Psych distress	T1	Loneliness at T1	not lonely	-0.03	0.04	-0.12	0.05	3382
**NCDS**	Psych distress	T1	Loneliness at T1	lonely	-0.47	0.22	-0.91	-0.03	165
**NCDS**	Psych distress	T2	Loneliness at T1	not lonely	-0.03	0.04	-0.11	0.05	3411
**NCDS**	Psych distress	T2	Loneliness at T1	lonely	-0.52	0.20	-0.92	-0.12	170
**NCDS**	Psych distress	T3	Loneliness at T1	not lonely	-0.06	0.04	-0.13	0.02	3260
**NCDS**	Psych distress	T3	Loneliness at T1	lonely	-0.55	0.21	-0.97	-0.12	154
**NCDS**	Psych distress	T0	Sex	male	0.24	0.09	0.07	0.41	1681
**NCDS**	Psych distress	T0	Sex	female	0.00	0.08	-0.15	0.15	1870
**NCDS**	Psych distress	T1	Sex	male	0.07	0.06	-0.06	0.19	1714
**NCDS**	Psych distress	T1	Sex	female	0.07	0.07	-0.06	0.21	1853
**NCDS**	Psych distress	T2	Sex	male	0.08	0.06	-0.03	0.19	1768
**NCDS**	Psych distress	T2	Sex	female	0.01	0.06	-0.11	0.13	1924
**NCDS**	Psych distress	T3	Sex	male	0.03	0.05	-0.08	0.13	1662
**NCDS**	Psych distress	T3	Sex	female	-0.01	0.06	-0.12	0.11	1850
**NCDS**	Psych distress	T0	Shielding status at T1	not shielding	0.11	0.06	-0.01	0.23	3320
**NCDS**	Psych distress	T0	Shielding status at T1	shielding	-0.09	0.20	-0.49	0.31	207
**NCDS**	Psych distress	T1	Shielding status at T1	not shielding	0.07	0.05	-0.03	0.16	3345
**NCDS**	Psych distress	T1	Shielding status at T1	shielding	-0.03	0.19	-0.41	0.35	200
**NCDS**	Psych distress	T2	Shielding status at T1	not shielding	0.03	0.04	-0.05	0.12	3456
**NCDS**	Psych distress	T2	Shielding status at T1	shielding	0.07	0.18	-0.28	0.41	211
**NCDS**	Psych distress	T3	Shielding status at T1	not shielding	0.00	0.04	-0.08	0.08	3286
**NCDS**	Psych distress	T3	Shielding status at T1	shielding	-0.03	0.20	-0.42	0.35	203
**NCDS**	Psych distress	T0	Prior mental ill-health	no prior MH	0.05	0.04	-0.02	0.12	3175
**NCDS**	Psych distress	T0	Prior mental ill-health	prior MH	-0.14	0.09	-0.33	0.04	376
**NCDS**	Psych distress	T1	Prior mental ill-health	no prior MH	0.02	0.04	-0.06	0.10	3197
**NCDS**	Psych distress	T1	Prior mental ill-health	prior MH	0.04	0.18	-0.31	0.40	370
**NCDS**	Psych distress	T2	Prior mental ill-health	no prior MH	0.03	0.04	-0.04	0.11	3299
**NCDS**	Psych distress	T2	Prior mental ill-health	prior MH	-0.20	0.15	-0.49	0.10	393
**NCDS**	Psych distress	T3	Prior mental ill-health	no prior MH	-0.02	0.04	-0.09	0.06	3141

**NCDS**	Psych distress	T3	Prior mental ill-health	prior MH	-0.22	0.15	-0.51	0.08	371
**NCDS**	Life satisfaction	T0	Loneliness at T0	not lonely	-0.49	0.06	-0.61	-0.38	3290
**NCDS**	Life satisfaction	T0	Loneliness at T0	lonely	-0.24	0.21	-0.65	0.17	158
**NCDS**	Life satisfaction	T1	Loneliness at T1	not lonely	-0.18	0.05	-0.28	-0.08	3425
**NCDS**	Life satisfaction	T1	Loneliness at T1	lonely	0.01	0.20	-0.39	0.41	170
**NCDS**	Life satisfaction	T2	Loneliness at T1	not lonely	-0.20	0.04	-0.28	-0.12	3415
**NCDS**	Life satisfaction	T2	Loneliness at T1	lonely	0.05	0.20	-0.35	0.46	170
**NCDS**	Life satisfaction	T3	Loneliness at T1	not lonely	-0.17	0.05	-0.26	-0.09	3265
**NCDS**	Life satisfaction	T3	Loneliness at T1	lonely	-0.01	0.22	-0.44	0.43	155
**NCDS**	Life satisfaction	T0	Sex	male	-0.51	0.08	-0.67	-0.35	1681
**NCDS**	Life satisfaction	T0	Sex	female	-0.49	0.07	-0.63	-0.35	1870
**NCDS**	Life satisfaction	T1	Sex	male	-0.35	0.08	-0.50	-0.19	1739
**NCDS**	Life satisfaction	T1	Sex	female	-0.29	0.08	-0.44	-0.14	1884
**NCDS**	Life satisfaction	T2	Sex	male	-0.26	0.06	-0.39	-0.14	1770
**NCDS**	Life satisfaction	T2	Sex	female	-0.33	0.06	-0.44	-0.22	1926
**NCDS**	Life satisfaction	T3	Sex	male	-0.19	0.06	-0.31	-0.06	1665
**NCDS**	Life satisfaction	T3	Sex	female	-0.32	0.06	-0.45	-0.19	1855
**NCDS**	Life satisfaction	T0	Shielding status at T1	not shielding	-0.50	0.06	-0.61	-0.39	3320
**NCDS**	Life satisfaction	T0	Shielding status at T1	shielding	-0.44	0.23	-0.91	0.02	207
**NCDS**	Life satisfaction	T1	Shielding status at T1	not shielding	-0.33	0.06	-0.44	-0.22	3394
**NCDS**	Life satisfaction	T1	Shielding status at T1	shielding	-0.21	0.21	-0.62	0.20	206
**NCDS**	Life satisfaction	T2	Shielding status at T1	not shielding	-0.30	0.04	-0.38	-0.21	3459
**NCDS**	Life satisfaction	T2	Shielding status at T1	shielding	-0.46	0.20	-0.84	-0.07	212
**NCDS**	Life satisfaction	T3	Shielding status at T1	not shielding	-0.25	0.05	-0.35	-0.16	3293
**NCDS**	Life satisfaction	T3	Shielding status at T1	shielding	-0.36	0.20	-0.76	0.04	203
**NCDS**	Life satisfaction	T0	Prior mental ill-health	no prior MH	-0.46	0.06	-0.57	-0.35	3175
**NCDS**	Life satisfaction	T0	Prior mental ill-health	prior MH	-0.56	0.14	-0.84	-0.28	376
**NCDS**	Life satisfaction	T1	Prior mental ill-health	no prior MH	-0.28	0.05	-0.38	-0.18	3239
**NCDS**	Life satisfaction	T1	Prior mental ill-health	prior MH	-0.38	0.17	-0.71	-0.04	384
**NCDS**	Life satisfaction	T2	Prior mental ill-health	no prior MH	-0.31	0.04	-0.40	-0.22	3301
**NCDS**	Life satisfaction	T2	Prior mental ill-health	prior MH	-0.14	0.14	-0.41	0.12	395
**NCDS**	Life satisfaction	T3	Prior mental ill-health	no prior MH	-0.24	0.05	-0.34	-0.15	3148
**NCDS**	Life satisfaction	T3	Prior mental ill-health	prior MH	-0.20	0.14	-0.47	0.07	372

**NSHD**	Psych distress	T0	Loneliness at T0	not lonely	-0.12	0.14	-0.40	0.16	1113
**NSHD**	Psych distress	T0	Loneliness at T0	lonely	-0.49	0.27	-1.02	0.03	187
**NSHD**	Psych distress	T1	Loneliness at T1	not lonely	0.34	0.17	0.01	0.66	1238
**NSHD**	Psych distress	T1	Loneliness at T1	lonely	-0.68	0.37	-1.41	0.05	202
**NSHD**	Psych distress	T2	Loneliness at T1	not lonely	0.35	0.16	0.04	0.67	1242
**NSHD**	Psych distress	T2	Loneliness at T1	lonely	-0.53	0.31	-1.14	0.08	204
**NSHD**	Psych distress	T3	Loneliness at T1	not lonely	0.06	0.17	-0.28	0.40	864
**NSHD**	Psych distress	T3	Loneliness at T1	lonely	-0.61	0.34	-1.28	0.07	190
**NSHD**	Psych distress	T0	Sex	male	-0.16	0.19	-0.53	0.22	590
**NSHD**	Psych distress	T0	Sex	female	-0.02	0.16	-0.33	0.29	710
**NSHD**	Psych distress	T1	Sex	male	-0.03	0.23	-0.48	0.43	645
**NSHD**	Psych distress	T1	Sex	female	0.66	0.19	0.28	1.03	795
**NSHD**	Psych distress	T2	Sex	male	0.05	0.22	-0.39	0.49	649
**NSHD**	Psych distress	T2	Sex	female	0.46	0.17	0.12	0.80	797
**NSHD**	Psych distress	T3	Sex	male	-0.30	0.23	-0.75	0.15	493
**NSHD**	Psych distress	T3	Sex	female	0.48	0.19	0.10	0.86	561
**NSHD**	Psych distress	T0	Shielding status at T1	not shielding	-0.08	0.13	-0.33	0.17	1161
**NSHD**	Psych distress	T0	Shielding status at T1	shielding	0.14	0.37	-0.58	0.87	139
**NSHD**	Psych distress	T1	Shielding status at T1	not shielding	0.35	0.15	0.05	0.66	1285
**NSHD**	Psych distress	T1	Shielding status at T1	shielding	0.94	0.44	0.07	1.82	155
**NSHD**	Psych distress	T2	Shielding status at T1	not shielding	0.38	0.14	0.10	0.66	1286
**NSHD**	Psych distress	T2	Shielding status at T1	shielding	-0.04	0.44	-0.92	0.83	160
**NSHD**	Psych distress	T3	Shielding status at T1	not shielding	0.21	0.15	-0.10	0.51	964
**NSHD**	Psych distress	T3	Shielding status at T1	shielding	0.39	0.53	-0.66	1.44	90
**NSHD**	Psych distress	T0	Prior mental ill-health	no prior MH	-0.03	0.07	-0.18	0.11	1083
**NSHD**	Psych distress	T0	Prior mental ill-health	prior MH	-0.30	0.31	-0.91	0.30	217
**NSHD**	Psych distress	T1	Prior mental ill-health	no prior MH	0.31	0.16	-0.01	0.63	1074
**NSHD**	Psych distress	T1	Prior mental ill-health	prior MH	1.27	0.40	0.49	2.05	215
**NSHD**	Psych distress	T2	Prior mental ill-health	no prior MH	0.28	0.15	-0.01	0.57	1074
**NSHD**	Psych distress	T2	Prior mental ill-health	prior MH	0.39	0.37	-0.33	1.11	216
**NSHD**	Psych distress	T3	Prior mental ill-health	no prior MH	0.12	0.15	-0.18	0.42	820
**NSHD**	Psych distress	T3	Prior mental ill-health	prior MH	0.29	0.43	-0.57	1.15	162
**GS**	Life satisfaction	1	Prior MH	not shielding	-0.33	0.06	-0.44	-0.22	2457

**GS**	Life satisfaction	1	Prior MH	shielding	-0.37	0.13	-0.62	-0.13	525
**GS**	Life satisfaction	2	Prior MH	not shielding	-0.27	0.06	-0.38	-0.16	1811
**GS**	Life satisfaction	2	Prior MH	shielding	-0.22	0.14	-0.49	0.04	373
**GS**	Life satisfaction	3	Prior MH	not shielding	-0.26	0.07	-0.39	-0.14	1684
**GS**	Life satisfaction	3	Prior MH	shielding	-0.32	0.14	-0.62	-0.02	340
**GS**	Life satisfaction	1	sex	male	-0.33	0.09	-0.51	-0.16	1146
**GS**	Life satisfaction	1	sex	female	-0.32	0.07	-0.44	-0.19	1836
**GS**	Life satisfaction	2	sex	male	-0.27	0.10	-0.46	-0.09	822
**GS**	Life satisfaction	2	sex	female	-0.25	0.07	-0.38	-0.12	1362
**GS**	Life satisfaction	3	sex	male	-0.29	0.11	-0.49	-0.08	768
**GS**	Life satisfaction	3	sex	female	-0.23	0.08	-0.38	-0.08	1256
**GS**	Life satisfaction	1	Shielding	not shielding	-0.35	0.05	-0.46	-0.25	2874
**GS**	Life satisfaction	1	Shielding	shielding	-0.39	0.26	-0.93	0.15	108
**GS**	Life satisfaction	2	Shielding	not shielding	-0.27	0.06	-0.38	-0.17	2103
**GS**	Life satisfaction	2	Shielding	shielding	-0.41	0.28	-0.98	0.15	81
**GS**	Life satisfaction	3	Shielding	not shielding	-0.27	0.06	-0.39	-0.14	1951
**GS**	Life satisfaction	3	Shielding	shielding	-1.06	0.33	-1.67	-0.45	73
**GS**	Life satisfaction	1	T1 loneliness	not lonely	-0.24	0.05	-0.35	-0.14	2846
**GS**	Life satisfaction	1	T1 loneliness	lonely	0.12	0.17	-0.22	0.46	122
**GS**	Life satisfaction	2	T1 loneliness	not lonely	-0.20	0.06	-0.30	-0.09	2088
**GS**	Life satisfaction	2	T1 loneliness	lonely	0.08	0.27	-0.43	0.58	84
**GS**	Life satisfaction	3	T1 loneliness	not lonely	-0.24	0.07	-0.37	-0.12	1931
**GS**	Life satisfaction	3	T1 loneliness	lonely	0.27	0.28	-0.26	0.81	82
**GS**	Psych distress	0	Prior MH	not shielding	0.02	0.02	-0.02	0.06	2458
**GS**	Psych distress	0	Prior MH	shielding	0.20	0.08	0.05	0.34	526
**GS**	Psych distress	1	Prior MH	not shielding	0.13	0.06	0.03	0.23	2390
**GS**	Psych distress	1	Prior MH	shielding	0.20	0.18	-0.12	0.52	492
**GS**	Psych distress	2	Prior MH	not shielding	0.22	0.06	0.11	0.34	1747
**GS**	Psych distress	2	Prior MH	shielding	0.15	0.20	-0.22	0.52	359
**GS**	Psych distress	3	Prior MH	not shielding	0.16	0.07	0.03	0.30	1612
**GS**	Psych distress	3	Prior MH	shielding	0.12	0.21	-0.26	0.50	320
**GS**	Psych distress	0	sex	male	0.10	0.07	-0.01	0.22	1148
**GS**	Psych distress	0	sex	female	0.12	0.05	0.04	0.21	1836
**GS**	Psych distress	1	sex	male	0.24	0.12	0.06	0.42	1110

**GS**	Psych distress	1	sex	female	0.09	0.07	-0.04	0.22	1772
**GS**	Psych distress	2	sex	male	0.33	0.13	0.12	0.54	805
**GS**	Psych distress	2	sex	female	0.18	0.08	0.03	0.33	1301
**GS**	Psych distress	3	sex	male	0.39	0.14	0.16	0.61	742
**GS**	Psych distress	3	sex	female	0.08	0.09	-0.09	0.25	1190
**GS**	Psych distress	0	Shielding	not shielding	0.12	0.04	0.05	0.19	2876
**GS**	Psych distress	0	Shielding	shielding	0.16	0.30	-0.26	0.58	108
**GS**	Psych distress	1	Shielding	not shielding	0.16	0.06	0.05	0.27	2783
**GS**	Psych distress	1	Shielding	shielding	0.36	0.37	-0.32	1.04	99
**GS**	Psych distress	2	Shielding	not shielding	0.22	0.07	0.10	0.34	2026
**GS**	Psych distress	2	Shielding	shielding	0.90	0.42	0.24	1.56	80
**GS**	Psych distress	3	Shielding	not shielding	0.19	0.07	0.06	0.33	1864
**GS**	Psych distress	3	Shielding	shielding	0.84	0.58	-0.01	1.70	68
**GS**	Psych distress	0	T1 loneliness	not lonely	0.05	0.04	-0.02	0.12	2846
**GS**	Psych distress	0	T1 loneliness	lonely	0.12	0.19	-0.27	0.50	122
**GS**	Psych distress	1	T1 loneliness	not lonely	0.00	0.05	-0.11	0.10	2758
**GS**	Psych distress	1	T1 loneliness	lonely	-0.27	0.27	-0.83	0.30	113
**GS**	Psych distress	2	T1 loneliness	not lonely	0.11	0.06	-0.01	0.23	2018
**GS**	Psych distress	2	T1 loneliness	lonely	-0.52	0.34	-1.22	0.19	78
**GS**	Psych distress	3	T1 loneliness	not lonely	0.09	0.07	-0.04	0.23	1846
**GS**	Psych distress	3	T1 loneliness	lonely	-0.29	0.32	-0.95	0.36	77
**BCS**	Psych distress	T0	Loneliness at T0	not lonely	-0.02	0.07	-0.16	0.12	2266
**BCS**	Psych distress	T0	Loneliness at T0	lonely	-0.19	0.28	-0.75	0.36	139
**BCS**	Psych distress	T1	Loneliness at T1	not lonely	-0.11	0.06	-0.23	0.02	2456
**BCS**	Psych distress	T1	Loneliness at T1	lonely	-0.45	0.19	-0.83	-0.07	152
**BCS**	Psych distress	T2	Loneliness at T1	not lonely	0.00	0.07	-0.13	0.13	2459
**BCS**	Psych distress	T2	Loneliness at T1	lonely	-0.47	0.22	-0.91	-0.03	152
**BCS**	Psych distress	T3	Loneliness at T1	not lonely	0.06	0.06	-0.06	0.19	2221
**BCS**	Psych distress	T3	Loneliness at T1	lonely	-0.24	0.21	-0.66	0.18	135
**BCS**	Psych distress	T0	Sex	male	-0.12	0.09	-0.30	0.05	961
**BCS**	Psych distress	T0	Sex	female	0.14	0.11	-0.08	0.37	1500
**BCS**	Psych distress	T1	Sex	male	-0.07	0.10	-0.26	0.12	1054
**BCS**	Psych distress	T1	Sex	female	-0.02	0.10	-0.22	0.17	1562

**BCS**	Psych distress	T2	Sex	male	0.03	0.09	-0.15	0.21	1073
**BCS**	Psych distress	T2	Sex	female	0.05	0.09	-0.13	0.23	1600
**BCS**	Psych distress	T3	Sex	male	0.10	0.09	-0.08	0.27	938
**BCS**	Psych distress	T3	Sex	female	0.15	0.09	-0.02	0.32	1468
**BCS**	Psych distress	T0	Shielding status at T1	not shielding	0.01	0.07	-0.14	0.15	2333
**BCS**	Psych distress	T0	Shielding status at T1	shielding	-0.12	0.35	-0.82	0.59	122
**BCS**	Psych distress	T1	Shielding status at T1	not shielding	-0.02	0.07	-0.15	0.11	2483
**BCS**	Psych distress	T1	Shielding status at T1	shielding	-0.31	0.33	-0.96	0.34	126
**BCS**	Psych distress	T2	Shielding status at T1	not shielding	0.02	0.07	-0.11	0.15	2537
**BCS**	Psych distress	T2	Shielding status at T1	shielding	0.39	0.30	-0.20	0.97	129
**BCS**	Psych distress	T3	Shielding status at T1	not shielding	0.11	0.06	-0.01	0.24	2280
**BCS**	Psych distress	T3	Shielding status at T1	shielding	0.32	0.31	-0.29	0.93	121
**BCS**	Psych distress	T0	Prior mental ill-health	no prior MH	-0.02	0.04	-0.10	0.07	2095
**BCS**	Psych distress	T0	Prior mental ill-health	prior MH	-0.03	0.12	-0.26	0.20	366
**BCS**	Psych distress	T1	Prior mental ill-health	no prior MH	-0.04	0.06	-0.16	0.07	2216
**BCS**	Psych distress	T1	Prior mental ill-health	prior MH	-0.09	0.18	-0.45	0.28	400
**BCS**	Psych distress	T2	Prior mental ill-health	no prior MH	-0.07	0.06	-0.18	0.04	2266
**BCS**	Psych distress	T2	Prior mental ill-health	prior MH	0.29	0.16	-0.02	0.60	407
**BCS**	Psych distress	T3	Prior mental ill-health	no prior MH	0.04	0.05	-0.07	0.15	2049
**BCS**	Psych distress	T3	Prior mental ill-health	prior MH	0.27	0.16	-0.05	0.60	357
**BCS**	Life satisfaction	T0	Loneliness at T0	not lonely	-0.32	0.07	-0.45	-0.19	2264
**BCS**	Life satisfaction	T0	Loneliness at T0	lonely	-0.68	0.23	-1.15	-0.22	137
**BCS**	Life satisfaction	T1	Loneliness at T1	not lonely	-0.02	0.07	-0.16	0.12	2474
**BCS**	Life satisfaction	T1	Loneliness at T1	lonely	-0.01	0.21	-0.43	0.40	154
**BCS**	Life satisfaction	T2	Loneliness at T1	not lonely	-0.21	0.06	-0.33	-0.10	2463
**BCS**	Life satisfaction	T2	Loneliness at T1	lonely	-0.23	0.20	-0.63	0.16	153
**BCS**	Life satisfaction	T3	Loneliness at T1	not lonely	-0.10	0.07	-0.23	0.03	2226
**BCS**	Life satisfaction	T3	Loneliness at T1	lonely	0.07	0.21	-0.34	0.48	136
**BCS**	Life satisfaction	T0	Sex	male	-0.48	0.10	-0.67	-0.29	959
**BCS**	Life satisfaction	T0	Sex	female	-0.40	0.10	-0.60	-0.21	1498
**BCS**	Life satisfaction	T1	Sex	male	-0.13	0.13	-0.38	0.12	1059
**BCS**	Life satisfaction	T1	Sex	female	-0.18	0.09	-0.35	-0.01	1579
**BCS**	Life satisfaction	T2	Sex	male	-0.43	0.09	-0.61	-0.26	1077
**BCS**	Life satisfaction	T2	Sex	female	-0.25	0.08	-0.40	-0.09	1601

**BCS**	Life satisfaction	T3	Sex	male	-0.26	0.10	-0.46	-0.06	940
**BCS**	Life satisfaction	T3	Sex	female	-0.14	0.08	-0.30	0.03	1472
**BCS**	Life satisfaction	T0	Shielding status at T1	not shielding	-0.45	0.07	-0.59	-0.32	2329
**BCS**	Life satisfaction	T0	Shielding status at T1	shielding	-0.06	0.33	-0.71	0.58	122
**BCS**	Life satisfaction	T1	Shielding status at T1	not shielding	-0.18	0.08	-0.34	-0.01	2504
**BCS**	Life satisfaction	T1	Shielding status at T1	shielding	0.20	0.41	-0.60	1.01	127
**BCS**	Life satisfaction	T2	Shielding status at T1	not shielding	-0.31	0.06	-0.43	-0.19	2542
**BCS**	Life satisfaction	T2	Shielding status at T1	shielding	-0.41	0.30	-1.00	0.19	129
**BCS**	Life satisfaction	T3	Shielding status at T1	not shielding	-0.17	0.07	-0.30	-0.04	2286
**BCS**	Life satisfaction	T3	Shielding status at T1	shielding	-0.25	0.30	-0.83	0.34	121
**BCS**	Life satisfaction	T0	Prior mental ill-health	no prior MH	-0.38	0.07	-0.51	-0.25	2091
**BCS**	Life satisfaction	T0	Prior mental ill-health	prior MH	-0.63	0.19	-1.00	-0.25	366
**BCS**	Life satisfaction	T1	Prior mental ill-health	no prior MH	-0.15	0.08	-0.31	0.01	2234
**BCS**	Life satisfaction	T1	Prior mental ill-health	prior MH	-0.21	0.20	-0.61	0.19	404
**BCS**	Life satisfaction	T2	Prior mental ill-health	no prior MH	-0.28	0.06	-0.41	-0.16	2270
**BCS**	Life satisfaction	T2	Prior mental ill-health	prior MH	-0.37	0.16	-0.68	-0.06	408
**BCS**	Life satisfaction	T3	Prior mental ill-health	no prior MH	-0.19	0.07	-0.32	-0.05	2054
**BCS**	Life satisfaction	T3	Prior mental ill-health	prior MH	-0.09	0.17	-0.42	0.24	358
**NS**	Psych distress	T0	Loneliness at T0	not lonely	0.04	0.08	-0.11	0.19	968
**NS**	Psych distress	T0	Loneliness at T0	lonely	-0.05	0.31	-0.67	0.58	112
**NS**	Psych distress	T1	Loneliness at T1	not lonely	0.27	0.12	0.04	0.49	1017
**NS**	Psych distress	T1	Loneliness at T1	lonely	0.17	0.31	-0.45	0.79	118
**NS**	Psych distress	T2	Loneliness at T1	not lonely	0.23	0.10	0.03	0.43	1023
**NS**	Psych distress	T2	Loneliness at T1	lonely	-0.37	0.24	-0.84	0.10	118
**NS**	Psych distress	T3	Loneliness at T1	not lonely	0.21	0.10	0.03	0.40	961
**NS**	Psych distress	T3	Loneliness at T1	lonely	-0.49	0.25	-0.98	0.00	110
**NS**	Psych distress	T0	Sex	male	0.27	0.11	0.05	0.50	380
**NS**	Psych distress	T0	Sex	female	-0.11	0.10	-0.31	0.09	734
**NS**	Psych distress	T1	Sex	male	0.31	0.17	-0.02	0.64	389
**NS**	Psych distress	T1	Sex	female	0.43	0.17	0.10	0.76	746
**NS**	Psych distress	T2	Sex	male	0.23	0.14	-0.03	0.50	401
**NS**	Psych distress	T2	Sex	female	0.12	0.12	-0.12	0.37	781
**NS**	Psych distress	T3	Sex	male	0.30	0.14	0.03	0.58	379

McElroy E, *et al. BMJ Ment Health* 2023; 26:e300842. doi: 10.1136/bmjment-2023-300842

**NS**	Psych distress	T3	Sex	female	0.04	0.11	-0.18	0.27	724
**NS**	Psych distress	T0	Shielding status at T1	not shielding	0.07	0.08	-0.08	0.23	1078
**NS**	Psych distress	T0	Shielding status at T1	shielding	-0.66	0.40	-1.51	0.19	32
**NS**	Psych distress	T1	Shielding status at T1	not shielding	0.36	0.14	0.09	0.62	1099
**NS**	Psych distress	T1	Shielding status at T1	shielding	0.43	0.34	-0.28	1.15	32
**NS**	Psych distress	T2	Shielding status at T1	not shielding	0.17	0.10	-0.02	0.36	1144
**NS**	Psych distress	T2	Shielding status at T1	shielding	-0.04	0.36	-0.79	0.72	34
**NS**	Psych distress	T3	Shielding status at T1	not shielding	0.15	0.09	-0.03	0.33	
**NS**	Psych distress	T3	Shielding status at T1	shielding	-0.17	0.62	-1.49	1.15	31
**NS**	Psych distress	T0	Prior mental ill-health	no prior MH	0.00	0.05	-0.10	0.11	849
**NS**	Psych distress	T0	Prior mental ill-health	prior MH	0.12	0.13	-0.14	0.37	265
**NS**	Psych distress	T1	Prior mental ill-health	no prior MH	0.29	0.17	-0.04	0.62	864
**NS**	Psych distress	T1	Prior mental ill-health	prior MH	0.23	0.22	-0.22	0.67	271
**NS**	Psych distress	T2	Prior mental ill-health	no prior MH	0.12	0.11	-0.10	0.34	902
**NS**	Psych distress	T2	Prior mental ill-health	prior MH	0.17	0.20	-0.22	0.55	280
**NS**	Psych distress	T3	Prior mental ill-health	no prior MH	0.15	0.09	-0.03	0.33	841
**NS**	Psych distress	T3	Prior mental ill-health	prior MH	0.10	0.20	-0.30	0.50	262
**NS**	Life satisfaction	T0	Loneliness at T0	not lonely	0.04	0.02	0.00	0.09	968
**NS**	Life satisfaction	T0	Loneliness at T0	lonely	0.01	0.08	-0.14	0.16	112
**NS**	Life satisfaction	T1	Loneliness at T1	not lonely	-0.16	0.08	-0.32	0.00	1037
**NS**	Life satisfaction	T1	Loneliness at T1	lonely	-0.34	0.20	-0.73	0.06	120
**NS**	Life satisfaction	T2	Loneliness at T1	not lonely	-0.10	0.06	-0.23	0.02	1027
**NS**	Life satisfaction	T2	Loneliness at T1	lonely	0.03	0.15	-0.28	0.34	118
**NS**	Life satisfaction	T3	Loneliness at T1	not lonely	-0.06	0.06	-0.19	0.06	964
**NS**	Life satisfaction	T3	Loneliness at T1	lonely	0.28	0.16	-0.04	0.60	110
**NS**	Life satisfaction	T0	Sex	male	0.09	0.03	0.02	0.15	380
**NS**	Life satisfaction	T0	Sex	female	0.02	0.03	-0.04	0.07	734
**NS**	Life satisfaction	T1	Sex	male	-0.28	0.11	-0.49	-0.08	396
**NS**	Life satisfaction	T1	Sex	female	-0.25	0.17	-0.58	0.08	762
**NS**	Life satisfaction	T2	Sex	male	-0.09	0.08	-0.25	0.08	404
**NS**	Life satisfaction	T2	Sex	female	-0.13	0.08	-0.29	0.03	783
**NS**	Life satisfaction	T3	Sex	male	-0.18	0.09	-0.37	0.00	379
**NS**	Life satisfaction	T3	Sex	female	0.05	0.08	-0.10	0.20	727
**NS**	Life satisfaction	T0	Shielding status at T1	not shielding	0.05	0.02	0.01	0.10	1078

**NS**	Life satisfaction	T0	Shielding status at T1	shielding	-0.13	0.09	-0.32	0.05	32
**NS**	Life satisfaction	T1	Shielding status at T1	not shielding	-0.26	0.11	-0.48	-0.03	1122
**NS**	Life satisfaction	T1	Shielding status at T1	shielding	-0.31	0.28	-0.89	0.28	32
**NS**	Life satisfaction	T2	Shielding status at T1	not shielding	-0.12	0.06	-0.25	0.00	1149
**NS**	Life satisfaction	T2	Shielding status at T1	shielding	0.23	0.25	-0.30	0.76	34
**NS**	Life satisfaction	T3	Shielding status at T1	not shielding	-0.06	0.06	-0.18	0.07	1071
**NS**	Life satisfaction	T3	Shielding status at T1	shielding	-0.17	0.29	-0.80	0.46	31
**NS**	Life satisfaction	T0	Prior mental ill-health	no prior MH	0.03	0.02	-0.01	0.08	849
**NS**	Life satisfaction	T0	Prior mental ill-health	prior MH	0.08	0.05	-0.01	0.17	265
**NS**	Life satisfaction	T1	Prior mental ill-health	no prior MH	-0.33	0.13	-0.58	-0.08	879
**NS**	Life satisfaction	T1	Prior mental ill-health	prior MH	-0.03	0.18	-0.39	0.34	279
**NS**	Life satisfaction	T2	Prior mental ill-health	no prior MH	-0.10	0.07	-0.25	0.04	904
**NS**	Life satisfaction	T2	Prior mental ill-health	prior MH	-0.10	0.13	-0.35	0.15	283
**NS**	Life satisfaction	T3	Prior mental ill-health	no prior MH	-0.02	0.07	-0.16	0.11	844
**NS**	Life satisfaction	T3	Prior mental ill-health	prior MH	-0.08	0.13	-0.34	0.18	262
**ELSA**	Psych distress	T0	Loneliness at T0	not lonely	0.07	0.04	0.00	0.15	4837
**ELSA**	Psych distress	T0	Loneliness at T0	lonely	-0.19	0.17	-0.52	0.14	280
**ELSA**	Psych distress	T1	Loneliness at T1	not lonely	0.13	0.04	0.04	0.22	5161
**ELSA**	Psych distress	T1	Loneliness at T1	lonely	0.09	0.12	-0.14	0.31	304
**ELSA**	Psych distress	T3	Loneliness at T1	not lonely	0.10	0.04	0.01	0.18	4771
**ELSA**	Psych distress	T3	Loneliness at T1	lonely	0.30	0.13	0.05	0.56	271
**ELSA**	Psych distress	T0	Sex	male	0.16	0.07	0.03	0.29	2336
**ELSA**	Psych distress	T0	Sex	female	0.23	0.05	0.12	0.34	3135
**ELSA**	Psych distress	T1	Sex	male	0.25	0.08	0.10	0.40	2335
**ELSA**	Psych distress	T1	Sex	female	0.29	0.05	0.19	0.40	3135
**ELSA**	Psych distress	T3	Sex	male	0.28	0.07	0.14	0.42	2164
**ELSA**	Psych distress	T3	Sex	female	0.20	0.05	0.09	0.30	2882
**ELSA**	Psych distress	T0	Shielding status at T1	not shielding	0.22	0.05	0.13	0.31	4543
**ELSA**	Psych distress	T0	Shielding status at T1	shielding	0.09	0.10	-0.11	0.29	921
**ELSA**	Psych distress	T1	Shielding status at T1	not shielding	0.28	0.05	0.18	0.37	4542
**ELSA**	Psych distress	T1	Shielding status at T1	shielding	0.18	0.10	-0.01	0.38	921
**ELSA**	Psych distress	T3	Shielding status at T1	not shielding	0.22	0.05	0.12	0.31	4240
**ELSA**	Psych distress	T3	Shielding status at T1	shielding	0.26	0.10	0.07	0.45	800

**ELSA**	Psych distress	T1	Prior mental ill-health	good MH	0.20	0.05	0.11	0.29	4880
**ELSA**	Psych distress	T1	Prior mental ill-health	ill health	0.22	0.11	0.01	0.44	590
**ELSA**	Psych distress	T3	Prior mental ill-health	good MH	0.15	0.04	0.07	0.24	4528
**ELSA**	Psych distress	T3	Prior mental ill-health	ill health	0.18	0.11	-0.03	0.40	518
**ELSA**	Life satisfaction	T0	Loneliness at T0	not lonely	-0.23	0.04	-0.31	-0.14	4790
**ELSA**	Life satisfaction	T0	Loneliness at T0	lonely	-0.08	0.16	-0.39	0.23	268
**ELSA**	Life satisfaction	T1	Loneliness at T1	not lonely	-0.04	0.04	-0.12	0.04	5145
**ELSA**	Life satisfaction	T1	Loneliness at T1	lonely	0.16	0.17	-0.16	0.49	303
**ELSA**	Life satisfaction	T3	Loneliness at T1	not lonely	-0.12	0.05	-0.20	-0.03	4764
**ELSA**	Life satisfaction	T3	Loneliness at T1	lonely	-0.18	0.18	-0.54	0.17	270
**ELSA**	Life satisfaction	T0	Sex	male	-0.30	0.07	-0.44	-0.16	2193
**ELSA**	Life satisfaction	T0	Sex	female	-0.33	0.05	-0.43	-0.23	2897
**ELSA**	Life satisfaction	T1	Sex	male	-0.11	0.07	-0.25	0.04	2333
**ELSA**	Life satisfaction	T1	Sex	female	-0.14	0.05	-0.25	-0.04	3120
**ELSA**	Life satisfaction	T3	Sex	male	-0.20	0.08	-0.36	-0.05	2161
**ELSA**	Life satisfaction	T3	Sex	female	-0.21	0.05	-0.31	-0.10	2877
**ELSA**	Life satisfaction	T0	Shielding status at T1	not shielding	-0.33	0.05	-0.42	-0.24	4261
**ELSA**	Life satisfaction	T0	Shielding status at T1	shielding	-0.31	0.11	-0.52	-0.09	824
**ELSA**	Life satisfaction	T1	Shielding status at T1	not shielding	-0.13	0.05	-0.22	-0.04	4528
**ELSA**	Life satisfaction	T1	Shielding status at T1	shielding	-0.08	0.11	-0.29	0.13	918
**ELSA**	Life satisfaction	T3	Shielding status at T1	not shielding	-0.21	0.05	-0.31	-0.11	4233
**ELSA**	Life satisfaction	T3	Shielding status at T1	shielding	-0.21	0.10	-0.40	-0.01	798
**ELSA**	Life satisfaction	T0	Prior mental ill-health	good MH	-0.27	0.04	-0.35	-0.19	4589
**ELSA**	Life satisfaction	T0	Prior mental ill-health	ill health	-0.13	0.13	-0.38	0.13	501
**ELSA**	Life satisfaction	T1	Prior mental ill-health	good MH	-0.10	0.05	-0.19	-0.01	4866
**ELSA**	Life satisfaction	T1	Prior mental ill-health	ill health	0.04	0.11	-0.18	0.27	587
**ELSA**	Life satisfaction	T3	Prior mental ill-health	good MH	-0.18	0.05	-0.27	-0.08	4523
**ELSA**	Life satisfaction	T3	Prior mental ill-health	ill health	-0.09	0.13	-0.33	0.16	515
**USOC**	Psych distress	T0	Shielding status at T1	not shielding	0.06	0.03	-0.01	0.13	12200
**USOC**	Psych distress	T0	Shielding status at T1	shielding	0.19	0.12	-0.04	0.42	784
**USOC**	Life satisfaction	T0	Shielding status at T1	not shielding	-0.10	0.03	-0.16	-0.03	12200
**USOC**	Life satisfaction	T0	Shielding status at T1	shielding	-0.11	0.11	-0.33	0.12	784
**USOC**	Life satisfaction	T1	Shielding status at T1	not shielding	-0.25	0.04	-0.33	-0.17	9530

**USOC**	Life satisfaction	T1	Shielding status at T1	shielding	-0.32	0.14	-0.59	-0.04	602
**USOC**	Psych distress	T0	Loneliness at T0	not lonely	-0.03	0.02	-0.07	0.01	20541
**USOC**	Psych distress	T0	Loneliness at T0	lonely	-0.13	0.10	-0.33	0.06	1646
**USOC**	Life satisfaction	T0	Loneliness at T0	not lonely	-0.01	0.02	-0.05	0.04	20541
**USOC**	Life satisfaction	T0	Loneliness at T0	lonely	-0.03	0.08	-0.20	0.13	1646
**USOC**	Life satisfaction	T1	Loneliness at T1	not lonely	-0.18	0.04	-0.27	-0.10	9470
**USOC**	Life satisfaction	T1	Loneliness at T1	lonely	-0.05	0.11	-0.28	0.17	662
**USOC**	Psych distress	T0	Sex	male	0.03	0.03	-0.03	0.10	9917
**USOC**	Psych distress	T0	Sex	female	0.02	0.03	-0.04	0.08	12270
**USOC**	Life satisfaction	T0	Sex	male	-0.09	0.03	-0.16	-0.02	9917
**USOC**	Life satisfaction	T0	Sex	female	-0.04	0.03	-0.10	0.02	12270
**USOC**	Life satisfaction	T1	Sex	male	-0.32	0.07	-0.45	-0.18	4210
**USOC**	Life satisfaction	T1	Sex	female	-0.22	0.05	-0.31	-0.12	5922
**USOC**	Life satisfaction	T1	Prior mental ill-health	good MH	-0.22	0.05	-0.31	-0.13	8341
**USOC**	Life satisfaction	T1	Prior mental ill-health	ill health	-0.33	0.08	-0.49	-0.17	1791
**USOC**	Psych distress	T0	Age	16-24	0.28	0.14	0.00	0.55	2615
**USOC**	Psych distress	T0	Age	25-34	0.01	0.08	-0.16	0.17	2522
**USOC**	Psych distress	T0	Age	35-44	0.00	0.09	-0.17	0.18	3227
**USOC**	Psych distress	T0	Age	45-54	0.02	0.06	-0.10	0.14	4088
**USOC**	Psych distress	T0	Age	55-64	0.13	0.05	0.02	0.23	3978
**USOC**	Psych distress	T0	Age	65-74	0.03	0.04	-0.04	0.10	3503
**USOC**	Psych distress	T0	Age	75+	-0.07	0.04	-0.14	0.00	2254
**USOC**	Life satisfaction	T0	Age	16-24	-0.12	0.13	-0.36	0.13	2615
**USOC**	Life satisfaction	T0	Age	25-34	-0.05	0.07	-0.18	0.09	2522
**USOC**	Life satisfaction	T0	Age	35-44	-0.12	0.09	-0.29	0.04	3227
**USOC**	Life satisfaction	T0	Age	45-54	-0.05	0.06	-0.18	0.07	4088
**USOC**	Life satisfaction	T0	Age	55-64	-0.14	0.05	-0.24	-0.04	3978
**USOC**	Life satisfaction	T0	Age	65-74	-0.09	0.04	-0.18	-0.01	3503
**USOC**	Life satisfaction	T0	Age	75+	0.04	0.05	-0.06	0.13	2254
**USOC**	Life satisfaction	T1	Age	16-24	-0.05	0.37	-0.77	0.67	651
**USOC**	Life satisfaction	T1	Age	25-34	-0.56	0.16	-0.88	-0.24	935
**USOC**	Life satisfaction	T1	Age	35-44	-0.50	0.14	-0.78	-0.22	1506
**USOC**	Life satisfaction	T1	Age	45-54	-0.11	0.11	-0.32	0.10	2069
**USOC**	Life satisfaction	T1	Age	55-64	-0.27	0.07	-0.42	-0.13	2255

**USOC**	Life satisfaction	T1	Age	65-74	-0.28	0.07	-0.42	-0.14	1986
**USOC**	Life satisfaction	T1	Age	75+	-0.12	0.11	-0.33	0.09	1524
**USOC**	Psych distress	T1	Shielding status at T1	not shielding	0.17	0.03	0.11	0.23	11364
**USOC**	Psych distress	T1	Shielding status at T1	shielding	0.23	0.11	0.01	0.44	724
**USOC**	Psych distress	T2	Shielding status at T1	not shielding	0.14	0.03	0.08	0.20	9309
**USOC**	Psych distress	T2	Shielding status at T1	shielding	0.25	0.12	0.01	0.50	596
**USOC**	Psych distress	T3	Shielding status at T1	not shielding	0.12	0.03	0.06	0.18	9304
**USOC**	Psych distress	T3	Shielding status at T1	shielding	0.10	0.12	-0.14	0.34	609
**USOC**	Life satisfaction	T2	Shielding status at T1	not shielding	-0.16	0.03	-0.22	-0.10	9309
**USOC**	Life satisfaction	T2	Shielding status at T1	shielding	-0.17	0.11	-0.39	0.04	596
**USOC**	Life satisfaction	T3	Shielding status at T1	not shielding	-0.19	0.03	-0.25	-0.13	9304
**USOC**	Life satisfaction	T3	Shielding status at T1	shielding	-0.28	0.10	-0.48	-0.08	609
**USOC**	Psych distress	T1	Loneliness at T1	not lonely	0.04	0.03	-0.01	0.09	11234
**USOC**	Psych distress	T1	Loneliness at T1	lonely	-0.06	0.10	-0.27	0.14	854
**USOC**	Psych distress	T2	Loneliness at T1	not lonely	0.05	0.03	-0.01	0.11	9258
**USOC**	Psych distress	T2	Loneliness at T1	lonely	-0.05	0.13	-0.30	0.20	647
**USOC**	Psych distress	T3	Loneliness at T1	not lonely	0.03	0.03	-0.03	0.09	9259
**USOC**	Psych distress	T3	Loneliness at T1	lonely	-0.20	0.12	-0.44	0.04	654
**USOC**	Life satisfaction	T2	Loneliness at T1	not lonely	-0.09	0.03	-0.15	-0.03	9258
**USOC**	Life satisfaction	T2	Loneliness at T1	lonely	-0.07	0.09	-0.24	0.10	647
**USOC**	Life satisfaction	T3	Loneliness at T1	not lonely	-0.14	0.03	-0.19	-0.08	9259
**USOC**	Life satisfaction	T3	Loneliness at T1	lonely	-0.02	0.09	-0.20	0.15	654
**USOC**	Psych distress	T1	Sex	male	0.21	0.05	0.12	0.30	5080
**USOC**	Psych distress	T1	Sex	female	0.14	0.04	0.07	0.22	7008
**USOC**	Psych distress	T2	Sex	male	0.20	0.05	0.10	0.30	4137
**USOC**	Psych distress	T2	Sex	female	0.10	0.04	0.02	0.18	5768
**USOC**	Psych distress	T3	Sex	male	0.14	0.05	0.04	0.24	4123
**USOC**	Psych distress	T3	Sex	female	0.11	0.04	0.03	0.19	5790
**USOC**	Life satisfaction	T2	Sex	male	-0.27	0.05	-0.37	-0.18	4137
**USOC**	Life satisfaction	T2	Sex	female	-0.09	0.04	-0.16	-0.02	5768
**USOC**	Life satisfaction	T3	Sex	male	-0.32	0.05	-0.41	-0.23	4123
**USOC**	Life satisfaction	T3	Sex	female	-0.11	0.03	-0.18	-0.05	5790
**USOC**	Psych distress	T1	Age	16-24	-0.23	0.36	-0.93	0.48	923
**USOC**	Psych distress	T1	Age	25-34	0.34	0.14	0.06	0.62	1223

McElroy E, et al. BMJ Ment Health 2023; 26:e300842. doi: 10.1136/bmjment-2023-300842

**USOC**	Psych distress	T1	Age	35-44	0.08	0.13	-0.17	0.34	1831
**USOC**	Psych distress	T1	Age	45-54	0.21	0.08	0.05	0.37	2496
**USOC**	Psych distress	T1	Age	55-64	0.18	0.06	0.07	0.29	2590
**USOC**	Psych distress	T1	Age	65-74	0.21	0.05	0.11	0.30	2183
**USOC**	Psych distress	T1	Age	75+	0.10	0.07	-0.03	0.24	842
**USOC**	Psych distress	T2	Age	16-24	-0.31	0.29	-0.88	0.26	590
**USOC**	Psych distress	T2	Age	25-34	0.48	0.17	0.15	0.80	897
**USOC**	Psych distress	T2	Age	35-44	0.24	0.14	-0.05	0.52	1421
**USOC**	Psych distress	T2	Age	45-54	0.17	0.09	0.00	0.34	1994
**USOC**	Psych distress	T2	Age	55-64	0.15	0.06	0.04	0.27	2281
**USOC**	Psych distress	T2	Age	65-74	0.13	0.05	0.03	0.24	1979
**USOC**	Psych distress	T2	Age	75+	-0.03	0.07	-0.16	0.11	743
**USOC**	Psych distress	T3	Age	16-24	-0.07	0.38	-0.83	0.68	599
**USOC**	Psych distress	T3	Age	25-34	0.17	0.15	-0.13	0.47	891
**USOC**	Psych distress	T3	Age	35-44	0.15	0.13	-0.10	0.40	1437
**USOC**	Psych distress	T3	Age	45-54	0.15	0.08	-0.01	0.31	2043
**USOC**	Psych distress	T3	Age	55-64	0.11	0.06	0.00	0.23	2281
**USOC**	Psych distress	T3	Age	65-74	0.14	0.05	0.04	0.25	1944
**USOC**	Psych distress	T3	Age	75+	0.07	0.07	-0.08	0.21	718
**USOC**	Life satisfaction	T2	Age	16-24	0.07	0.19	-0.31	0.44	590
**USOC**	Life satisfaction	T2	Age	25-34	-0.38	0.11	-0.60	-0.16	897
**USOC**	Life satisfaction	T2	Age	35-44	-0.44	0.11	-0.66	-0.23	1421
**USOC**	Life satisfaction	T2	Age	45-54	-0.19	0.08	-0.35	-0.04	1994
**USOC**	Life satisfaction	T2	Age	55-64	-0.25	0.06	-0.37	-0.14	2281
**USOC**	Life satisfaction	T2	Age	65-74	-0.10	0.06	-0.21	0.02	1979
**USOC**	Life satisfaction	T2	Age	75+	0.08	0.08	-0.09	0.24	743
**USOC**	Life satisfaction	T3	Age	16-24	0.22	0.21	-0.20	0.63	599
**USOC**	Life satisfaction	T3	Age	25-34	-0.22	0.11	-0.43	-0.01	891
**USOC**	Life satisfaction	T3	Age	35-44	-0.26	0.10	-0.45	-0.06	1437
**USOC**	Life satisfaction	T3	Age	45-54	-0.19	0.07	-0.32	-0.05	2043
**USOC**	Life satisfaction	T3	Age	55-64	-0.26	0.06	-0.37	-0.16	2281
**USOC**	Life satisfaction	T3	Age	65-74	-0.16	0.05	-0.27	-0.06	1944
**USOC**	Life satisfaction	T3	Age	75+	-0.17	0.08	-0.32	-0.01	718
**USOC**	Psych distress	T1	Prior mental ill-health	good MH	0.13	0.03	0.07	0.18	9887

**USOC**	Psych distress	T1	Prior mental ill-health	ill health	0.21	0.08	0.05	0.37	2201
**USOC**	Psych distress	T2	Prior mental ill-health	good MH	0.09	0.03	0.04	0.15	8164
**USOC**	Psych distress	T2	Prior mental ill-health	ill health	0.22	0.09	0.04	0.39	1741
**USOC**	Psych distress	T3	Prior mental ill-health	good MH	0.08	0.03	0.02	0.14	8156
**USOC**	Psych distress	T3	Prior mental ill-health	ill health	0.10	0.09	-0.07	0.28	1757
**USOC**	Life satisfaction	T2	Prior mental ill-health	good MH	-0.10	0.03	-0.16	-0.04	8164
**USOC**	Life satisfaction	T2	Prior mental ill-health	ill health	-0.32	0.07	-0.45	-0.19	1741
**USOC**	Life satisfaction	T3	Prior mental ill-health	good MH	-0.15	0.03	-0.21	-0.09	8156
**USOC**	Life satisfaction	T3	Prior mental ill-health	ill health	-0.27	0.06	-0.39	-0.14	1757
**ALSPAC-G1**	Psych distress	T0	Sex	male	0.04	0.15	-0.25	0.32	501
**ALSPAC-G1**	Psych distress	T0	Sex	female	0.29	0.11	0.08	0.51	1308
**ALSPAC-G1**	Psych distress	T1	Sex	male	0.21	0.13	-0.04	0.46	586
**ALSPAC-G1**	Psych distress	T1	Sex	female	0.30	0.11	0.09	0.51	1478
**ALSPAC-G1**	Psych distress	T2	Sex	male	0.07	0.14	-0.20	0.34	459
**ALSPAC-G1**	Psych distress	T2	Sex	female	0.25	0.12	0.01	0.49	1151
**ALSPAC-G1**	Psych distress	T3	Sex	male	0.18	0.14	-0.10	0.47	539
**ALSPAC-G1**	Psych distress	T3	Sex	female	0.31	0.11	0.09	0.52	1380
**ALSPAC-G1**	Psych distress	T0	Prior mental ill-health	no prior MH	0.10	0.05	-0.01	0.20	1404
**ALSPAC-G1**	Psych distress	T0	Prior mental ill-health	prior MH	-0.09	0.12	-0.32	0.14	405
**ALSPAC-G1**	Psych distress	T1	Prior mental ill-health	no prior MH	0.16	0.08	0.00	0.33	1376
**ALSPAC-G1**	Psych distress	T1	Prior mental ill-health	prior MH	0.27	0.20	-0.13	0.67	398
**ALSPAC-G1**	Psych distress	T2	Prior mental ill-health	no prior MH	0.05	0.09	-0.14	0.24	1098
**ALSPAC-G1**	Psych distress	T2	Prior mental ill-health	prior MH	0.16	0.22	-0.27	0.58	315
**ALSPAC-G1**	Psych distress	T3	Prior mental ill-health	no prior MH	0.11	0.09	-0.06	0.28	1300
**ALSPAC-G1**	Psych distress	T3	Prior mental ill-health	prior MH	0.38	0.20	-0.02	0.78	375
**ALSPAC-G1**	Life satisfaction	T0	Sex	male	-0.33	0.17	-0.66	0.00	469
**ALSPAC-G1**	Life satisfaction	T0	Sex	female	-0.25	0.11	-0.47	-0.03	1251
**ALSPAC-G1**	Life satisfaction	T1	Sex	male	-0.38	0.16	-0.69	-0.08	587
**ALSPAC-G1**	Life satisfaction	T1	Sex	female	-0.33	0.10	-0.53	-0.13	1488
**ALSPAC-G1**	Life satisfaction	T2	Sex	male	-0.04	0.16	-0.36	0.28	456
**ALSPAC-G1**	Life satisfaction	T2	Sex	female	-0.23	0.12	-0.46	0.00	1160
**ALSPAC-G1**	Life satisfaction	T3	Sex	male	-0.23	0.16	-0.55	0.09	533
**ALSPAC-G1**	Life satisfaction	T3	Sex	female	-0.33	0.11	-0.53	-0.12	1387

**ALSPAC-G1**	Life satisfaction	T0	Prior mental ill-health	no prior MH	-0.20	0.08	-0.35	-0.06	1439
**ALSPAC-G1**	Life satisfaction	T0	Prior mental ill-health	prior MH	0.03	0.10	-0.17	0.24	281
**ALSPAC-G1**	Life satisfaction	T1	Prior mental ill-health	no prior MH	-0.38	0.10	-0.58	-0.19	1418
**ALSPAC-G1**	Life satisfaction	T1	Prior mental ill-health	prior MH	0.00	0.21	-0.41	0.40	278
**ALSPAC-G1**	Life satisfaction	T2	Prior mental ill-health	no prior MH	-0.14	0.11	-0.36	0.07	1123
**ALSPAC-G1**	Life satisfaction	T2	Prior mental ill-health	prior MH	0.23	0.22	-0.21	0.67	228
**ALSPAC-G1**	Life satisfaction	T3	Prior mental ill-health	no prior MH	-0.23	0.10	-0.42	-0.03	1336
**ALSPAC-G1**	Life satisfaction	T3	Prior mental ill-health	prior MH	-0.11	0.20	-0.51	0.29	264
**ALSPAC-G0**	Psych distress	T0	Sex	male	0.08	0.14	-0.19	0.35	755
**ALSPAC-G0**	Psych distress	T0	Sex	female	0.25	0.08	0.09	0.42	1947
**ALSPAC-G0**	Psych distress	T1	Sex	male	0.31	0.11	0.10	0.53	843
**ALSPAC-G0**	Psych distress	T1	Sex	female	0.54	0.08	0.38	0.70	2334
**ALSPAC-G0**	Psych distress	T2	Sex	male	0.38	0.11	0.16	0.61	743
**ALSPAC-G0**	Psych distress	T2	Sex	female	0.48	0.08	0.32	0.65	2003
**ALSPAC-G0**	Psych distress	T3	Sex	male	0.31	0.12	0.08	0.54	739
**ALSPAC-G0**	Psych distress	T3	Sex	female	0.37	0.08	0.20	0.53	2069
**ALSPAC-G0**	Psych distress	T0	Prior mental ill-health	no prior MH	0.02	0.05	-0.09	0.12	2223
**ALSPAC-G0**	Psych distress	T0	Prior mental ill-health	prior MH	0.33	0.09	0.15	0.50	479
**ALSPAC-G0**	Psych distress	T1	Prior mental ill-health	no prior MH	0.38	0.07	0.25	0.51	2121
**ALSPAC-G0**	Psych distress	T1	Prior mental ill-health	prior MH	0.61	0.21	0.19	1.03	458
**ALSPAC-G0**	Psych distress	T2	Prior mental ill-health	no prior MH	0.34	0.07	0.21	0.48	1889
**ALSPAC-G0**	Psych distress	T2	Prior mental ill-health	prior MH	0.78	0.22	0.34	1.22	390
**ALSPAC-G0**	Psych distress	T3	Prior mental ill-health	no prior MH	0.21	0.07	0.07	0.34	1951
**ALSPAC-G0**	Psych distress	T3	Prior mental ill-health	prior MH	0.55	0.21	0.13	0.97	398
**ALSPAC-G0**	Psych distress	T0	Age	45-54	0.32	0.26	-0.18	0.83	447
**ALSPAC-G0**	Psych distress	T0	Age	55-64	0.30	0.08	0.13	0.46	1954
**ALSPAC-G0**	Psych distress	T0	Age	65-74	-0.29	0.15	-0.59	0.01	292
**ALSPAC-G0**	Psych distress	T0	Age	75+	-0.91	1.92	-6.23	4.41	9
**ALSPAC-G0**	Psych distress	T1	Age	45-54	0.38	0.25	-0.11	0.88	576
**ALSPAC-G0**	Psych distress	T1	Age	55-64	0.51	0.07	0.36	0.66	2250
**ALSPAC-G0**	Psych distress	T1	Age	65-74	0.42	0.14	0.15	0.69	342
**ALSPAC-G0**	Psych distress	T1	Age	75+	-0.55	0.79	-2.73	1.64	9
**ALSPAC-G0**	Psych distress	T2	Age	45-54	0.52	0.26	0.01	1.03	442

**ALSPAC-G0**	Psych distress	T2	Age	55-64	0.47	0.08	0.31	0.63	1976
**ALSPAC-G0**	Psych distress	T2	Age	65-74	0.41	0.13	0.16	0.66	320
**ALSPAC-G0**	Psych distress	T2	Age	75+	-0.17	0.72	-2.45	2.11	8
**ALSPAC-G0**	Psych distress	T3	Age	45-54	0.48	0.31	-0.13	1.09	368
**ALSPAC-G0**	Psych distress	T3	Age	55-64	0.33	0.08	0.17	0.49	2054
**ALSPAC-G0**	Psych distress	T3	Age	65-74	0.37	0.14	0.10	0.64	376
**ALSPAC-G0**	Psych distress	T3	Age	75+	-0.19	1.25	-3.41	3.04	10

Note: TP = timepoint; coef = coefficient; se = standard error ; l_ci = lower 95% confidence interval ; u_ci = cupper 95% confidence interval. Models adjust for covariates.

McElroy E, *et al. BMJ Ment Health* 2023; 26:e300842. doi: 10.1136/bmjment-2023-300842

Table S7. Regression coefficients from sensitivity analysis (excluding those living in households of larger than ten)

cohort	outcome	exposure	coef	coef_se	lower_ci	upper_ci	n
**NCDS**	T1 Psych distress	T1 living alone alternative coding	0.07	0.05	-0.03	0.16	3566
**NCDS**	T2 Psych distress	T1 living alone alternative coding	0.04	0.04	-0.04	0.12	3691
**NCDS**	T3 Psych distress	T1 living alone alternative coding	0.01	0.04	-0.07	0.09	3723
**NCDS**	T2 Psych distress	T2 living alone	0.07	0.04	-0.01	0.15	3682
**NCDS**	T3 Psych distress	T3 living alone	0.07	0.04	-0.01	0.15	3719
**NCDS**	T2 Life satisfaction	T2 living alone	-0.30	0.04	-0.38	-0.21	3685
**NCDS**	T3 Life satisfaction	T3 living alone	-0.29	0.05	-0.38	-0.20	3727
**NCDS**	T2 Psych distress (binary)	T2 living alone	0.27	0.12	0.04	0.50	3681
**NCDS**	T3 Psych distress (binary)	T3 living alone	0.14	0.12	-0.10	0.39	3716
**BCS**	T1 Psych distress	T1 living alone alternative coding	-0.04	0.07	-0.18	0.09	2616
**BCS**	T2 Psych distress	T1 living alone alternative coding	0.04	0.06	-0.08	0.17	2673
**BCS**	T3 Psych distress	T1 living alone alternative coding	0.11	0.06	-0.01	0.24	2725
**BCS**	T2 Psych distress	T2 living alone	0.05	0.07	-0.08	0.17	2669
**BCS**	T3 Psych distress	T3 living alone	0.07	0.06	-0.05	0.19	2720
**BCS**	T2 Life satisfaction	T2 living alone	-0.35	0.06	-0.47	-0.24	2674
**BCS**	T3 Life satisfaction	T3 living alone	-0.19	0.06	-0.31	-0.07	2726
**BCS**	T2 Psych distress (binary)	T2 living alone	0.04	0.16	-0.27	0.35	2668
**BCS**	T3 Psych distress (binary)	T3 living alone	0.20	0.16	-0.10	0.51	2719
**NS**	T1 Psych distress	T1 living alone alternative coding	0.36	0.13	0.10	0.62	1131
**NS**	T2 Psych distress	T1 living alone alternative coding	0.16	0.09	-0.02	0.35	1178
**NS**	T3 Psych distress	T1 living alone alternative coding	0.08	0.09	-0.10	0.27	1244
**NS**	T2 Psych distress	T2 living alone	0.09	0.10	-0.10	0.28	1182
**NS**	T3 Psych distress	T3 living alone	-0.08	0.09	-0.24	0.09	1245
**NS**	T2 Life satisfaction	T2 living alone	-0.12	0.06	-0.25	0.00	1186
**NS**	T3 Life satisfaction	T3 living alone	-0.01	0.07	-0.15	0.13	1250
**NS**	T2 Psych distress (binary)	T2 living alone	0.03	0.21	-0.38	0.44	1182
**NS**	T3 Psych distress (binary)	T3 living alone	0.29	0.20	-0.09	0.68	1248

McElroy E, *et al. BMJ Ment Health* 2023; 26:e300842. doi: 10.1136/bmjment-2023-300842

**ELSA**	T1 Psych distress	T1 living alone alternative coding	0.00	0.05	-0.09	0.10	5470
**ELSA**	T3 Psych distress	T1 living alone alternative coding	-0.04	0.05	-0.13	0.06	5046
**ELSA**	T3 Psych distress	T3 living alone	0.21	0.04	0.12	0.29	5045
**ELSA**	T3 Life satisfaction	T3 living alone	-0.17	0.04	-0.26	-0.09	5037
**ELSA**	T3 Psych distress (binary)	T3 living alone	0.09	0.02	0.05	0.12	5045
**USOC**	T1 Psych distress	T1 living alone alternative coding	0.17	0.03	0.12	0.23	12,087
**USOC**	T2 Psych distress	T1 living alone alternative coding	0.15	0.03	0.09	0.21	9,904
**USOC**	T3 Psych distress	T1 living alone alternative coding	0.12	0.03	0.06	0.18	9,912
**USOC**	T1 Life satisfaction	T1 living alone alternative coding	-0.26	0.04	-0.35	-0.18	10,132
**USOC**	T2 Life satisfaction	T1 living alone alternative coding	-0.17	0.03	-0.22	-0.11	9,904
**USOC**	T3 life satisfaction (binary)	T1 living alone alternative coding	-0.20	0.03	-0.25	-0.14	9,912
**USOC**	T2 Psych distress	T2 living alone	0.12	0.03	0.07	0.17	9905
**USOC**	T3 Psych distress	T3 living alone	0.10	0.03	0.04	0.15	9913
**USOC**	T2 Life satisfaction	T2 living alone	-0.17	0.03	-0.23	-0.12	9905
**USOC**	T3 Life satisfaction	T3 living alone	-0.16	0.03	-0.21	-0.11	9913
**USOC**	T2 Psych distress (binary)	T2 living alone	0.30	0.05	0.19	0.40	9905
**USOC**	T3 Psych distress (binary)	T3 living alone	0.19	0.05	0.10	0.28	9913
**USOC**	T2 life satisfaction (binary)	T2 living alone	0.25	0.04	0.18	0.33	9905
**USOC**	T3 life satisfaction (binary)	T3 living alone	0.22	0.03	0.16	0.29	9913
**ALSPAC-G1**	T1 Psych distress	T1 living alone alternative coding	0.27	0.08	0.10	0.43	2062
**ALSPAC-G1**	T2 Psych distress	T1 living alone alternative coding	0.18	0.09	-0.01	0.36	1609
**ALSPAC-G1**	T3 Psych distress	T1 living alone alternative coding	0.26	0.09	0.09	0.44	1917
**ALSPAC-G1**	T2 Psych distress	T2 living alone	0.15	0.09	-0.02	0.33	1618
**ALSPAC-G1**	T3 Psych distress	T3 living alone	0.20	0.07	0.05	0.35	1964
**ALSPAC-G1**	T2 Life satisfaction	T2 living alone	-0.16	0.09	-0.33	0.02	1624
**ALSPAC-G1**	T3 Life satisfaction	T3 living alone	-0.24	0.08	-0.39	-0.10	1965

McElroy E, *et al. BMJ Ment Health* 2023; 26:e300842. doi: 10.1136/bmjment-2023-300842

**ALSPAC-G0**	T1 Psych distress	T1 living alone alternative coding	0.48	0.07	0.35	0.61	3175
**ALSPAC-G0**	T2 Psych distress	T1 living alone alternative coding	0.45	0.07	0.32	0.59	2744
**ALSPAC-G0**	T3 Psych distress	T1 living alone alternative coding	0.35	0.07	0.21	0.48	2806
**ALSPAC-G0**	T2 Psych distress	T2 living alone	0.46	0.07	0.33	0.59	2797
**ALSPAC-G0**	T3 Psych distress	T3 living alone	0.38	0.06	0.26	0.50	2873

Note: TP = timepoint; coef = coefficient; se = standard error ; l_ci = lower 95% confidence interval ; u_ci = cupper 95% confidence interval. Models adjust for covariates.

Table S8. Results from longitudinal multi-level models (time x lone household interactions)

cohort	outcome (cont outcomes only)	Model	exposure	TP	coef_interaction	se	l_ci	u_ci	main effect (time)	se	l_ci	u_ci	main effect (Lone HH)	se	L_ci	u_ci	n	
**NCDS**	Psych distress	Unadjusted	living alone	1	-0.04	0.05	-0.14	0.06	-0.03	0.02	-0.07	0.00	0.14	0.05	0.04	0.23	3928	
**NCDS**	Psych distress	Unadjusted	living alone	2	-0.07	0.05	-0.17	0.03	0.10	0.02	0.07	0.14	--	--	--	--	--	
**NCDS**	Psych distress	Unadjusted	living alone	3	-0.10	0.05	-0.21	0.00	0.07	0.02	0.04	0.11	--	--	--	--	--	
**NCDS**	Psych distress	Adjusted	living alone	1	-0.03	0.05	-0.13	0.07	-0.03	0.02	-0.06	0.01	0.09	0.05	-0.01	0.18	3928	
**NCDS**	Psych distress	Adjusted	living alone	2	-0.06	0.05	-0.16	0.04	0.11	0.02	0.07	0.14	--	--	--	--	--	
**NCDS**	Psych distress	Adjusted	living alone	3	-0.09	0.05	-0.20	0.01	0.07	0.02	0.04	0.11	--	--	--	--	--	
**BCS**	Psych distress	Unadjusted	living alone	1	-0.05	0.06	-0.16	0.06	0.02	0.02	-0.01	0.05	0.06	0.06	-0.05	0.18	3026	
**BCS**	Psych distress	Unadjusted	living alone	2	-0.01	0.06	-0.12	0.11	0.16	0.02	0.13	0.20	--	--	--	--	--	
**BCS**	Psych distress	Unadjusted	living alone	3	0.06	0.06	-0.06	0.18	0.09	0.02	0.05	0.12	--	--	--	--	--	
**BCS**	Psych distress	Adjusted	living alone	1	-0.06	0.06	-0.17	0.06	0.02	0.02	-0.01	0.05	0.03	0.06	-0.08	0.15	3026	
**BCS**	Psych distress	Adjusted	living alone	2	-0.02	0.06	-0.13	0.10	0.17	0.02	0.13	0.20	--	--	--	--	--	
**BCS**	Psych distress	Adjusted	living alone	3	0.05	0.06	-0.07	0.17	0.09	0.02	0.05	0.12	--	--	--	--	--	
**Next Steps**	Psych distress	Unadjusted	living alone	1	0.21	0.13	-0.04	0.47	0.32	0.04	0.24	0.40	0.01	0.09	-0.16	0.17	4555	
**Next Steps**	Psych distress	Unadjusted	living alone	2	0.14	0.12	-0.10	0.37	0.28	0.04	0.20	0.36	--	--	--	--	--	
**Next Steps**	Psych distress	Unadjusted	living alone	3	0.09	0.12	-0.14	0.32	0.36	0.04	0.29	0.44	--	--	--	--	--	
**Next Steps**	Psych distress	Adjusted	living alone	1	0.19	0.12	-0.05	0.43	0.34	0.04	0.27	0.42	0.06	0.09	-0.12	0.24	4555	
**Next Steps**	Psych distress	Adjusted	living alone	2	0.05	0.11	-0.16	0.26	0.27	0.04	0.19	0.34	--	--	--	--	--	
**Next Steps**	Psych distress	Adjusted	living alone	3	0.08	0.11	-0.14	0.29	0.33	0.04	0.26	0.40	--	--	--	--	--	
																	(obs)	*(groups)*
**ELSA**	Psych distress	Unadjusted	living alone	1	-0.03	0.06	-0.15	0.08	-0.01	0.02	-0.05	0.03	0.34	0.05	0.25	0.43	15139	5047
**ELSA**	Psych distress	Unadjusted	living alone	3	-0.10	0.05	-0.21	0.00	0.02	0.02	-0.02	0.07	--	--	--	--	--	
**ELSA**	Psych distress	Adjusted	living alone	1	-0.03	0.06	-0.14	0.08	0.00	0.02	-0.05	0.04	0.26	0.04	0.17	0.34	15139	5047
**ELSA**	Psych distress	Adjusted	living alone	3	-0.10	0.05	-0.20	0.00	0.03	0.02	-0.01	0.07	--	--	--	--	--	
**TwinsUK**	Psych distress	Unadjusted	Live Alone	1	-0.08	0.04	-0.16	0.00	0.24	0.02	0.21	0.28	0.08	0.05	-0.01	0.18	2327	
**TwinsUK**	Psych distress	Unadjusted	Live Alone	2	-0.10	0.04	-0.19	-0.02	0.49	0.02	0.45	0.52	--	--	--	--	--	

**TwinsUK**	Psych distress	Unadjusted	Live Alone	3	-0.11	0.04	-0.19	-0.03	0.54	0.02	0.50	0.57					2327
**TwinsUK**	Psych distress	Adjusted	Live Alone	1	-0.08	0.04	-0.16	0.00	0.24	0.02	0.21	0.28	0.10	0.05	0.01	0.20	2327
**TwinsUK**	Psych distress	Adjusted	Live Alone	2	-0.10	0.04	-0.19	-0.02	0.49	0.02	0.45	0.52	--	--	--	--	--
**TwinsUK**	Psych distress	Adjusted	Live Alone	3	-0.11	0.04	-0.19	-0.03	0.54	0.02	0.50	0.57					2327
**GS**	Psych distress	Unadjusted	Live Alone	1	0.02	0.06	-0.08	0.11	0.31	0.02	0.27	0.35	0.12	0.04	0.03	0.22	2882
**GS**	Psych distress	Unadjusted	Live Alone	2	0.11	0.06	0.00	0.21	0.22	0.02	0.18	0.26	--	--	--	--	--
**GS**	Psych distress	Unadjusted	Live Alone	3	0.05	0.07	-0.06	0.16	0.47	0.02	0.42	0.51	--	--	--	--	--
**GS**	Psych distress	Adjusted	Live Alone	1	0.02	0.06	-0.08	0.11	0.31	0.02	0.27	0.35	0.12	0.04	0.02	0.21	2882
**GS**	Psych distress	Adjusted	Live Alone	2	0.11	0.06	0.01	0.22	0.22	0.02	0.18	0.27	--	--	--	--	--
**GS**	Psych distress	Adjusted	Live Alone	3	0.05	0.07	-0.06	0.15	0.47	0.02	0.43	0.51	--	--	--	--	--
**USOC**	Psych distress	Unadjusted	living alone	1	0.05	0.04	-0.03	0.12	0.20	0.01	0.18	0.23	0.04	0.04	-0.03	0.11	12270
**USOC**	Psych distress	Unadjusted	living alone	2	0.05	0.04	-0.03	0.12	0.10	0.01	0.08	0.12	--	--	--	--	--
**USOC**	Psych distress	Unadjusted	living alone	3	0.00	0.04	-0.07	0.08	0.26	0.01	0.23	0.28	--	--	--	--	--
**USOC**	Psych distress	Adjusted	living alone	1	0.05	0.04	-0.02	0.12	0.20	0.01	0.18	0.23	0.04	0.04	-0.02	0.11	12270
**USOC**	Psych distress	Adjusted	living alone	2	0.05	0.04	-0.02	0.12	0.10	0.01	0.08	0.13	--	--	--	--	--
**USOC**	Psych distress	Adjusted	living alone	3	0.01	0.04	-0.07	0.08	0.26	0.01	0.24	0.29	--	--	--	--	--
**ALSPAC-G1**	Psych distress	Unadjusted	living alone	1	0.04	0.08	-0.12	0.19	-0.10	0.02	-0.14	-0.07	0.26	0.09	0.08	0.44	2852
**ALSPAC-G1**	Psych distress	Unadjusted	living alone	2	-0.04	0.07	-0.18	0.11	-0.06	0.02	-0.10	-0.02	--	--	--	--	--
**ALSPAC-G1**	Psych distress	Unadjusted	living alone	3	0.04	0.08	-0.12	0.20	-0.06	0.02	-0.10	-0.01	--	--	--	--	--
**ALSPAC-G1**	Psych distress	Adjusted	living alone	1	0.04	0.08	-0.12	0.21	-0.11	0.02	-0.15	-0.06	0.21	0.09	0.03	0.39	2102
**ALSPAC-G1**	Psych distress	Adjusted	living alone	2	-0.02	0.08	-0.18	0.14	-0.07	0.02	-0.12	-0.02	--	--	--	--	--
**ALSPAC-G1**	Psych distress	Adjusted	living alone	3	0.08	0.09	-0.10	0.25	-0.07	0.02	-0.11	-0.02	--	--	--	--	--
**ALSPAC-G0**	Psych distress	Unadjusted	living alone	1	0.15	0.09	-0.03	0.32	-0.73	0.02	-0.77	-0.69	0.27	0.09	0.09	0.45	3434
**ALSPAC-G0**	Psych distress	Unadjusted	living alone	2	0.17	0.09	-0.01	0.34	-0.68	0.02	-0.72	-0.64	--	--	--	--	--
**ALSPAC-G0**	Psych distress	Unadjusted	living alone	3	0.08	0.09	-0.08	0.25	-0.64	0.02	-0.68	-0.60	--	--	--	--	--
**ALSPAC-G0**	Psych distress	Adjusted	living alone	1	0.13	0.09	-0.05	0.31	-0.73	0.02	-0.77	-0.69	0.23	0.09	0.05	0.41	3326
**ALSPAC-G0**	Psych distress	Adjusted	living alone	2	0.16	0.09	-0.02	0.34	-0.69	0.02	-0.73	-0.65	--	--	--	--	--
**ALSPAC-G0**	Psych distress	Adjusted	living alone	3	0.09	0.09	-0.08	0.26	-0.65	0.02	-0.69	-0.61	--	--	--	--	--

Table S9. Results from the meta-analysis in LPS

	Time point	SMD	Lower CI	Upper CI	I^2
**Continuous Psychological Distress**	0	0.093	0.044	0.143	58.54
	1	0.191	0.092	0.289	87.21
	2	0.153	0.06	0.246	82.77
	3	0.146	0.074	0.218	76.04
**Binary Psychological Distress**	0	1.251	1.116	1.386	85.39
	1	1.312	1.03	1.593	96.75
	2	1.262	1.008	1.516	95.84
	3	1.307	1.148	1.466	91.89
**Continuous Life Satisfaction**	0	-0.251	-0.458	-0.044	97.85
	1	-0.223	-0.296	-0.15	66.26
	2	-0.222	-0.29	-0.155	66.61
	3	-0.191	-0.236	-0.146	35.38
**Binary Life Satisfaction**	0	1.056	0.779	1.333	97.76
	1	1.04	0.882	1.198	93.5
	2	1.05	0.806	1.293	97.48
	3	1.071	0.906	1.236	95.18

Table S10. Main effects from EHR analyses

	Anxiety				Depression				Eating disorder			
	Coefficient	SE	95% CI	P value	Coefficient	SE	95% CI	P value	Coefficient	SE	95% CI	P value
**Effect of not living alone pre-pandemic**	-13.63	3.67	(-20.93, -6.33)	<0.001	-25.56	3.89	(-33.30, -17.82)	<0.001	-2.88	0.19	(-3.27, -2.49)	<0.001
**Effect of the pandemic in those living alone**	-48.07	5.06	(-58.12,-38.02)	<0.001	-66.12	4.96	(-75.99,-56.25)	<0.001	0.1	0.4	(-0.70,0.91)	0.798
**Effect of the pandemic among those not living**	5.67	7.55	(-9.34, 20.68)	0.455	13.42	6.89	(-0.29, 27.12)	0.055	0.49	0.52	(-0.55, 1.52)	0.352
**Summer**	7.06	6.01	(-4.89, 19.00)	0.244	8.81	6.05	(-3.22, 20.83)	0.149	0.43	0.4	(-0.37, 1.22)	0.291
**Autumn**	22.19	6.11	(10.05, 34.33)	<0.001	24.78	6.38	(12.09, 37.47)	<0.001	0.25	0.38	(-0.51, 1.00)	0.521
**Winter**	1.97	6.83	(-11.60, 15.55)	0.773	8.36	6.5	(-4.55, 21.27)	0.201	0.05	0.4	(-0.75, 0.85)	0.906
**Constant**	244.48	5.08	(234.39, 254.57)	<0.001	212.59	5.53	(201.60, 223.58)	<0.001	9.49	0.29	(8.91, 10.06)	<0.001

	OCD				Self harm				Severe mental illness			
	Coefficient	SE	95% CI	P value	Coefficient	SE	95% CI	P value	Coefficient	SE	95% CI	P value
**Effect of not living alone pre-pandemic**	-0.31	0.11	(-0.53, -0.08)	0.009	-25.83	0.85	(-27.53, -24.14)	<0.001	-57.96	1.97	(-61.87, -54.06)	<0.001
**Effect of the pandemic in those living alone**	-0.61	0.14	(-0.89,-0.34)	<0.001	-6.99	1.44	(-9.85,-4.13)	<0.001	-7.48	2.79	(-13.03,-1.92)	0.009
**Effect of the pandemic among those not living**	0.05	0.17	(-0.30, 0.39)	0.795	7.95	1.68	(4.62, 11.29)	<0.001	4.40	3.00	(-1.57, 10.37)	0.147
**Summer**	-0.01	0.14	(-0.28, 0.27)	0.966	3.02	1.06	(0.93, 5.12)	0.005	0.33	1.97	(-3.57, 4.24)	0.866
**Autumn**	0.06	0.14	(-0.22, 0.34)	0.654	-0.36	0.95	(-2.24, 1.53)	0.708	2.49	1.92	(-1.33, 6.32)	0.199
**Winter**	0.00	0.14	(-0.27, 0.28)	0.989	-2.08	1.14	(-4.34, 0.18)	0.071	3.74	2.54	(-1.31, 8.80)	0.144
**Constant**	3.25	0.12	(3.01, 3.49)	<0.001	52.32	0.94	(50.46, 54.18)	<0.001	88.36	2.1	(84.19, 92.53)	<0.001

Mental health outcomes as defined in Table S1.

Table S11. Sex-stratified estimates from EHR analyses

	Anxiety				Depression				Eating disorder			
	Coefficient	SE	95% CI	P value	Coefficient	SE	95% CI	P value	Coefficient	SE	95% CI	P value
**Pre-pandemic effects**												
Females not living alone	16.4	4.48	(7.56, 25.24)	<0.001	2.72	4.75	(-6.67, 12.10)	0.568	-4.24	0.36	(-4.94, -3.53)	<0.001
Males living alone	-88.63	3.8	(-96.12, -81.14)	<0.001	-34.16	4.51	(-43.05, -25.26)	<0.001	-12.88	0.34	(-13.54, -12.21)	<0.001
Males not living alone	-135.15	3.47	(-142.01, -128.29	<0.001	-88.65	3.95	(-96.45, -80.85)	<0.001	-14.26	0.33	(-14.92, -13.61)	<0.001
**Effect of the pandemic in those living a**	-37.89	6.08	(-49.88,-25.89)	<0.001	-65.09	5.45	(-75.86,-54.33)	<0.001	0.6	0.7	(-0.79,1.99)	0.395
**Interaction effects during pandemic**												
Females not living alone	-3.09	9.82	(-22.48, 16.29)	0.753	3.09	8.18	(-13.05, 19.23)	0.706	0.62	0.91	(-1.19, 2.42)	0.5
Males living alone	-8.43	7.52	(-23.27, 6.41)	0.264	-2.23	6.98	(-16.00, 11.54)	0.75	-0.97	0.71	(-2.38, 0.43)	0.173
Males not living alone	7.11	7.61	(-7.91, 22.14)	0.352	22.02	6.67	(8.86, 35.18)	0.001	-0.62	0.7	(-2.01, 0.77)	0.379
**Summer**	6.99	4.49	(-1.87, 15.85)	0.121	8.81	4.37	(0.18, 17.44)	0.045	0.42	0.36	(-0.30, 1.14)	0.249
**Autumn**	23.85	4.59	(14.79, 32.91)	<0.001	24.85	4.66	(15.66, 34.04)	<0.001	0.24	0.35	(-0.45, 0.93)	0.489
**Winter**	3.44	5.08	(-6.58, 13.46)	0.499	8.45	4.68	(-0.80, 17.69)	0.073	0.06	0.37	(-0.67, 0.79)	0.878
**Constant**	289.5	4.07	(281.46, 297.54)	<0.001	229.65	4.77	(220.24, 239.07)	<0.001	15.8	0.37	(15.08, 16.53)	<0.001

	OCD				Self harm				Severe mental illness			
	Coefficient	SE	95% CI	P value	Coefficient	SE	95% CI	P value	Coefficient	SE	95% CI	P value
**Pre-pandemic effects**												
Females not living alone	0.24	0.14	(-0.02, 0.51)	0.074	-26.29	1.02	(-28.31, -24.27)	<0.001	-49.45	1.98	(-53.35, -45.56)	<0.001
Males living alone	-0.18	0.16	(-0.50, 0.14)	0.263	-11.17	0.99	(-13.12, -9.22)	<0.001	8.64	2.61	(3.49, 13.80)	0.001
Males not living alone	-1.06	0.12	(-1.31, -0.82)	<0.001	-36.64	0.98	(-38.57, -34.71)	<0.001	-58.29	2	(-62.23, -54.34)	<0.001
**Effect of the pandemic in those living a**	-0.48	0.16	(-0.79,-0.17)	0.002	-8.2	1.58	(-11.33,-5.08)	<0.001	-4.4	2.93	(-10.18,1.38)	0.134
**Interaction effects during pandemic**												
Females not living alone	-0.1	0.21	(-0.52, 0.32)	0.641	9.4	1.88	(5.69, 13.11)	<0.001	0.79	3.17	(-5.48, 7.06)	0.804
Males living alone	-0.27	0.22	(-0.70, 0.15)	0.209	2.47	2.11	(-1.69, 6.63)	0.243	-6.38	3.94	(-14.16, 1.40)	0.107
Males not living alone	-0.06	0.17	(-0.41, 0.28)	0.717	8.91	1.75	(5.46, 12.35)	<0.001	1.86	3.11	(-4.28, 7.99)	0.551
**Summer**	0	0.11	(-0.21, 0.20)	0.963	3.04	0.77	(1.52, 4.57)	<0.001	0.33	1.41	(-2.44, 3.11)	0.813
**Autumn**	0.07	0.11	(-0.15, 0.29)	0.536	-0.34	0.71	(-1.74, 1.07)	0.638	2.5	1.37	(-0.22, 5.21)	0.071
**Winter**	0	0.11	(-0.21, 0.21)	0.987	-2.05	0.84	(-3.71, -0.40)	0.015	3.76	1.8	(0.21, 7.32)	0.038
**Constant**	3.34	0.12	(3.10, 3.59)	<0.001	57.94	0.99	(55.99, 59.89)	<0.001	84.27	1.98	(80.36, 88.17)	<0.001

NB: Except where specified, coefficients relate to effects in the baseline categories of all variables (pre-pandemic, spring, living alone, females)

Table S12. Age band-stratified estimates from EHR analyses

	Anxiety				Depression				Eating disorder			
	Coefficient	SE	95% CI	P value	Coefficient	SE	95% CI	P value	Coefficient	SE	95% CI	P value
**Pre-pandemic effects**												
Age 18-39 not living alone	-266.13	5.29	(-276.55, -255.72	<0.001	-215.01	5.25	(-225.34, -204.6	<0.001	-18.37	0.54	(-19.42, -17.32)	<0.001
Age 40-59 living alone	-308.98	5.27	(-319.34, -298.62	<0.001	-262.97	4.85	(-272.51, -253.4	<0.001	-19.59	0.52	(-20.61, -18.56)	<0.001
Age 40-59 not living alone	-298.61	5.87	(-310.14, -287.07	<0.001	-269.19	4.75	(-278.54, -259.8	<0.001	-18.45	0.54	(-19.50, -17.39)	<0.001
Age 60-79 living alone	-318.99	5.92	(-330.63, -307.36	<0.001	-282.19	4.94	(-291.92, -272.4	<0.001	-18.11	0.54	(-19.18, -17.05)	<0.001
Age 60-79 not living alone	-58.01	6.8	(-71.40, -44.63)	<0.001	-59.24	5.69	(-70.43, -48.06)	<0.001	-9.22	0.55	(-10.30, -8.14)	<0.001
Age 80+ living alone	-136.01	5.86	(-147.54, -124.47	<0.001	-69.54	7.08	(-83.46, -55.62)	<0.001	-12.9	0.55	(-13.97, -11.82)	<0.001
Age 80+ not living alone	-209.38	5.33	(-219.87, -198.89	<0.001	-160.58	5.26	(-170.93, -150.2	<0.001	-17.62	0.52	(-18.65, -16.60)	<0.001
**Effect of the pandemic in those living al**	-66.76	10.6	(-87.61,-45.90)	<0.001	-79.77	9.25	(-97.96,-61.58)	<0.001	0.87	1.22	(-1.53,3.27)	0.477
**Interaction effects during pandemic**												
Age 18-39 not living alone	10.27	14.6	(-18.44, 38.98)	0.482	10.88	12.19	(-13.10, 34.86)	0.373	1.27	1.42	(-1.51, 4.05)	0.37
Age 40-59 living alone	5.44	11.82	(-17.80, 28.67)	0.646	-23.08	11.41	(-45.52, -0.63)	0.044	-1.47	1.25	(-3.93, 0.99)	0.242
Age 40-59 not living alone	37.8	11.65	(14.90, 60.70)	0.001	24.54	10.38	(4.12, 44.96)	0.019	-1.04	1.23	(-3.46, 1.38)	0.399
Age 60-79 living alone	48.69	11.06	(26.93, 70.45)	<0.001	37.36	9.7	(18.28, 56.44)	<0.001	-0.8	1.23	(-3.22, 1.62)	0.516
Age 60-79 not living alone	59.89	11.2	(37.87, 81.91)	<0.001	59.17	9.62	(40.25, 78.08)	<0.001	-0.96	1.22	(-3.35, 1.43)	0.43
Age 80+ living alone	58.36	11.22	(36.28, 80.43)	<0.001	64.72	9.51	(46.01, 83.42)	<0.001	-0.85	1.23	(-3.27, 1.56)	0.488
Age 80+ not living alone	59.53	11.51	(36.88, 82.17)	<0.001	70.83	9.63	(51.89, 89.77)	<0.001	-0.99	1.24	(-3.42, 1.45)	0.426
**Summer**	7.06	3.43	(0.32, 13.81)	0.04	8.47	3.24	(2.09, 14.85)	0.009	0.32	0.3	(-0.26, 0.91)	0.279
**Autumn**	21.36	3.58	(14.32, 28.40)	<0.001	21.67	3.59	(14.60, 28.73)	<0.001	0.17	0.27	(-0.37, 0.70)	0.54
**Winter**	3.57	3.88	(-4.06, 11.20)	0.359	7.49	3.53	(0.55, 14.43)	0.035	0.08	0.29	(-0.48, 0.65)	0.77
**Constant**	409.92	5.24	(399.61, 420.23)	<0.001	338.14	5.06	(328.19, 348.08	<0.001	21.52	0.51	(20.51, 22.53)	<0.001

	OCD				Self harm				Severe mental illness			
	Coefficient	SE	95% CI	P value	Coefficient	SE	95% CI	P value	Coefficient	SE	95% CI	P value
**Pre-pandemic effects**												
Age 18-39 not living alone	-5.49	0.32	(-6.12, -4.87)	<0.001	-90.82	1.54	(-93.85, -87.80)	<0.001	6.97	3.34	(0.40, 13.53)	0.038
Age 40-59 living alone	-5.93	0.31	(-6.54, -5.32)	<0.001	-100.56	1.51	(-103.52, -97.5	<0.001	-46.86	1.49	(-49.80, -43.93)	<0.001
Age 40-59 not living alone	-6.43	0.31	(-7.05, -5.82)	<0.001	-98.63	1.56	(-101.70, -95.5	<0.001	-35.79	2.82	(-41.35, -30.24)	<0.001
Age 60-79 living alone	-6.4	0.31	(-7.01, -5.78)	<0.001	-101.43	1.5	(-104.37, -98.4	<0.001	-49.06	1.67	(-52.34, -45.77)	<0.001
Age 60-79 not living alone	-1.22	0.32	(-1.85, -0.58)	<0.001	-58.58	1.65	(-61.82, -55.34)	<0.001	-45.75	1.33	(-48.37, -43.12)	<0.001
Age 80+ living alone	-2.85	0.33	(-3.51, -2.20)	<0.001	-40.07	1.74	(-43.49, -36.65)	<0.001	55.06	2.72	(49.70, 60.42)	<0.001
Age 80+ not living alone	-4.77	0.31	(-5.39, -4.16)	<0.001	-86.08	1.53	(-89.09, -83.07)	<0.001	-42.59	1.45	(-45.44, -39.74)	<0.001
**Effect of the pandemic in those living al**	-0.65	0.44	(-1.51,0.21)	0.138	-20.46	3.02	(-26.40,-14.52)	<0.001	-16.36	1.9	(-20.10,-12.61)	<0.001
**Interaction effects during pandemic**												
Age 18-39 not living alone	-0.05	0.5	(-1.03, 0.92)	0.913	22.24	3.32	(15.71, 28.77)	<0.001	13.12	2.2	(8.81, 17.44)	<0.001
Age 40-59 living alone	-0.69	0.46	(-1.59, 0.21)	0.133	12.26	3.66	(5.06, 19.46)	0.001	-1.06	4.16	(-9.25, 7.13)	0.8
Age 40-59 not living alone	0.1	0.44	(-0.77, 0.96)	0.824	21.46	3.1	(15.36, 27.55)	<0.001	11.83	2.3	(7.31, 16.35)	<0.001
Age 60-79 living alone	0.45	0.44	(-0.42, 1.33)	0.308	21.47	3.09	(15.39, 27.54)	<0.001	18.82	4.5	(9.98, 27.66)	<0.001
Age 60-79 not living alone	0.44	0.44	(-0.42, 1.30)	0.315	21.81	3.08	(15.76, 27.86)	<0.001	15.04	2.31	(10.50, 19.58)	<0.001
Age 80+ living alone	0.64	0.44	(-0.23, 1.50)	0.148	23.92	3.13	(17.76, 30.07)	<0.001	26.57	3.5	(19.69, 33.45)	<0.001
Age 80+ not living alone	0.54	0.44	(-0.32, 1.40)	0.219	22.2	3.07	(16.16, 28.24)	<0.001	17.56	2.38	(12.87, 22.25)	<0.001
**Summer**	0.02	0.1	(-0.19, 0.22)	0.87	2.81	0.71	(1.43, 4.20)	<0.001	0	1.12	(-2.19, 2.20)	0.999
**Autumn**	0.08	0.11	(-0.13, 0.29)	0.46	-0.11	0.59	(-1.27, 1.05)	0.851	2.28	1.09	(0.13, 4.43)	0.038
**Winter**	0.03	0.1	(-0.16, 0.22)	0.738	-1.69	0.72	(-3.11, -0.26)	0.02	4	1.43	(1.19, 6.81)	0.005
**Constant**	6.66	0.3	(6.08, 7.24)	<0.001	105.9	1.48	(103.00, 108.81	<0.001	75.54	1.42	(72.75, 78.34)	<0.001

NB: Except where specified, coefficients relate to effects in the baseline categories of all variables (pre-pandemic, spring, living alone, age 18-40)

Table S13. Ethnicity-stratified estimates from EHR analyses

	Anxiety				Depression				Eating disorder				OCD				Self harm				Severe mental illness			
	Coefficien	SE	95% CI	P value	Coefficien	SE	95% CI	P value	Coefficien	SE	95% CI	P value	Coefficien	SE	95% CI	P value	Coefficien	SE	95% CI	P value	Coefficien	SE	95% CI	P value
**Pre-pandemic effects**																								
White not living alone	85.28	9.86	(65.91, 104.65)	<0.001	92.1	9.29	(73.85, 110.34)	<0.001	7.25	1.36	(4.59, 9.92)	<0.001	1.7	0.76	(0.21, 3.19)	0.026	27.63	2.84	(22.05, 33.21)	<0.001	96.47	4.49	(87.66, 105.29)	<0.001
Mixed living alone	46.66	4.4	(38.02, 55.31)	<0.001	37.2	4.37	(28.61, 45.79)	<0.001	2.42	0.76	(0.93, 3.91)	0.002	0.46	0.4	(-0.34, 1.25)	0.259	0.76	1.66	(-2.50, 4.03)	0.646	-11.69	2.51	(-16.62, -6.75)	<0.001
Mixed not living alone	-15.34	3.99	(-23.19, -7.50)	<0.001	-5.22	4.11	(-13.29, 2.86)	0.205	-3.29	0.45	(-4.18, -2.40)	<0.001	-0.08	0.3	(-0.68, 0.52)	0.798	-12.81	1.15	(-15.07, -10.56)	<0.001	36.7	2.88	(31.05, 42.36)	<0.001
Asian living alone	-61.52	3.09	(-67.58, -55.46)	<0.001	-63.63	2.94	(-69.40, -57.85)	<0.001	-4.31	0.32	(-4.94, -3.68)	<0.001	-0.33	0.24	(-0.79, 0.14)	0.165	-25.01	0.74	(-26.46, -23.55)	<0.001	-22.64	2.15	(-26.86, -18.42)	<0.001
Asian not living alone	-19.49	3.84	(-27.04, -11.94)	<0.001	0.64	6.44	(-12.01, 13.30)	0.921	-1.65	0.59	(-2.81, -0.49)	0.005	-1.01	0.38	(-1.75, -0.26)	0.008	-7.61	1.43	(-10.42, -4.80)	<0.001	116.59	3.83	(109.06, 124.12	<0.001
Black living alone	-75.11	3.78	(-82.54, -67.69)	<0.001	-62.95	3.1	(-69.04, -56.86)	<0.001	-3.45	0.38	(-4.20, -2.70)	<0.001	-1.72	0.25	(-2.22, -1.22)	<0.001	-23.51	0.89	(-25.25, -21.76)	<0.001	-12.7	2.42	(-17.46, -7.95)	<0.001
Black not living alone	-50.57	4.29	(-59.01, -42.14)	<0.001	-36.62	3.89	(-44.26, -28.98)	<0.001	-3.14	0.59	(-4.29, -1.98)	<0.001	-0.62	0.33	(-1.26, 0.03)	0.063	-15.21	1.13	(-17.43, -12.98)	<0.001	-3.84	3.46	(-10.64, 2.96)	0.268
Other living alone	-73.71	3.49	(-80.57, -66.85)	<0.001	-71.15	2.92	(-76.88, -65.41)	<0.001	-4.58	0.43	(-5.43, -3.73)	<0.001	-1.31	0.27	(-1.84, -0.79)	<0.001	-24.23	0.85	(-25.89, -22.56)	<0.001	-36.5	2.14	(-40.71, -32.30)	<0.001
Other not living alone	18.28	4.18	(10.06, 26.50)	<0.001	5.06	3.04	(-0.91, 11.02)	0.096	-1.09	0.31	(-1.69, -0.49)	<0.001	0.48	0.22	(0.05, 0.91)	0.027	-9.22	0.89	(-10.98, -7.47)	<0.001	-37.9	2.14	(-42.11, -33.69)	<0.001
Unknown living alone	71.73	3.89	(64.10, 79.36)	<0.001	63.81	4.25	(55.46, 72.16)	<0.001	3.08	0.34	(2.41, 3.75)	<0.001	0.76	0.24	(0.30, 1.23)	0.001	25	0.97	(23.10, 26.91)	<0.001	34.07	2.83	(28.51, 39.64)	<0.001
Unknown not living alone	57.48	3.92	(49.78, 65.19)	<0.001	38.83	3.48	(31.98, 45.67)	<0.001	-0.17	0.3	(-0.77, 0.42)	0.571	0.31	0.22	(-0.13, 0.75)	0.163	-5.5	0.82	(-7.12, -3.88)	<0.001	-27.15	2.18	(-31.44, -22.85)	<0.001
**Effect of the pandemic in those living alone and w**	-30.26	4.82	(-39.73,-20.80)	<0.001	-45.63	3.93	(-53.35,-37.90)	<0.001	0.96	0.4	(0.18,1.74)	0.015	-0.68	0.26	(-1.19,-0.16)	0.01	-2.11	1.03	(-4.14,-0.08)	0.042	-3.76	2.45	(-8.56,1.04)	0.125
**Interaction effects during pandemic**																								
White not living alone	-0.56	8.31	(-16.89, 15.78)	0.947	1.3	6.43	(-11.33, 13.93)	0.84	0.6	0.63	(-0.64, 1.84)	0.34	0.12	0.3	(-0.47, 0.70)	0.692	4.24	1.37	(1.56, 6.93)	0.002	2.28	2.62	(-2.88, 7.44)	0.386
Mixed living alone	-17.47	7.17	(-31.57, -3.38)	0.015	-29.04	6.42	(-41.64, -16.44)	<0.001	-1.17	0.6	(-2.36, 0.01)	0.053	0.01	0.3	(-0.58, 0.59)	0.986	-6.43	2.07	(-10.50, -2.37)	0.002	-3.55	3.84	(-11.11, 4.00)	0.356
Mixed not living alone	-9.99	8.06	(-25.81, 5.84)	0.216	-13.29	6.47	(-26.01, -0.57)	0.041	-0.54	0.51	(-1.54, 0.46)	0.289	0.06	0.29	(-0.50, 0.62)	0.83	2.81	1.32	(0.21, 5.41)	0.034	0.28	2.74	(-5.10, 5.66)	0.919
Asian living alone	21.59	13.06	(-4.06, 47.25)	0.099	10.57	12.9	(-14.77, 35.91)	0.413	2.59	2.11	(-1.56, 6.74)	0.221	-0.46	0.95	(-2.32, 1.40)	0.627	-8.46	3.54	(-15.41, -1.51)	0.017	-23.3	7.59	(-38.20, -8.39)	0.002
Asian not living alone	1.49	9.48	(-17.13, 20.11)	0.875	-1.01	8.65	(-18.00, 15.98)	0.907	-0.02	1.11	(-2.20, 2.16)	0.986	0.7	0.57	(-0.43, 1.82)	0.225	4.46	2.55	(-0.55, 9.47)	0.081	0.56	3.4	(-6.11, 7.23)	0.868
Black living alone	13.67	7.7	(-1.45, 28.80)	0.076	6.49	6.61	(-6.49, 19.46)	0.326	0.23	0.67	(-1.08, 1.54)	0.729	1.29	0.46	(0.39, 2.18)	0.005	3	1.76	(-0.45, 6.46)	0.088	-12.35	3.76	(-19.73, -4.96)	0.001
Black not living alone	18.97	5.97	(7.25, 30.70)	0.002	20.53	5.28	(10.17, 30.90)	<0.001	-0.95	0.47	(-1.87, -0.02)	0.044	0.28	0.3	(-0.31, 0.88)	0.351	3.33	1.21	(0.96, 5.70)	0.006	-0.02	2.74	(-5.41, 5.36)	0.993
Other living alone	15.22	8.33	(-1.14, 31.59)	0.068	6.1	9.76	(-13.07, 25.27)	0.532	-0.37	1.12	(-2.57, 1.82)	0.74	0.43	0.51	(-0.58, 1.44)	0.404	-1.65	2.28	(-6.13, 2.84)	0.472	-27.43	6.26	(-39.74, -15.12)	<0.001
Other not living alone	24.12	6.28	(11.78, 36.47)	<0.001	22.56	5.6	(11.55, 33.56)	<0.001	-1.16	0.63	(-2.40, 0.09)	0.068	0.4	0.31	(-0.21, 1.01)	0.201	2.58	1.39	(-0.15, 5.30)	0.064	0.15	3	(-5.75, 6.05)	0.96
Unknown living alone	22.9	7.58	(8.00, 37.80)	0.003	20.97	6.96	(7.30, 34.64)	0.003	-0.66	0.81	(-2.25, 0.93)	0.417	0.38	0.46	(-0.52, 1.29)	0.405	-1.53	1.73	(-4.92, 1.86)	0.375	-4.34	4.21	(-12.61, 3.92)	0.303
Unknown not living alone	15.62	6.2	(3.44, 27.80)	0.012	25.13	5.03	(15.25, 35.02)	<0.001	-0.08	0.58	(-1.22, 1.07)	0.894	0.59	0.34	(-0.07, 1.25)	0.079	2.75	1.29	(0.20, 5.29)	0.034	1.56	2.69	(-3.72, 6.84)	0.561
**Summer**	3.07	2.78	(-2.39, 8.53)	0.27	9.11	2.95	(3.31, 14.90)	0.002	0.6	0.29	(0.03, 1.18)	0.04	0.01	0.15	(-0.29, 0.31)	0.956	3.14	0.64	(1.88, 4.40)	<0.001	0.53	1.45	(-2.31, 3.37)	0.714
**Autumn**	13.92	2.75	(8.51, 19.33)	<0.001	19.9	2.95	(14.11, 25.69)	<0.001	0.13	0.3	(-0.47, 0.73)	0.676	0.22	0.16	(-0.10, 0.54)	0.17	0.05	0.62	(-1.17, 1.27)	0.937	2.28	1.41	(-0.48, 5.05)	0.105
**Winter**	1.86	3.03	(-4.11, 7.82)	0.541	11.49	3.23	(5.15, 17.83)	<0.001	0.33	0.37	(-0.40, 1.05)	0.38	0.11	0.16	(-0.20, 0.42)	0.48	-1.01	0.7	(-2.38, 0.37)	0.151	3.87	1.49	(0.95, 6.79)	0.009
**Constant**	198.33	3.16	(192.12, 204.53)	<0.001	166.56	2.96	(160.74, 172.38	<0.001	7.29	0.34	(6.62, 7.95)	<0.001	2.65	0.23	(2.20, 3.10)	<0.001	34.4	0.73	(32.96, 35.85)	<0.001	59.04	2.19	(54.75, 63.33)	<0.001

NB: Except where specified, coefficients relate to effects in the baseline categories of all variables (pre-pandemic, spring, living alone, White ethnicity)

Table S14. IMD-stratified estimates from EHR analyses

	Anxiety				Depression				Eating disorder				OCD				Self harm				Severe mental illness			
	Coefficient	SE	95% CI	P value	Coefficient	SE	95% CI	P value	Coefficient	SE	95% CI	P value	Coefficient	SE	95% CI	P value	Coefficient	SE	95% CI	P value	Coefficient	SE	95% CI	P value
**Pre-pandemic effects, by quintile of deprivation**																								
IMD 1 (least deprived), not living alone	-86.84	5.23	(-97.11, -76.57)	<0.001	-101.47	5.98	(-113.23, -89.70	<0.001	-1.18	0.37	(-1.90, -0.46)	0.001	-0.45	0.26	(-0.97, 0.07)	0.09	-54.51	1.5	(-57.46, -51.56)	<0.001	-73.14	2.86	(-78.76, -67.51)	<0.001
IMD 2, living alone	-95.64	5.4	(-106.25, -85.03)	<0.001	-117.42	5.91	(-129.03, -105.8	<0.001	-3.76	0.27	(-4.30, -3.23)	<0.001	-0.74	0.22	(-1.17, -0.31)	0.001	-73.71	1.49	(-76.65, -70.78)	<0.001	-120.29	2.29	(-124.80, -115.	<0.001
IMD 2, not living alone	-105.07	5.19	(-115.28, -94.87)	<0.001	-120.73	6.03	(-132.58, -108.8	<0.001	-2.27	0.35	(-2.95, -1.58)	<0.001	-0.75	0.26	(-1.26, -0.24)	0.004	-63.79	1.5	(-66.75, -60.84)	<0.001	-88.57	2.84	(-94.14, -82.99)	<0.001
IMD 3, living alone	-110.56	5.25	(-120.88, -100.24	<0.001	-135.75	5.94	(-147.42, -124.0	<0.001	-4.33	0.27	(-4.87, -3.79)	<0.001	-0.9	0.21	(-1.31, -0.49)	<0.001	-78.23	1.47	(-81.11, -75.35)	<0.001	-125.56	2.26	(-130.01, -121.	<0.001
IMD 3, not living alone	-116.84	5.04	(-126.75, -106.93	<0.001	-137.25	6.31	(-149.64, -124.8	<0.001	-2.63	0.36	(-3.34, -1.91)	<0.001	-0.61	0.25	(-1.10, -0.12)	0.016	-72.47	1.49	(-75.39, -69.55)	<0.001	-101.03	2.67	(-106.29, -95.7	<0.001
IMD 4, living alone	-125.41	5.16	(-135.55, -115.27	<0.001	-153.96	5.82	(-165.40, -142.5	<0.001	-4.35	0.28	(-4.90, -3.80)	<0.001	-0.82	0.21	(-1.24, -0.41)	<0.001	-81.84	1.5	(-84.79, -78.89)	<0.001	-129.33	2.26	(-133.77, -124.	<0.001
IMD 4, not living alone	-22.04	5.97	(-33.76, -10.31)	<0.001	-39.18	6.51	(-51.97, -26.39)	<0.001	-3.23	0.3	(-3.83, -2.64)	<0.001	-0.37	0.22	(-0.81, 0.06)	0.093	-52.07	1.64	(-55.30, -48.84)	<0.001	-100.69	2.31	(-105.23, -96.1	<0.001
IMD 5 (most deprived), living alone	-54.79	5.59	(-65.77, -43.80)	<0.001	-62.49	6.28	(-74.84, -50.15)	<0.001	0.65	0.46	(-0.24, 1.55)	0.152	-0.15	0.23	(-0.61, 0.31)	0.514	-38.45	1.62	(-41.64, -35.27)	<0.001	-45.22	2.88	(-50.89, -39.56)	<0.001
IMD 5 (most deprived), not living alone	-73.54	5.25	(-83.86, -63.21)	<0.001	-90.4	5.94	(-102.07, -78.73	<0.001	-3.71	0.29	(-4.28, -3.13)	<0.001	-0.62	0.21	(-1.04, -0.20)	0.004	-66.33	1.44	(-69.17, -63.49)	<0.001	-112.49	2.3	(-117.00, -107.	<0.001
**Effect of the pandemic in those living alo**	-69.05	6.69	(-82.20,-55.90)	<0.001	-103.46	6.86	(-116.93,-89.98)	<0.001	0.43	0.37	(-0.31,1.16)	0.252	-0.84	0.24	(-1.30,-0.37)	<0.001	-14.9	3.23	(-21.25,-8.56)	<0.001	-11.96	4.22	(-20.26,-3.67)	0.005
**Interaction effects during pandemic**																								
IMD 1 (least deprived), not living alone	25.82	10.32	(5.54, 46.10)	0.013	34.88	10.11	(15.01, 54.75)	0.001	0.38	0.55	(-0.70, 1.46)	0.494	0.18	0.28	(-0.37, 0.74)	0.522	15.64	3.51	(8.74, 22.55)	<0.001	6.91	4.47	(-1.88, 15.69)	0.123
IMD 2, living alone	34.9	8.94	(17.32, 52.47)	<0.001	38.98	8.68	(21.92, 56.05)	<0.001	-0.23	0.79	(-1.78, 1.32)	0.771	0.35	0.31	(-0.26, 0.95)	0.261	8.76	3.67	(1.54, 15.98)	0.018	4.21	5.14	(-5.89, 14.30)	0.413
IMD 2, not living alone	32.18	9.1	(14.30, 50.06)	<0.001	46.97	8.5	(30.26, 63.68)	<0.001	0.47	0.53	(-0.56, 1.51)	0.369	0.19	0.27	(-0.34, 0.72)	0.488	16.31	3.36	(9.72, 22.91)	<0.001	8.56	4.4	(-0.09, 17.21)	0.052
IMD 3, living alone	32.12	8.27	(15.86, 48.39)	<0.001	47.38	8.34	(30.98, 63.78)	<0.001	-0.05	0.69	(-1.41, 1.31)	0.939	0.34	0.32	(-0.30, 0.98)	0.293	9.08	3.46	(2.28, 15.89)	0.009	4.29	4.97	(-5.48, 14.05)	0.389
IMD 3, not living alone	34.1	8.71	(16.98, 51.22)	<0.001	53.26	8.12	(37.31, 69.21)	<0.001	-0.02	0.52	(-1.04, 0.99)	0.965	0.15	0.28	(-0.40, 0.70)	0.589	15.99	3.34	(9.43, 22.54)	<0.001	9.03	4.38	(0.42, 17.63)	0.04
IMD 4, living alone	34.7	8.31	(18.37, 51.03)	<0.001	48.59	8.1	(32.67, 64.51)	<0.001	-0.36	0.54	(-1.42, 0.69)	0.499	0.39	0.33	(-0.27, 1.04)	0.246	10.92	3.41	(4.22, 17.62)	0.001	7.25	4.84	(-2.26, 16.76)	0.135
IMD 4, not living alone	36.2	8.75	(19.00, 53.40)	<0.001	56.93	8.08	(41.05, 72.81)	<0.001	0.16	0.52	(-0.85, 1.18)	0.752	0.47	0.28	(-0.08, 1.01)	0.093	15.57	3.33	(9.03, 22.11)	<0.001	9.34	4.32	(0.84, 17.83)	0.031
IMD 5 (most deprived), living alone	33.47	8.11	(17.54, 49.41)	<0.001	54.35	7.98	(38.67, 70.03)	<0.001	-1.28	0.64	(-2.55, -0.02)	0.047	-0.05	0.31	(-0.67, 0.56)	0.867	11.5	3.34	(4.94, 18.06)	0.001	7.11	4.72	(-2.17, 16.39)	0.133
IMD 5 (most deprived), not living alone	36.13	8.61	(19.21, 53.05)	<0.001	61.04	7.96	(45.39, 76.69)	<0.001	-0.16	0.51	(-1.15, 0.84)	0.76	0.37	0.27	(-0.16, 0.91)	0.171	15.58	3.34	(9.02, 22.14)	<0.001	10.4	4.33	(1.89, 18.90)	0.017
**Summer**	6.99	2.82	(1.46, 12.52)	0.013	8.7	2.81	(3.19, 14.22)	0.002	0.42	0.21	(0.00, 0.84)	0.049	0	0.09	(-0.18, 0.18)	0.982	3	0.59	(1.84, 4.16)	<0.001	0.34	0.98	(-1.58, 2.27)	0.725
**Autumn**	23.93	2.81	(18.40, 29.46)	<0.001	24.69	3	(18.79, 30.58)	<0.001	0.26	0.21	(-0.15, 0.66)	0.222	0.08	0.09	(-0.09, 0.25)	0.367	-0.39	0.51	(-1.40, 0.62)	0.448	2.45	0.95	(0.59, 4.31)	0.01
**Winter**	3.43	3.2	(-2.86, 9.71)	0.284	8.29	3.03	(2.34, 14.24)	0.006	0.05	0.22	(-0.38, 0.47)	0.828	0	0.09	(-0.18, 0.17)	0.992	-2.04	0.62	(-3.26, -0.83)	0.001	3.76	1.2	(1.41, 6.12)	0.002
**Constant**	316.6	4.95	(306.86, 326.33)	<0.001	294.85	5.82	(283.40, 306.29	<0.001	10.48	0.29	(9.91, 11.06)	<0.001	3.63	0.2	(3.24, 4.02)	<0.001	97.07	1.41	(94.31, 99.83)	<0.001	148.19	2.26	(143.74, 152.6	<0.001

NB: Except where specified, coefficients relate to effects in the baseline categories of all variables (pre-pandemic, spring, living alone, IMD quintile 1)

McElroy E, *et al. BMJ Ment Health* 2023; 26:e300842. doi: 10.1136/bmjment-2023-300842

Table S15. Region-stratified estimates from EHR analyses

	Anxiety				Depression				Eating disorder				OCD				Self harm				Severe mental illness			
	Coefficient	SE	95% CI	P value	Coefficient	SE	95% CI	P value	Coefficient	SE	95% CI	P value	Coefficient	SE	95% CI	P value	Coefficient	SE	95% CI	P value	Coefficient	SE	95% CI	P value
**Pre-pandemic effects**																								
East midlands, not living alone	-67.3	12.38	(-91.60, -43.00)	<0.001	-60.55	14.8	(-89.61, -31.50)	<0.001	3.3	0.58	(2.15, 4.44)	<0.001	-0.04	0.35	(-0.72, 0.64)	0.916	-36.67	1.08	(-38.79, -34.56)	<0.001	6.96	2.88	(1.31, 12.60)	0.016
East, living alone	-135.7	5.33	(-146.15, -125.25	<0.001	-132.77	6.46	(-145.46, -120.0	<0.001	-1.26	0.35	(-1.95, -0.57)	<0.001	-1.18	0.27	(-1.70, -0.66)	<0.001	-49.06	1	(-51.02, -47.09)	<0.001	-57.05	2.01	(-61.01, -53.10)	<0.001
East, not living alone	28.72	6.45	(16.06, 41.39)	<0.001	-3.41	8.78	(-20.65, 13.83)	0.698	4.34	0.59	(3.19, 5.50)	<0.001	-0.11	0.34	(-0.78, 0.57)	0.757	2.03	1.64	(-1.18, 5.25)	0.215	-12.31	3.45	(-19.08, -5.54)	<0.001
London, living alone	6.36	5.36	(-4.16, 16.88)	0.236	-38.16	6.69	(-51.29, -25.03)	<0.001	-1.06	0.41	(-1.85, -0.26)	0.01	0	0.3	(-0.59, 0.59)	0.997	-30.02	1.14	(-32.25, -27.78)	<0.001	-62.74	1.97	(-66.61, -58.87)	<0.001
London, not living alone	48.82	5.76	(37.51, 60.13)	<0.001	33.24	8.13	(17.28, 49.19)	<0.001	-0.32	0.49	(-1.29, 0.64)	0.511	-0.23	0.29	(-0.79, 0.33)	0.422	9.89	1.57	(6.81, 12.98)	<0.001	0.73	2.99	(-5.13, 6.59)	0.807
North East, living alone	55.74	6.09	(43.80, 67.69)	<0.001	5.77	7.01	(-7.99, 19.53)	0.411	-2.3	0.34	(-2.96, -1.63)	<0.001	-0.17	0.28	(-0.72, 0.37)	0.533	-24.43	1.12	(-26.64, -22.23)	<0.001	-59.25	2.04	(-63.26, -55.24)	<0.001
North East, not living alone	-37.22	5.04	(-47.11, -27.33)	<0.001	-59.83	6.67	(-72.93, -46.73)	<0.001	3.37	0.46	(2.47, 4.27)	<0.001	0.01	0.34	(-0.67, 0.69)	0.979	-4.64	1.62	(-7.81, -1.46)	0.004	-4.82	3	(-10.70, 1.07)	0.109
North West, living alone	-54.61	5.27	(-64.95, -44.27)	<0.001	-72	6.46	(-84.68, -59.32)	<0.001	-0.23	0.37	(-0.95, 0.49)	0.525	-0.04	0.27	(-0.57, 0.50)	0.89	-31.82	1.21	(-34.20, -29.45)	<0.001	-60.07	2.04	(-64.07, -56.07)	<0.001
North West, not living alone	-76.31	4.93	(-85.99, -66.62)	<0.001	-69.5	7.3	(-83.84, -55.17)	<0.001	0.79	0.43	(-0.06, 1.64)	0.069	-0.68	0.32	(-1.30, -0.06)	0.032	-13.44	1.09	(-15.59, -11.29)	<0.001	-14.02	3.05	(-20.01, -8.03)	<0.001
South East, living alone	-81.61	4.66	(-90.76, -72.46)	<0.001	-79.48	6.66	(-92.56, -66.40)	<0.001	-0.91	0.35	(-1.59, -0.23)	0.008	-0.96	0.26	(-1.46, -0.45)	<0.001	-33.75	1.04	(-35.78, -31.71)	<0.001	-63.85	2.11	(-67.98, -59.71)	<0.001
South East, not living alone	-4.37	7.57	(-19.22, 10.48)	0.564	-14.56	10	(-34.19, 5.07)	0.146	2.89	0.72	(1.48, 4.31)	<0.001	-0.55	0.4	(-1.34, 0.23)	0.167	-16.52	1.53	(-19.52, -13.53)	<0.001	6.68	2.87	(1.06, 12.31)	0.02
South West, living alone	-2.14	5.4	(-12.74, 8.45)	0.692	-42.45	7.62	(-57.40, -27.50)	<0.001	0.07	0.59	(-1.08, 1.22)	0.902	-0.22	0.32	(-0.85, 0.42)	0.502	-33.18	1.27	(-35.68, -30.68)	<0.001	-50.85	2.04	(-54.86, -46.84)	<0.001
South West, not living alone	17.85	5.74	(6.58, 29.12)	0.002	6.88	7.06	(-6.98, 20.75)	0.33	-0.17	0.47	(-1.09, 0.74)	0.711	-0.85	0.28	(-1.40, -0.29)	0.003	-1.73	1.19	(-4.07, 0.61)	0.147	0.09	2.93	(-5.67, 5.84)	0.977
West Midlands, living alone	2.01	5.26	(-8.31, 12.32)	0.703	-27.66	6.5	(-40.43, -14.89)	<0.001	-2.86	0.34	(-3.53, -2.19)	<0.001	-0.7	0.26	(-1.20, -0.20)	0.007	-31.4	0.96	(-33.29, -29.51)	<0.001	-58.61	2.09	(-62.73, -54.50)	<0.001
West Midlands, not living alone	-10.58	4.92	(-20.23, -0.92)	0.032	-17.99	7.04	(-31.81, -4.16)	0.011	-2.41	0.33	(-3.06, -1.75)	<0.001	-0.39	0.26	(-0.90, 0.11)	0.128	-29.1	1.11	(-31.28, -26.92)	<0.001	-59.06	1.95	(-62.90, -55.23)	<0.001
Yorkshire and The Humber, living alone	-24.35	5.3	(-34.75, -13.95)	<0.001	-41.75	7.11	(-55.71, -27.79)	<0.001	1.03	0.39	(0.27, 1.79)	0.008	0.48	0.31	(-0.13, 1.09)	0.126	-7.03	1.11	(-9.21, -4.85)	<0.001	3.14	3.02	(-2.79, 9.08)	0.299
Yorkshire and The Humber, not living alor	-48.11	5.28	(-58.47, -37.74)	<0.001	-68.8	6.39	(-81.34, -56.25)	<0.001	-1.82	0.32	(-2.45, -1.19)	<0.001	-0.37	0.26	(-0.87, 0.13)	0.151	-35.17	1.08	(-37.29, -33.06)	<0.001	-59.65	2.11	(-63.78, -55.51)	<0.001
**Effect of the pandemic in those living a**	-70.19	5.78	(-81.54,-58.84)	<0.001	-100.93	6.43	(-113.56,-88.31)	<0.001	0.1	0.55	(-0.98,1.17)	0.861	-1.21	0.27	(-1.74,-0.68)	<0.001	-3.87	2.23	(-8.25,0.51)	0.083	-7.06	2.75	(-12.46,-1.66)	0.01
**Interaction effects during pandemic**																								
East midlands, not living alone	16.09	8.18	(0.03, 32.14)	0.05	24.24	8.49	(7.57, 40.91)	0.004	0.13	0.62	(-1.08, 1.34)	0.833	0.44	0.32	(-0.18, 1.06)	0.167	6.75	2.54	(1.76, 11.73)	0.008	4.06	2.96	(-1.76, 9.87)	0.171
East, living alone	12.71	7.68	(-2.37, 27.79)	0.098	24.45	8.13	(8.48, 40.42)	0.003	-0.43	0.67	(-1.74, 0.87)	0.515	-0.21	0.35	(-0.89, 0.48)	0.552	-4.79	2.71	(-10.11, 0.54)	0.078	-1.45	4.31	(-9.90, 7.00)	0.737
East, not living alone	32.56	8.52	(15.84, 49.27)	<0.001	51.47	7.89	(35.98, 66.96)	<0.001	0.31	0.65	(-0.98, 1.59)	0.64	0.65	0.3	(0.06, 1.23)	0.031	4.43	2.41	(-0.31, 9.16)	0.067	3.55	3.07	(-2.48, 9.58)	0.248
London, living alone	169.32	17.14	(135.69, 202.96)	<0.001	194.41	19.07	(156.98, 231.84	<0.001	2.12	1.05	(0.07, 4.18)	0.043	3.27	0.53	(2.23, 4.32)	<0.001	3.3	2.56	(-1.71, 8.32)	0.197	-5.1	4.09	(-13.13, 2.94)	0.213
London, not living alone	81.42	8.31	(65.12, 97.72)	<0.001	106.16	8.36	(89.74, 122.57)	<0.001	0.75	0.74	(-0.70, 2.21)	0.31	1.45	0.32	(0.83, 2.07)	<0.001	4.57	2.37	(-0.08, 9.22)	0.054	4.38	3.09	(-1.68, 10.45)	0.156
North East, living alone	-4.56	8.66	(-21.56, 12.44)	0.599	6.03	9.92	(-13.43, 25.50)	0.543	-0.74	1.23	(-3.15, 1.68)	0.549	0.24	0.41	(-0.57, 1.05)	0.561	-8.67	3.27	(-15.10, -2.25)	0.008	11.63	4.67	(2.47, 20.79)	0.013
North East, not living alone	28.12	8.82	(10.82, 45.43)	0.001	50.67	8.92	(33.16, 68.17)	<0.001	-0.25	0.77	(-1.76, 1.26)	0.748	0.25	0.38	(-0.49, 0.99)	0.509	4.43	2.46	(-0.41, 9.27)	0.073	6.04	3.03	(0.09, 11.99)	0.047
North West, living alone	-0.65	9.39	(-19.08, 17.79)	0.945	-3.07	9.72	(-22.15, 16.00)	0.752	-0.16	0.79	(-1.70, 1.38)	0.84	0.78	0.38	(0.03, 1.53)	0.042	-5.19	3.03	(-11.14, 0.76)	0.087	-2.66	4.48	(-11.46, 6.13)	0.552
North West, not living alone	21.19	10.68	(0.24, 42.15)	0.047	35.16	9.8	(15.93, 54.39)	<0.001	0.72	0.67	(-0.59, 2.03)	0.282	0.51	0.34	(-0.16, 1.19)	0.134	6.56	2.51	(1.63, 11.50)	0.009	3.31	3.04	(-2.67, 9.28)	0.278
South East, living alone	25.83	8.99	(8.17, 43.48)	0.004	40.27	8.39	(23.81, 56.73)	<0.001	-1.22	0.87	(-2.94, 0.49)	0.162	0.68	0.44	(-0.19, 1.54)	0.125	0.47	3.38	(-6.16, 7.11)	0.889	-1.3	4.18	(-9.51, 6.92)	0.757
South East, not living alone	42.22	9.31	(23.95, 60.50)	<0.001	55.1	8	(39.41, 70.80)	<0.001	0.91	0.73	(-0.52, 2.34)	0.21	0.7	0.33	(0.05, 1.34)	0.034	6.04	2.44	(1.25, 10.83)	0.014	3.33	2.97	(-2.50, 9.17)	0.263
South West, living alone	35.69	7.33	(21.30, 50.08)	<0.001	41.17	7.97	(25.53, 56.81)	<0.001	-0.08	0.8	(-1.65, 1.49)	0.922	0.26	0.37	(-0.47, 0.98)	0.486	-2.58	2.67	(-7.83, 2.67)	0.335	1.17	4.12	(-6.92, 9.27)	0.776
South West, not living alone	44.96	7.85	(29.56, 60.36)	<0.001	54.19	7.77	(38.94, 69.44)	<0.001	0.72	0.69	(-0.63, 2.08)	0.295	0.7	0.31	(0.08, 1.31)	0.026	4.58	2.37	(-0.08, 9.23)	0.054	5.12	3.04	(-0.84, 11.08)	0.092
West Midlands, living alone	7.28	9.61	(-11.59, 26.14)	0.449	14.24	10.62	(-6.61, 35.09)	0.18	-0.35	1.2	(-2.71, 2.01)	0.77	0.63	0.5	(-0.34, 1.61)	0.204	-0.99	3.01	(-6.89, 4.91)	0.742	1	5.4	(-9.60, 11.60)	0.853
West Midlands, not living alone	13.81	8.04	(-1.98, 29.60)	0.087	39.22	8.68	(22.18, 56.27)	<0.001	0.33	0.81	(-1.26, 1.91)	0.687	0.66	0.36	(-0.05, 1.37)	0.067	3.25	2.53	(-1.71, 8.21)	0.198	3.82	3.12	(-2.30, 9.95)	0.221
Yorkshire and The Humber, living alone	19.89	8.47	(3.26, 36.52)	0.019	14.94	8.56	(-1.86, 31.74)	0.081	0.28	0.82	(-1.32, 1.88)	0.732	0.93	0.34	(0.27, 1.60)	0.006	-6.47	2.68	(-11.74, -1.20)	0.016	-1.62	4.44	(-10.35, 7.10)	0.715
Yorkshire and The Humber, not living alor	40.87	9.11	(22.98, 58.75)	<0.001	47.3	8.66	(30.30, 64.30)	<0.001	1.02	0.7	(-0.36, 2.40)	0.147	0.71	0.31	(0.11, 1.31)	0.021	2.57	2.34	(-2.02, 7.16)	0.272	3.31	3.02	(-2.61, 9.24)	0.273
**Summer**	6.42	2.42	(1.67, 11.17)	0.008	9.14	2.46	(4.31, 13.97)	<0.001	0.21	0.22	(-0.22, 0.65)	0.343	-0.04	0.09	(-0.22, 0.13)	0.62	2.81	0.48	(1.87, 3.75)	<0.001	0.07	0.83	(-1.55, 1.70)	0.929
**Autumn**	22.97	2.54	(17.98, 27.96)	<0.001	25.23	2.68	(19.97, 30.49)	<0.001	0.15	0.21	(-0.27, 0.57)	0.486	0.03	0.09	(-0.14, 0.21)	0.699	-0.74	0.46	(-1.64, 0.16)	0.108	2.16	0.84	(0.51, 3.80)	0.01
**Winter**	2.87	2.78	(-2.60, 8.33)	0.304	8.33	2.77	(2.89, 13.76)	0.003	-0.04	0.22	(-0.46, 0.39)	0.866	-0.06	0.09	(-0.24, 0.12)	0.511	-2.21	0.51	(-3.22, -1.20)	<0.001	3.07	1	(1.10, 5.04)	0.002
**Constant**	263.1	4.5	(254.27, 271.94)	<0.001	237.86	6.27	(225.55, 250.18	<0.001	8.44	0.33	(7.79, 9.09)	<0.001	3.46	0.25	(2.98, 3.94)	<0.001	59.53	0.91	(57.75, 61.31)	<0.001	90.35	1.94	(86.55, 94.15)	<0.001
**NB: Except where specified, coefficients relate to effects in the baseline categories of all variables (pre-pandemic, spring, living alone, East)**																								

Table S16. Shielded-stratified estimates from EHR analyses

	Anxiety				Depression				Eating disorder				OCD				Self harm				Severe mental illness			
	Coefficient	SE	95% CI	P value	Coefficient	SE	95% CI	P value	Coefficient	SE	95% CI	P value	Coefficient	SE	95% CI	P value	Coefficient	SE	95% CI	P value	Coefficient	SE	95% CI	P value
**Pre-pandemic effects**																								
Not shielding, not living alone	-17.97	3.66	(-25.20, -10.75)	<0.001	-28.01	3.71	(-35.33, -20.69)	<0.001	-2.84	0.2	(-3.23, -2.44)	<0.001	-0.44	0.12	(-0.68, -0.19)	<0.001	-24.88	0.89	(-26.64, -23.12)	<0.001	-49.6	1.88	(-53.30, -45.90)	<0.001
Shielding, living alone	-38.19	3.7	(-45.49, -30.89)	<0.001	-11	5.62	(-22.10, 0.10)	0.052	3.65	0.48	(2.71, 4.60)	<0.001	-1.52	0.16	(-1.82, -1.21)	<0.001	13.64	1.22	(11.24, 16.04)	<0.001	110.49	5.29	(100.05, 120.93	<0.001
Shielding, not living alone	-41.05	3.71	(-48.37, -33.73)	<0.001	-15.79	4.71	(-25.09, -6.50)	0.001	2.32	0.36	(1.61, 3.03)	<0.001	-1.26	0.13	(-1.52, -1.01)	<0.001	-16.94	0.8	(-18.52, -15.36)	<0.001	15.12	2.82	(9.56, 20.68)	<0.001
**Effect of the pandemic in tho**	-45.57	5.34	(-56.10,-35.03)	<0.001	-66.38	4.89	(-76.03,-56.73)	<0.001	-0.15	0.42	(-0.99,0.68)	0.721	-0.67	0.15	(-0.96,-0.38)	<0.001	-7.28	1.39	(-10.03,-4.53)	<0.001	-8.58	2.46	(-13.44,-3.71)	0.001
**Interaction effects during pandemic**																								
Not shielding, not living alone	7.66	8.13	(-8.39, 23.72)	0.347	13.99	6.96	(0.25, 27.73)	0.046	0.76	0.54	(-0.31, 1.83)	0.163	0.09	0.18	(-0.27, 0.45)	0.629	8.22	1.66	(4.94, 11.51)	<0.001	5.63	2.82	(0.07, 11.19)	0.047
Shielding, living alone	26.48	6.63	(13.40, 39.56)	<0.001	-0.35	7.42	(-14.99, 14.30)	0.963	2.33	0.73	(0.89, 3.76)	0.002	0.32	0.22	(-0.12, 0.75)	0.152	3.29	2.39	(-1.43, 8.01)	0.17	17.48	7.83	(2.03, 32.92)	0.027
Shielding, not living alone	31.99	7.48	(17.24, 46.75)	<0.001	9.99	6.9	(-3.63, 23.60)	0.15	0.72	0.62	(-0.51, 1.95)	0.249	0.23	0.19	(-0.14, 0.60)	0.227	8.36	1.76	(4.88, 11.83)	<0.001	5.78	4.27	(-2.66, 14.21)	0.178
**Summer**	7.76	3.94	(-0.02, 15.53)	0.05	11.14	4.28	(2.69, 19.59)	0.01	0.75	0.37	(0.03, 1.47)	0.041	0.05	0.1	(-0.15, 0.24)	0.637	3.88	0.98	(1.94, 5.82)	<0.001	3.07	2.99	(-2.84, 8.97)	0.307
**Autumn**	22.08	3.92	(14.34, 29.82)	<0.001	24.33	4.66	(15.13, 33.52)	<0.001	0.62	0.37	(-0.10, 1.35)	0.091	0.08	0.1	(-0.12, 0.28)	0.423	0.08	0.91	(-1.71, 1.87)	0.929	7.19	2.96	(1.35, 13.03)	0.016
**Winter**	1.59	4.58	(-7.46, 10.63)	0.729	7.9	4.48	(-0.94, 16.74)	0.08	0.23	0.39	(-0.54, 1.01)	0.554	0.05	0.11	(-0.16, 0.27)	0.629	-2.19	1.01	(-4.19, -0.19)	0.032	7.9	3.51	(0.96, 14.83)	0.026
**Constant**	251.41	3.65	(244.21, 258.61)	<0.001	213.94	4.28	(205.48, 222.39	<0.001	8.83	0.3	(8.25, 9.41)	<0.001	3.41	0.12	(3.18, 3.64)	<0.001	50.53	0.87	(48.81, 52.24)	<0.001	72.23	2.55	(67.21, 77.26)	<0.001
**NB: Except where specified, coefficients relate to effects in the baseline categories of all variables (pre-pandemic, spring, living alone, not shielding)**																								

Table S17. Previous mental illness-stratified estimates from EHR analyses

	Anxiety				Depression			
	Coefficient	SE	95% CI	P value	Coefficient	SE	95% CI	P value
**Pre-pandemic effects**								
No previous mental illness, not living alone	2.24	5.98	(-9.56, 14.05)	0.708	-3.95	5.46	(-14.72, 6.82)	0.47
Previous mental illness, living alone	907.96	13.31	(881.69, 934.23)	<0.001	809.36	16.47	(776.86, 841.85	<0.001
Previous mental illness, not living alone	943.07	12.03	(919.32, 966.82)	<0.001	785.88	14.91	(756.46, 815.29	<0.001
**Effect of the pandemic in those living alone**	-15.48	6.43	(-28.18,-2.78)	0.017	-31.69	6.01	(-43.55,-19.83)	<0.001
**Interaction effects during pandemic**								
No previous mental illness, not living alone	2.34	9.18	(-15.79, 20.46)	0.799	8.2	8.37	(-8.31, 24.70)	0.329
Previous mental illness, living alone	-188.7	20.92	(-229.98, -147.42	<0.001	-238.8	22.13	(-282.47, -195.1	<0.001
Previous mental illness, not living alone	-203.31	23.87	(-250.42, -156.20	<0.001	-241.42	22.62	(-286.07, -196.7	<0.001
**Summer**	14.67	12.41	(-9.82, 39.15)	0.239	16.56	12.65	(-8.41, 41.52)	0.192
**Autumn**	47.36	13.56	(20.60, 74.12)	0.001	49.27	14.58	(20.50, 78.03)	0.001
**Winter**	1.32	13.8	(-25.92, 28.55)	0.924	10.99	13.11	(-14.88, 36.85)	0.403
**Constant**	100.63	9.66	(81.57, 119.70)	<0.001	81.2	9.71	(62.03, 100.36)	<0.001

	Eating disorder				OCD			
	Coefficient	SE	95% CI	P value	Coefficient	SE	95% CI	P value
**Pre-pandemic effects**								
No previous mental illness, not living alone	-0.43	0.24	(-0.91, 0.05)	0.078	-0.03	0.06	(-0.15, 0.10)	0.683
Previous mental illness, living alone	46.6	1.1	(44.43, 48.78)	<0.001	14.41	0.46	(13.51, 15.31	<0.001
Previous mental illness, not living alone	35.75	0.59	(34.59, 36.91)	<0.001	14.82	0.35	(14.12, 15.51	<0.001
**Effect of the pandemic in those living alone**	-0.08	0.27	(-0.62,0.45)	0.766	-0.22	0.08	(-0.38,-0.06)	0.006
**Interaction effects during pandemic**								
No previous mental illness, not living alone	0.14	0.37	(-0.60, 0.87)	0.716	0.08	0.09	(-0.11, 0.27)	0.406
Previous mental illness, living alone	0.46	2.14	(-3.76, 4.68)	0.831	-2.69	0.63	(-3.94, -1.43)	<0.001
Previous mental illness, not living alone	2.99	1.86	(-0.68, 6.65)	0.11	-3.49	0.55	(-4.57, -2.40)	<0.001
**Summer**	1.41	1.12	(-0.79, 3.61)	0.208	-0.02	0.34	(-0.68, 0.64)	0.953
**Autumn**	0.54	1.03	(-1.50, 2.57)	0.605	0.05	0.34	(-0.62, 0.71)	0.89
**Winter**	-0.13	1.11	(-2.33, 2.07)	0.908	-0.04	0.33	(-0.69, 0.61)	0.905
**Constant**	2.12	0.73	(0.67, 3.57)	0.004	1.08	0.22	(0.64, 1.51)	<0.001

	Self harm				Severe mental illness			
	Coefficient	SE	95% CI	P value	Coefficient	SE	95% CI	P value
**Pre-pandemic effects**								
No previous mental illness, not living alone	-3.55	1.95	(-7.39, 0.29)	0.07	-9.96	1.44	(-12.81, -7.11)	<0.001
Previous mental illness, living alone	264.23	4.25	(255.83, 272.6	<0.001	483.38	8.53	(466.54, 500.2	<0.001
Previous mental illness, not living alone	135.55	2.46	(130.68, 140.4	<0.001	189.25	3.74	(181.86, 196.6	<0.001
**Effect of the pandemic in those living alone**	-1.66	1.92	(-5.46,2.13)	0.388	-2.66	1.52	(-5.67,0.35)	0.083
**Interaction effects during pandemic**								
No previous mental illness, not living alone	1.58	2.77	(-3.88, 7.03)	0.57	1.61	2.1	(-2.53, 5.76)	0.444
Previous mental illness, living alone	-38.59	8.33	(-55.03, -22.16)	<0.001	-36.73	14.39	(-65.13, -8.32)	0.012
Previous mental illness, not living alone	4.3	4.28	(-4.16, 12.75)	0.317	-20.27	6.67	(-33.44, -7.10)	0.003
**Summer**	8.46	3.13	(2.29, 14.64)	0.007	2.49	5.78	(-8.92, 13.90)	0.667
**Autumn**	-1.13	2.59	(-6.23, 3.97)	0.663	8.44	5.64	(-2.69, 19.56)	0.136
**Winter**	-6.72	3.26	(-13.15, -0.29)	0.04	9.65	6.91	(-3.98, 23.28)	0.164
**Constant**	12.17	2.09	(8.05, 16.29)	<0.001	11.38	4.17	(3.16, 19.60)	0.007

NB: Except where specified, coefficients relate to effects in the baseline categories of all variables (pre-pandemic, spring, living alone, no previous mental illness)

68

Table S18. Heterogeneity between estimates of psychological distress explained by cohort differences (meta-regression analyses)

Measure	Timepoint	Heterogeneity (I^2)	Heterogeneity Explained (%)
**Time between pre-pandemic and first pandemic measure**	0	65.7	-7.2
	1	89.2	-2.0
	2	85.9	-3.1
	3	80.2	-4.2
**General population representativeness**	0	58.0	0.6
	1	88.0	-0.8
	2	82.7	0.1
	3	77.7	-1.6
**Type of measure for mental health used**	0	19.4	39.1
	1	82.0	5.2
	2	79.9	2.9
	3	71.8	4.2

Table S19 Leave-One-Out Analysis

Meta-analysed standardised mean differences for psychological distress when named cohort is removed from analysis.

Time Period 0			
Cohort	SMD	Lower CI	Upper CI
All Included	0.09	0.04	0.14
ALSPAC-G0	0.08	0.03	0.14
ALSPAC-G1	0.09	0.04	0.14
BCS	0.10	0.05	0.15
ELSA	0.07	0.03	0.12
GS	0.09	0.04	0.15
NCDS	0.09	0.04	0.15
NS	0.10	0.04	0.15
NSHD	0.10	0.05	0.16
TwinsUK	0.10	0.04	0.15
USOC	0.11	0.06	0.16

Time Period 1			
Cohort	SMD	Lower CI	Upper CI
All Included	0.19	0.09	0.29
ALSPAC-G0	0.15	0.07	0.23
ALSPAC-G1	0.18	0.08	0.29
BCS	0.22	0.12	0.31
ELSA	0.18	0.07	0.29
GS	0.20	0.09	0.31
NCDS	0.21	0.10	0.31
NS	0.18	0.08	0.28
NSHD	0.18	0.08	0.29
TwinsUK	0.21	0.11	0.31
USOC	0.19	0.08	0.31

Time Period 2			
Cohort	SMD	Lower CI	Upper CI
All Included	0.15	0.06	0.25
ALSPAC-G0	0.11	0.05	0.18
ALSPAC-G1	0.15	0.05	0.25
BCS	0.17	0.07	0.27
ELSA*	0.15	0.06	0.25
GS	0.15	0.04	0.25
NCDS	0.17	0.07	0.27
NS	0.15	0.05	0.26
NSHD	0.15	0.05	0.25
TwinsUK	0.18	0.08	0.27
USOC	0.16	0.05	0.26

Time Period 3			
Cohort	SMD	Lower CI	Upper CI
All Included	0.15	0.07	0.22
ALSPAC-G0	0.12	0.06	0.19
ALSPAC-G1	0.14	0.06	0.21
BCS	0.15	0.07	0.23
ELSA	0.14	0.06	0.21
GS	0.14	0.07	0.22
NCDS	0.17	0.09	0.24
NS	0.15	0.07	0.23
NSHD	0.15	0.07	0.23
TwinsUK	0.17	0.10	0.24
USOC	0.15	0.07	0.23

* ELSA does not contain information for Time Period 2

McElroy E, *et al. BMJ Ment Health* 2023; 26:e300842. doi: 10.1136/bmjment-2023-300842

Figure S1. Results of meta-analyses in LPS

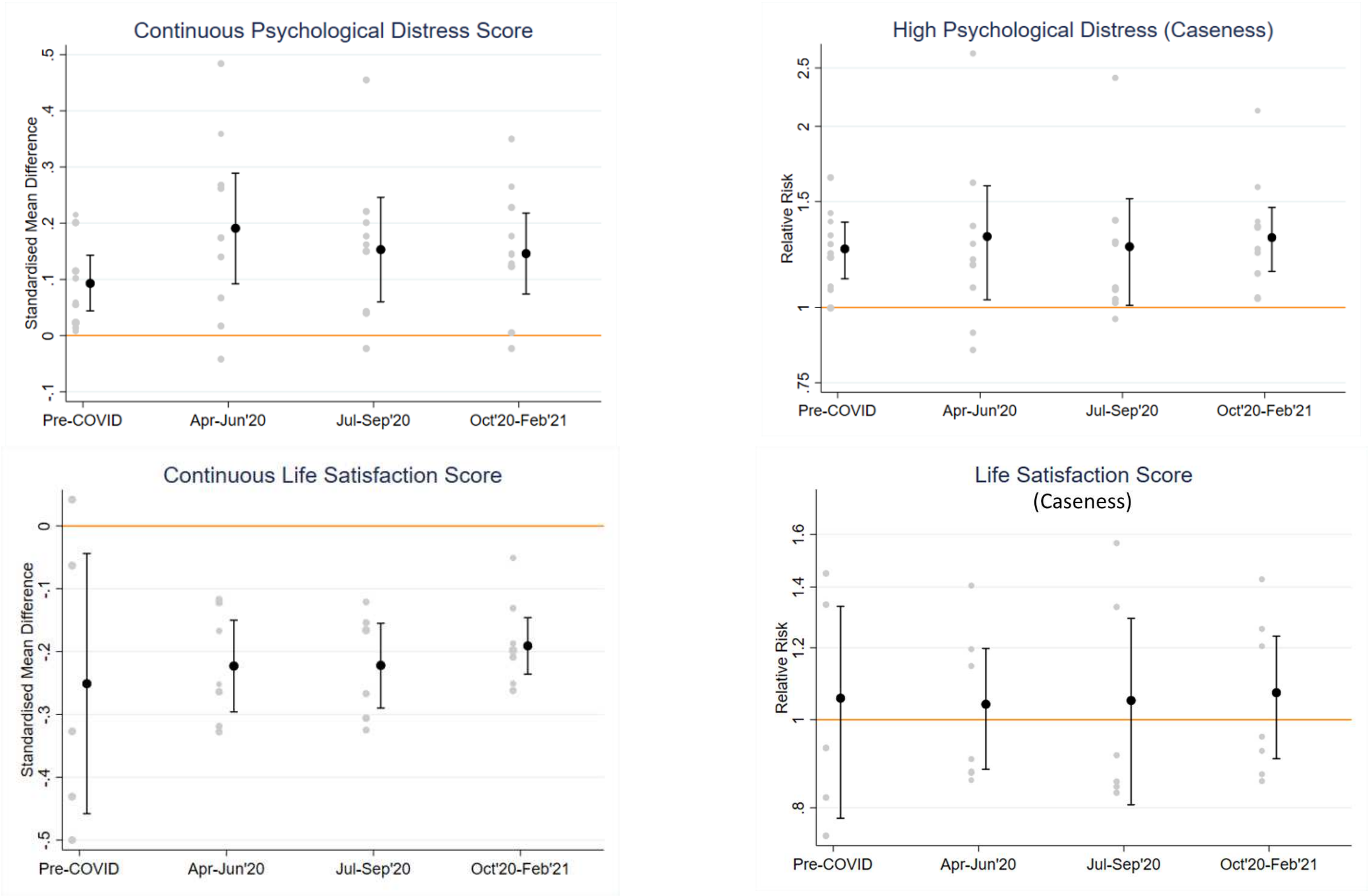

Supplement: Supplementary data [file bmjment-2023-300842supp001.pdf]
